# Integrated multi-omics analysis and experimental investigation of mitochondrial dynamics-related genes: molecular subtypes, immune landscape, and prognostic implications in lung adenocarcinoma

**DOI:** 10.3389/fimmu.2025.1585505

**Published:** 2025-05-29

**Authors:** Sixuan Wu, Junfan Pan, Lijun Zeng, Qihong Pan, Yao Yu, Jincan Lin, Jing Zhang, Yiling Jiang

**Affiliations:** ^1^ Department of Oncology, The First Affiliated Hospital, Hengyang Medical School, University of South China, Hengyang, Hunan, China; ^2^ Clinical Oncology School of Fujian Medical University, Fujian Cancer Hospital, Fuzhou, China; ^3^ Pu Ai Medical School, Shaoyang University, Shaoyang, Hunan, China; ^4^ Xiamen Critical Care Quality Control Center, Department of Intensive Care Unit, School of Medicine, The First Affiliated Hospital of Xiamen University, Xiamen University, Xiamen, China; ^5^ Department of Thoracic Oncology, Clinical Oncology School of Fujian Medical University, Fujian Cancer Hospital, Fuzhou, China

**Keywords:** LUAD, mitochondrial dynamics, prognostic model, tumor microenvironment, MTCH2

## Abstract

**Background:**

Lung adenocarcinoma (LUAD) is a common and aggressive subtype of lung cancer associated with poor clinical outcomes. The role of mitochondrial dynamics (MD)-related genes in tumor progression and immune regulation remains poorly understood.

**Methods:**

Data from public databases were integrated, and subtypes were classified based on 23 MD-related genes. A five-gene prognostic model was constructed. Associations between the model and immune infiltration, tumor mutational burden (TMB), tumor stemness, and drug sensitivity were analyzed. The function of the key gene MTCH2 was validated through *in vitro* experiments.

**Results:**

Two distinct MD molecular subtypes were identified, exhibiting significant differences in prognosis and immune characteristics. A corresponding risk score model was established. Patients in the low-risk group showed better prognosis and enhanced immune activity, whereas the high-risk group displayed higher TMB and stemness scores. Drug sensitivity analysis revealed distinct responses to chemotherapeutic agents such as cisplatin and docetaxel between risk groups. Functional assays demonstrated that MTCH2 knockout significantly inhibited LUAD cell proliferation, migration, and invasion, and induced G0/G1 phase arrest, suggesting that MTCH2 may act as a potential adverse prognostic marker.

**Conclusion:**

MD-related genes exhibit strong prognostic and immune subtyping value. The proposed risk model holds clinical potential, and MTCH2 may serve as a promising target for precision therapy in LUAD.

## Introduction

Lung adenocarcinoma (LUAD), recognized as the most common subtype of non-small cell lung cancer (NSCLC), significantly contributes to the global health crisis, accounting for nearly 40% of all lung cancer incidences (1–4). Despite notable progress in both diagnostic and therapeutic approaches, the overall prognosis for LUAD continues to be unfavorable, as evidenced by a 5-year survival rate that falls below 20% (5, 6). Current treatment options are frequently constrained by issues such as drug resistance and adverse side effects. Therefore, it is crucial to discover novel biomarkers and therapeutic targets that may enhance the clinical outcomes for patients with LUAD.

Mitochondria are incredibly versatile and adaptable organelles that are essential for cellular metabolism, the response to stress, and the regulation of homeostasis (7). Mitochondrial dynamics (MD) are primarily governed by fusion and fission (8), both of which are essential for maintaining mitochondrial homeostasis (9–11). Fusion mitigates cellular stress and facilitates functional complementation by merging partially damaged mitochondrial contents, whereas fission supports quality control by generating new mitochondria and isolating damaged ones from healthy counterparts (12, 13). In mammalian cells, the fusion process of the outer mitochondrial membrane is orchestrated by mitofusin-1 (MFN1) and mitofusin-2 (MFN2). In contrast, the fusion of the inner mitochondrial membrane is facilitated by the optic atrophy 1 (Opa1) protein (14). Mfn1 is predominantly expressed in the pancreas, heart, adrenal glands, liver, and testis, whereas Mfn2 is mainly expressed in the brain, skeletal muscle, heart, and brown adipose tissue (15, 16). OPA1 also exhibits high expression levels in the brain, retina, liver, heart, testis, and skeletal muscle (17, 18). Notably, the biology of OPA1 is complex, with eight isoforms generated in humans through alternative splicing, each exhibiting distinct functional activities (19). The primary proteins that are essential for the mitochondrial fission process include mitochondrial fission 1 protein (Fis1) and dynamin-related protein 1 (Drp1) (20, 21). Drp1, which possesses GTPase activity, is primarily localized in the cytoplasm and is widely distributed across various tissues (22). The equilibrium between mitochondrial fusion and fission plays a vital role in the bioenergetic functions of these organelles. This equilibrium can be disrupted by intracellular stressors or external factors, leading to excessive mitochondrial fission, which is characterized by a marked increase in Drp1 levels (23).

Abnormal expression of mitochondrial dynamin proteins has been observed in various human cancers, including lung cancer, bladder cancer, breast cancer, pancreatic cancer, colorectal cancer, ovarian cancer, and melanoma (24–31). In studies on lung cancer cells, mitochondrial fragmentation was strongly correlated with tumor phenotype and was correlated with elevated levels of Drp1 and its hyperactive phosphorylated form, Drp1P616 (24). Notably, the silencing of Drp1 leads to a decrease in cell proliferation and triggers apoptosis in lung cancer cells. Conversely, heightened activity of Drp1 facilitates the transformation of cells driven by oncogenic Ras (27, 32). Ras activation is further promoted by the upregulation of mitogen-activated protein kinase (MAPK), which phosphorylates Drp1 at serine 616. A multitude of investigations have shown that the modulation of mitochondrial fusion and fission plays a significant role in influencing tumor metabolism, cellular proliferation, metastasis, migration, and the preservation of tumor stem cell populations (33–37). Additionally, MD has been recognized as a significant contributor to the emergence of drug resistance in cancerous cells (38–41). Furthermore, MD profoundly influences immune surveillance within the tumor microenvironment (TME) (42–46). Therefore, further investigation into the role of MD in tumors is of significant importance.

In this study, the RNA expression profiles of MD-related genes in LUAD were systematically analyzed to investigate their roles in disease progression. Based on integrated data from public databases, distinct molecular subtypes were identified, and a prognostic model comprising five MD-related genes was developed. This model effectively stratified patients into high- and low-risk groups and demonstrated strong associations with tumor mutational burden (TMB), tumor stemness, and levels of immune cell infiltration-factors closely linked to tumor aggressiveness and therapeutic resistance. High-risk patients were found to exhibit elevated TMB and stemness scores, suggesting enhanced immune evasion and greater treatment challenges. Moreover, significant differences in drug response were observed between risk groups, indicating potential clinical relevance. The function of the key MD gene MTCH2 was further validated through *in vitro* experiments. MTCH2 knockdown markedly suppressed LUAD cell proliferation, migration, and invasion, and induced G0/G1 phase cell cycle arrest, indicating its potential role as a tumor-promoting gene and unfavorable prognostic factor. In summary, this study systematically elucidates the relevance of MD-related genes to LUAD prognosis, immune evasion, and drug resistance. It also highlights MTCH2 as a promising target for precision therapy, providing a theoretical foundation and practical direction for future research on LUAD molecular mechanisms and personalized treatment strategies.

## Materials and methods

### Data acquisition

The RNA expression profiles, somatic mutation data, and clinical characteristics of LUAD were sourced from the The Cancer Genome Atlas (TCGA) database. Additionally, an adequate number of samples, accompanied by comprehensive clinical information, were obtained from the GSE13213 dataset within the Gene Expression Omnibus (GEO) database. Data from 541 patients diagnosed with LUAD and 59 healthy controls sourced from the TCGA database were combined with information from 117 LUAD patients in the GSE13213 dataset to construct a detailed expression matrix. The integration and processing of these datasets were methodically executed with the aid of Strawberry Perl (version 5.30.0.1). To enhance the robustness and reliability of the analytical results, potential outliers in the TCGA and GEO datasets were systematically identified and processed during the data preprocessing stage. RNA expression data were first standardized and visualized using principal component analysis (PCA) to detect samples exhibiting expression patterns that deviated significantly from the main distribution. Additionally, the ComBat algorithm was applied to eliminate batch effects, thereby ensuring consistency of the expression matrix across multiple data sources. Informed by previous studies (47), 23 genes associated with MD were selected for analysis (**Supplementary Table S1**). The study’s methodological flowchart is presented in **Supplementary Figure S1**.

In addition, the researchers obtained tissue samples of LUAD and adjacent non-cancerous tissues from 30 lung cancer patients undergoing surgical treatment at Fujian Cancer Hospital. The sample collection process for all participants followed the relevant regulations of the hospital’s Ethics Committee and was approved by the hospital’s Ethics Committee (approval number: K2023-417-01). Prior to sample collection, all patients signed an informed consent form to ensure that they were fully aware of the purpose, process, and possible risks of the study.

### Consensus clustering analysis

The RNA sequences of MD genes in LUAD patients were analyzed using consensus clustering. The consensus clustering algorithm in the R package “ConsensusClusterPlus” (48) was employed to classify patients into distinct molecular subgroups. The ideal quantity of subclusters was determined through an evaluation of the consensus of the clusters, the cumulative distribution function (CDF) of the consensus, and the delta area. PCA was utilized to assess the classification of the subgroups, providing insight into the variability between them. Based on the consensus of MD genes, patients with LUAD in the TCGA cohort were categorized into two distinct clusters. Survival differences between the subgroups were evaluated using Kaplan-Meier analysis.

### Enrichment analysis

The clinical and pathological characteristics distinguishing the two subgroups of MD were analyzed and illustrated through the utilization of R packages, specifically “limma”, “pheatmap”, and “ggpubr”. A comprehensive analysis led to the identification of 217 differentially expressed genes (DEGs) linked to the MD subtypes, determined by an absolute log-fold change threshold (|log2FC| > 2) and a significance level with a P-value below 0.001, as detailed in **Supplementary Table S2**. Gene Ontology (GO) enrichment analysis (49) and Kyoto Encyclopedia of Genes and Genomes (KEGG) pathway analysis (50) were conducted on the DEGs from these two MD subgroups using the ‘clusterProfiler’ R package (51). Gene set variation analysis (GSVA) facilitates the comparative analysis of signaling pathways. Concurrently, to examine the functions and pathways associated with MTCH2 in LUAD, gene set enrichment analysis (GSEA) was utilized (52).

### Creation and assessment of the prognostic model

DEGs linked to OS were initially determined through univariate Cox regression analysis, applying a significance criterion of P < 0.05. Subsequently, Lasso Cox regression analysis was conducted utilizing the R package “glmnet” (53), which reduced the number of genes to 12. A subsequent multivariate Cox regression analysis was conducted, and five genes were selected for the construction of the prognostic model. To validate the model’s reliability, all LUAD patients were arbitrarily assigned to either the training or testing group, with each group containing 312 individuals. Based on the median risk score, patients were categorized into low-risk and high-risk groups. The prognostic efficacy of the model was evaluated in these groups through receiver operating characteristic (ROC) analysis and Kaplan-Meier survival analysis. To further enhance the clinical applicability of the model, clinical variables, including age, gender, N-stage, T-stage, and risk scores, were incorporated into a comprehensive nomogram, and the model’s predictive accuracy was assessed using calibration curves.

### Assessment of TME

To assess the distribution of stromal and immune cells within the TME and their potential influence on patient prognosis, the stromal and immune scores of each individual were determined utilizing the ESTIMATE algorithm. Meanwhile, the CIBERSORT method was used to quantify the infiltration of immune cells in samples from the low-risk and high-risk groups, thus revealing differences in the immune microenvironment across risk groups. In addition, the study further compared the ratios between five prognostic genes and 19 immune cell types to explore the correlation between these genes and immune cell infiltration and to assess their potential impact on patient prognosis.

### Analysis of TMB, tumor stemness score, somatic mutation profile, and drug sensitivity

Using the R package “maftools” (54), a mutation annotation format was generated to compare the somatic mutation profiles of LUAD patients. This analysis provided insights into the mutational landscape of LUAD and identified key genes potentially associated with prognosis. Additionally, the correlation between tumor stemness score, TMB, and risk score was examined to explore the potential impact of these factors on patient prognosis. To further evaluate drug sensitivity in LUAD patients, the pRRophetic software package, based on gene expression data, was employed to predict the half-maximal inhibitory concentration (IC50) of relevant drugs. This approach allowed for the quantification of drug potency and the comparison of drug sensitivity between high- and low-risk groups.

### Cell culture and transfection

BEAS-2, PC9, H1299, and A549 cell lines were obtained from ProCell (Wuhan, China) and are commonly used in oncology and immunology research. The cells were cultured under standard conditions (37°C, 5% CO2, and humidified atmosphere) in RPMI-1640 medium supplemented with 10% fetal bovine serum (FBS). To investigate the role of MTCH2, human MTCH2-targeted small interfering RNAs (siRNAs), designed by Hanbio Co. Ltd (Shanghai, China), were employed to knock down MTCH2 expression and examine its function in LUAD cells. Transient transfections were performed using Lipofectamine 3000 (Invitrogen, Carlsbad, CA, USA), following the manufacturer’s instructions. Cells were harvested 48–72 hours post-transfection. The siRNA sequences used were as follows: MTCH2si#1: 5’-GGACUUGUGAUUCCAUCAUA-3’; MTCH2si#2: 5’-GGAUUUUUUGGCGAUUGAUUG-3’; MTCH2si#3: 5’-CCUUAUCCCCAAUAUAAUACG-3’; and sicontrol: 5’-UUCUCCGAACGUGUCACGUTT-3’.

### Real-time quantitative reverse transcription polymerase chain reaction (qRT-PCR)

Total RNA was extracted utilizing the TRIzol reagent (Invitrogen, USA), followed by the synthesis of complementary DNA employing the PrimeScript RT kit (Takara). The qRT-PCR was performed utilizing the SYBR Green assay from Takara. The analysis of qRT-PCR data was performed utilizing the 2-ΔΔCt approach, where β-actin served as the internal control reference. The specific primers utilized in this study were as follows: MTCH2-F: 5’-TGGTACAGTTCATTGGCAGAG-3’; MTCH2-R: 5’-GCATAGGTATTGACGAGGTAGG-3’; β-actin-F: 5’-GAGAAAATCTGGCACCACACC-3’; and β-actin-R: 5’-GGATAGCACAGCCTGGATAGCAA-3’.

### Western blot

Intracellular proteins were isolated utilizing RIPA lysis buffer, which was enhanced with phosphatase inhibitor (P1081, Beyotime, China) and 1% protease inhibitor (P1005, Beyotime, China) to prevent phosphorylation and protein degradation. The concentration of protein in the extract was assessed utilizing the bicinchoninic acid (BCA) assay (Beyotime Biotechnology), based on the absorbance measurement. Subsequently, the complete cell lysate underwent separation of proteins based on molecular weight using 4%–20% gradient sodium dodecyl sulfate-polyacrylamide gel electrophoresis (SDS-PAGE). Following electrophoresis, the proteins were moved from the gel to a polyvinylidene fluoride (PVDF) membrane for immunodetection. In order to prevent the non-specific binding of antibodies, the membrane was subjected to incubation at room temperature for a duration of one hour in a solution of 5% skim milk diluted in TBST buffer. Finally, the membrane was subjected to an overnight incubation at 4°C with the primary antibody diluted as per the manufacturer’s guidelines to guarantee specific interaction with the target protein. Following this incubation, the membranes were incubated with secondary antibodies conjugated to horseradish peroxidase (HRP) for one hour. Visualization of protein bands was achieved using a chemiluminescent HRP substrate in conjunction with an imaging system (Chemidoc, Bio-Rad). The primary antibodies employed in the study, at specified dilutions, included MTCH2 (Cat No: 16888-1-AP, 1:1000; Proteintech, Wuhan, China) and α-tubulin (Cat No: 11224-1-AP, 1:1000; Proteintech, Wuhan, China).

### Cell proliferation and colony formation assays

In order to evaluate cellular proliferation, a total of 1 × 10^4 cells were introduced into each well of a 24-well plate and subsequently cultured in RPMI 1640 medium that was enriched with 10% fetal bovine serum (FBS). The cells were maintained in a humidified incubator set at 37°C with an atmosphere containing 5% CO2. Samples of the cells were obtained at 24, 48, and 72 hours following inoculation, and their optical density (OD) values were assessed using a cell counting kit (CCK)-8. Changes in absorbance were used as an indicator of cell proliferation. To further evaluate the proliferative capacity, a total of 500 cells were seeded into each well of 6-well plates and incubated for 10 days. During this period, the medium was replaced periodically, and the cell growth was monitored. Upon completion of the culture duration, the cells were subjected to fixation using formaldehyde to maintain their morphological characteristics and subsequently stained with a crystal violet solution (Sigma-Aldrich). Cell counting was then performed using an inverted microscope, and images were captured to document cell growth. To guarantee the dependability, consistency, and strength of the findings, each experiment was conducted on three separate occasions.

### Wound-healing assay

Cells were plated in 6-well plates at a density of 1 × 10^5 cells for each well. Subsequently, the cell monolayer was delicately scraped using the end of a 10 μL pipette tip to create a wound and simulate a traumatized environment, enabling the observation of cell migration. The scraping procedure was carried out with precision to ensure uniformity and consistency in wound width, which is critical for reproducibility and comparability of the results. Subsequent to the scraping process, any remaining cellular debris was eliminated through washing the wells with PBS to reduce its possible impact on cell migration. Microscopic observations were conducted at two time points: 0 hours and 24 hours. Images were captured at both time points, and three replicate wells were included in each experiment to ensure statistical reliability and data stability.

### Transwell assays

To perform the cell migration assay, a total of 2 × 10^4 suspension cells were initially introduced into the upper chamber of the transwell apparatus (BD Biosciences, USA), utilizing 200 μL of medium devoid of serum. For the invasion assay, 50 μL of Matrigel diluted 1:8 was uniformly applied to the lower chamber and incubated at 37°C for 4 hours to allow the Matrigel to solidify, mimicking the extracellular matrix environment. Subsequently, a total of 5 × 10^4 cells were resuspended in 200 μL of serum-free medium and subsequently introduced into the upper chamber that contained the solidified Matrigel. To provide adequate nutritional support, a volume of 600 μL of medium, enriched with 10% fetal bovine serum (FBS), was introduced into the lower chamber to facilitate cell proliferation and migration. After 24 hours of incubation, cells in the lower chamber were fixed with formaldehyde and subsequently stained with crystal violet. The migratory capacity of the cells was assessed by inverted microscopy, with five randomly selected fields photographed and analyzed for cell count. Three replicate wells were used for each experimental condition to ensure the reliability and statistical significance of the results.

### Cell cycle assay

After trypsin digestion and dissociation, the transfected cells (1 × 10^6 cells per well) were fixed in a solution with a concentration of 75% ethanol and kept at a temperature of 4°C for an overnight incubation period. The fixed cells were then centrifuged at 1000 rpm for 5 minutes to remove the ethanol and wash the cells. The cells were subsequently treated with 5% propidium iodide solution, which binds to DNA in the nucleus, allowing for the identification of different cell cycle phases. Following staining, the cells were resuspended and prepared for analysis via flow cytometry. Cell cycle distribution was analyzed utilizing a CytoFLEX flow cytometer (USA), which captures both scatter and fluorescence signals to accurately differentiate between the G0/G1, S, and G2/M phases. Three replicate wells were included for each condition to ensure the reliability and statistical significance of the results.

### Statistical analysis

Statistical analyses were conducted using R software (version 4.3.3) and GraphPad Prism 9. Between-group comparisons were performed using two-tailed unpaired Student’s t-tests. Survival analyses were carried out using the Kaplan-Meier method, with statistical significance assessed by the log-rank test. Prognostic factors were evaluated through univariate and multivariate Cox proportional hazards regression analyses. For differential expression and enrichment analyses, P values were adjusted for multiple comparisons using the Benjamini-Hochberg method to control the false discovery rate. A P value < 0.05 was considered statistically significant.

## Results

### Transcriptional and genetic changes

**Figure 1:**
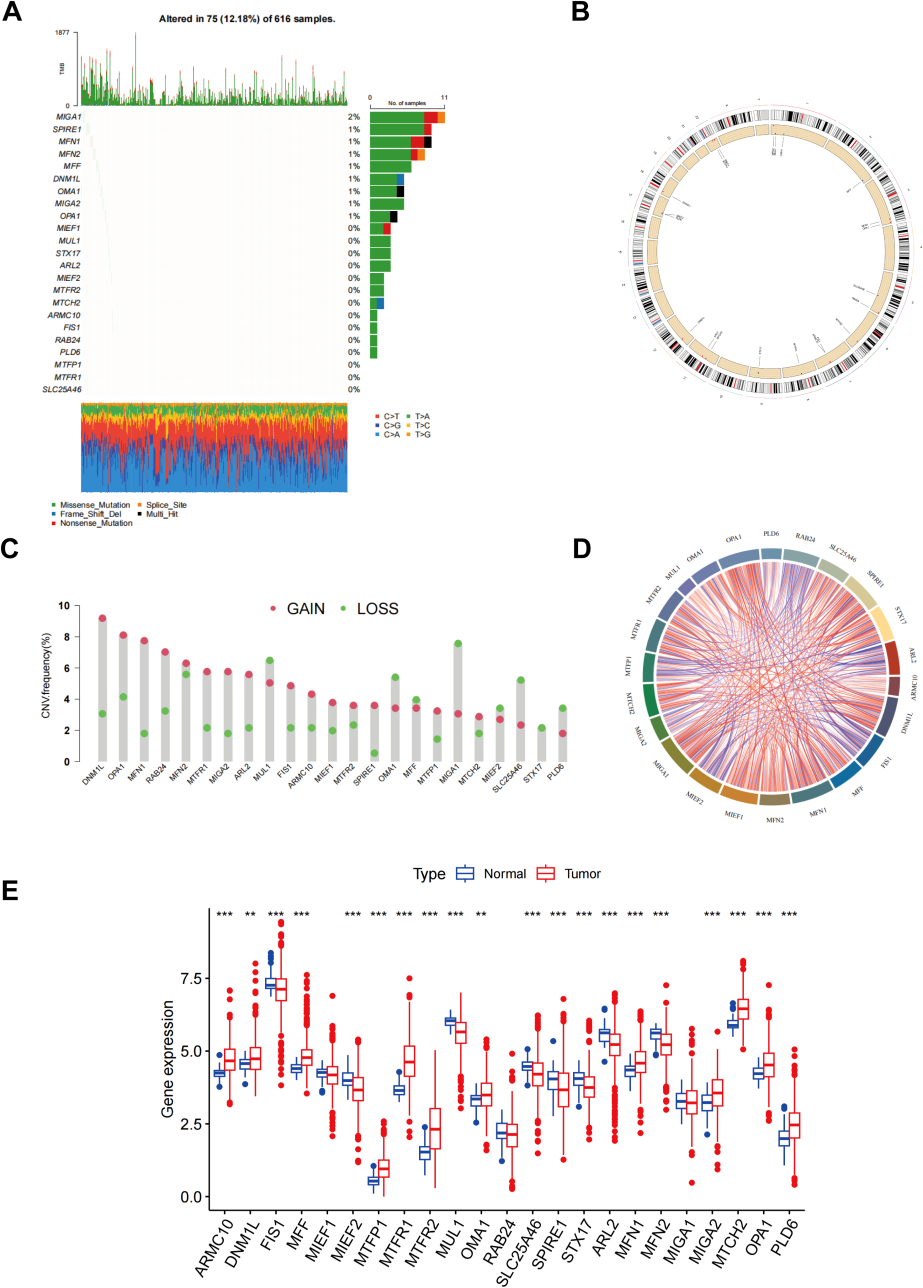
Different mutations, CNV, and expression of MD genes. **(A)** The frequency of somatic mutations in genes related to MD; **(B)** The location of CNVs of MD genes; **(C)** The CNV frequency of MD genes; **(D)** An analysis of the correlation among the MD genes; **(E)** Result of MD genes expression in both normal and LUAD tissues. p** < 0.01; ***p < 0.001.

**Figure 1A** illustrates the mutation frequency of 23 mitochondrial dynamic genes in somatic cells, revealing mutations in 75 out of 616 LUAD samples (12.18%). Among these, MIGA1 exhibited the highest mutation frequency at 2%. The locations of copy number variations (CNVs) within the mitochondrial dynamic genes were also identified (**Figure 1B**). The CNV frequency analysis indicated that DNM1L had the highest CNV frequency, nearly 9%, primarily manifested as an increase in copy number (**Figure 1C**). The chord plot depicted the interactions between mitochondrial dynamic genes, where the red chords represent positive correlations, and the blue chords indicate negative correlations (**Figure 1D**). The analysis revealed that a majority of genes linked to MD exhibited markedly elevated expression levels in tumor tissues in contrast to normal tissues. Specifically, genes such as ARMC10, DNM1L, MFF, MTFP1, MTFR1, MTFR2, OMA1, MFN1, MIGA2, MTCH2, OPA1, and PLD6 exhibited elevated expression levels in tumor tissues. Conversely, the expression levels of genes including FIS1, MIEF2, MUL1, SLC25A46, SPIRE1, STX17, ARL2, and MFN2 were markedly lower in tumor tissues than in normal tissues (**Figure 1E**). Kaplan-Meier survival curve analysis further demonstrated that elevated expression levels of OPA1, MUL1, MTFR2, MTFR1, MTPP1, MTCH2, MIGA1, MEF1, MFF, DNM1L, ARMC10, ARL2, and STX17 were significantly correlated with reduced patient survival rates, suggesting an association with poor prognosis (**Supplementary Figure S2**). In contrast, elevated expression of the MIGA2 gene was linked to improved survival outcomes, indicating a potential correlation with favorable prognosis in tumor patients (**Supplementary Figure S2**). These findings highlight the potential significance of MD-related gene expression patterns in the advancement of tumors and the prognosis of patients, underscoring their relevance as potential biomarkers or therapeutic targets in cancer research.

### Confirmation of MD subtypes

**Figure 2:**
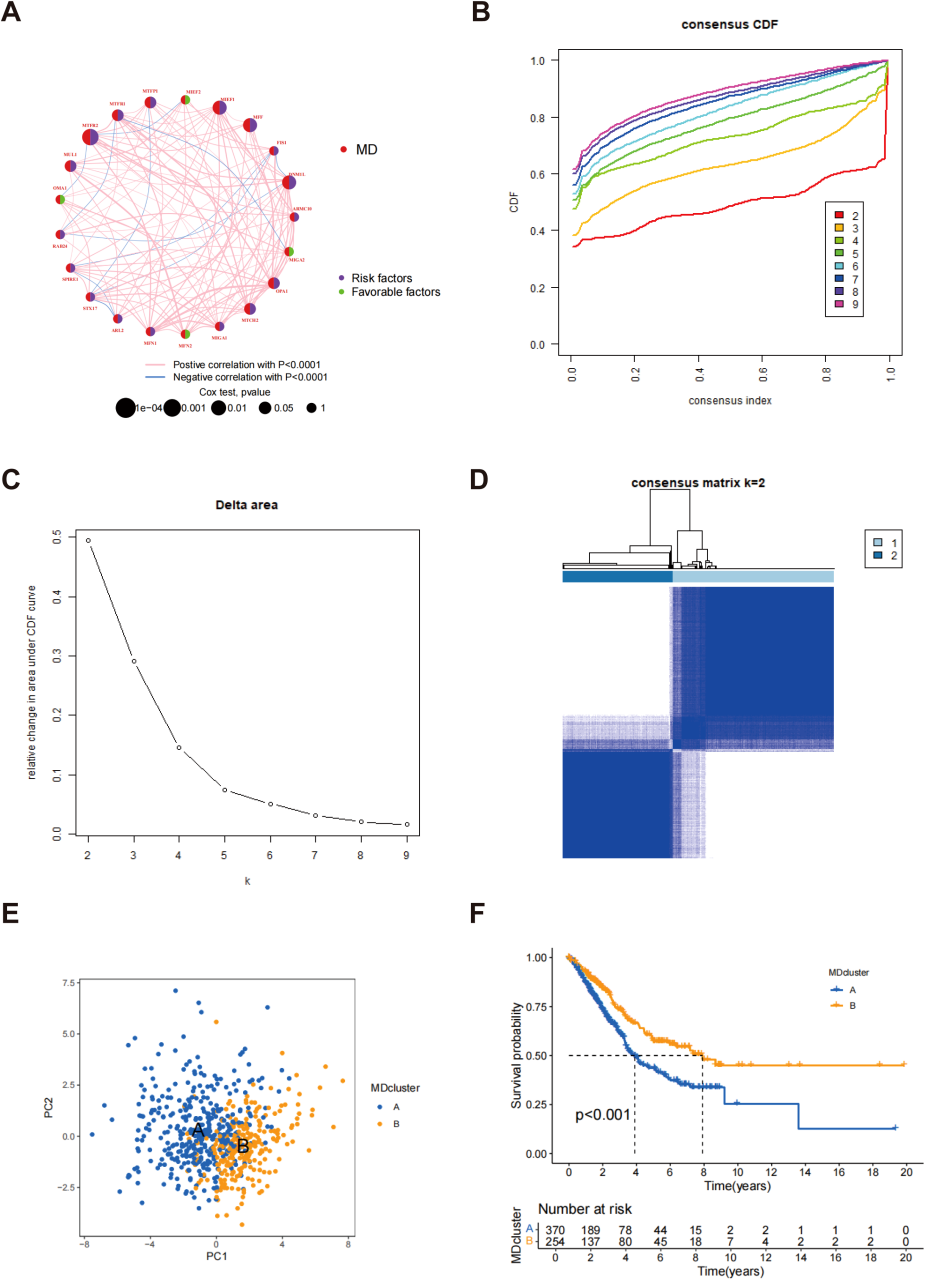
Confirmation of MD subtypes. **(A)** Network diagram of MD genes and their interactions; **(B)** Consensus CDF plot for different numbers of clusters; **(C)** Delta area representation illustrating the results of consensus clustering; **(D)** Consensus Matrix for k=2 clusters; **(E)** PCA of MD clusters; **(F)** Kaplan-Meier survival analysis for LUAD patients by MD clusters.

The interrelationships between mitochondrial kinetic genes and their significance levels in LUAD samples are illustrated in **Figure 2A**. The node sizes correspond to the p-value significance from the Cox test, with red circles representing MD genes. Genes marked with purple fans are identified as risk factors, potentially associated with poor prognosis, whereas those marked with green fans are considered favorable factors, likely offering protective effects. This analysis highlights the distinct biological roles of MD genes in LUAD patients and their potential association with clinical prognosis. A consensus clustering algorithm was applied to categorize LUAD patients according to the expression profiles of 23 MD genes (**Supplementary Figure S3**). This analysis successfully categorized the patients into two distinct subtypes: subtype A (n = 328) and subtype B (n = 305). Through multiple iterations, k = 2 was identified as the optimal choice, indicating that LUAD patients can be stratified into two distinct groups according to their MD gene expression patterns (**Figures 2B–D**). PCA further confirmed a clear separation between subtype A and subtype B, demonstrating significant differences in gene expression profiles (**Figure 2E**). The survival analysis indicated that individuals classified under subtype B demonstrated a markedly extended overall survival (OS) when contrasted with those in subtype A, with this disparity reaching statistical significance (p < 0.001) (**Figure 2F**). These findings indicate that the expression pattern of MD genes is closely associated with the prognosis of LUAD patients, with subtype B likely representing a more favorable prognosis.

### Characteristics of different MD subtypes

**Figure 3:**
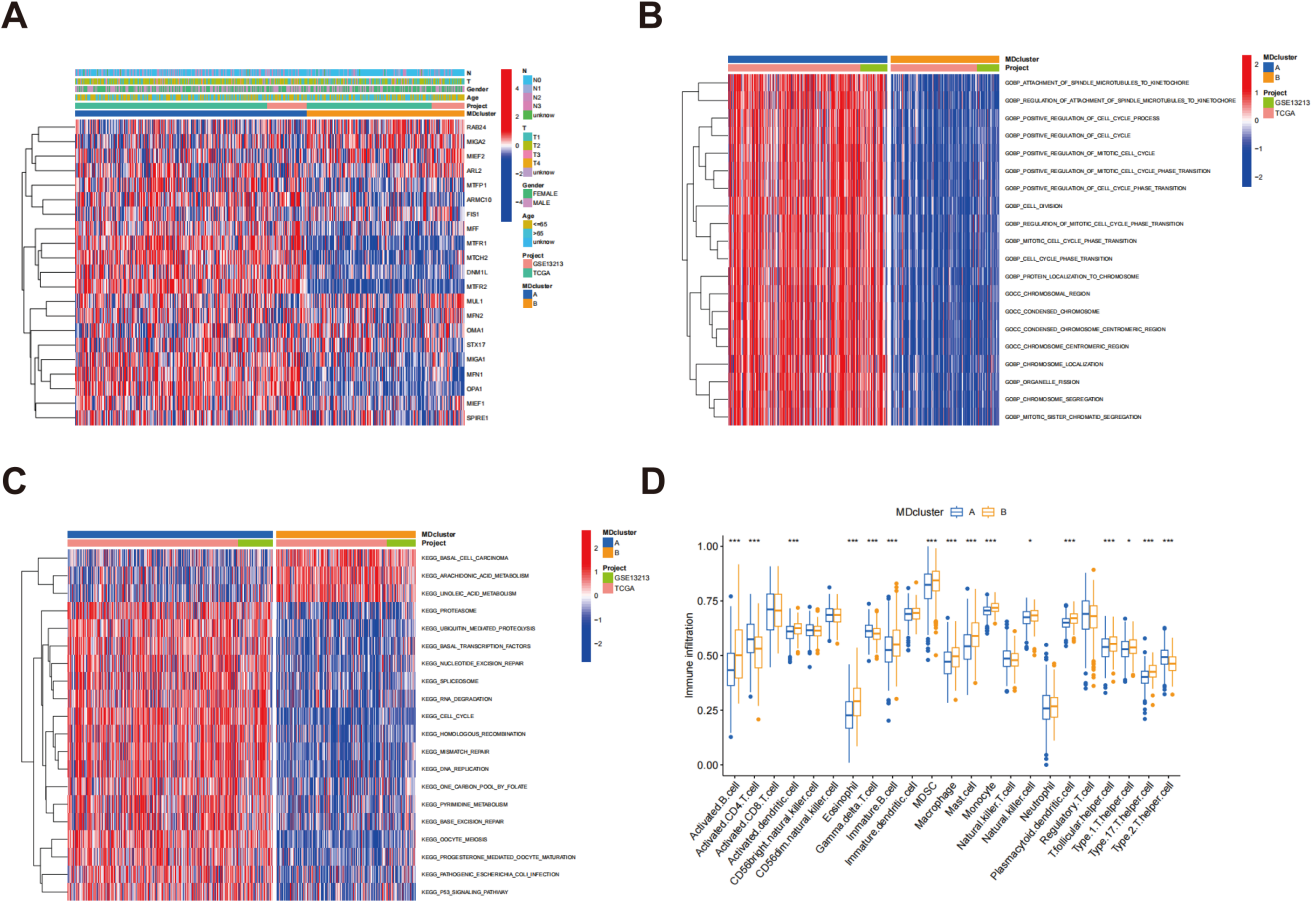
Enrichment analysis of MD subtypes and immune cell infiltration analysis. **(A)** Heatmap of mitochondrial dynamic gene expression in LUAD patients; **(B)** GSVA was conducted to assess the biological pathways across two distinct subtypes; **(C)** GSVA was utilized to evaluate the molecular functions between these two distinct subtypes; **(D)** An analysis was conducted to compare the infiltration of immune cells among the MD clusters. p* < 0.05; ***p < 0.001.

**Figure 3A** illustrates the relationship between subtypes A and B alongside the clinical characteristics of LUAD, based on data sourced from the TCGA and GSE13213 databases. GSVA analysis demonstrated a substantial enrichment of subtype B in multiple immune-related pathways, including pathogenic escherichia coli infection and the P53 signaling pathway (**Figure 3B**), indicating that subtype B may play a key role in immune responses and participate in a variety of immune-related biological processes. **Figure 3C** presents the GSVA analysis results of molecular functions for both subtypes, further supporting the active involvement of subtype B in immune responses. Among the 23 immune cell subpopulations analyzed, 16 exhibited significant differences in infiltration between subtypes A and B. Notably, the majority of immune cells were predominantly enriched in subtype B. These consisted of activated B cells, activated dendritic cells, eosinophils, immature B cells, MDSCs, mast cells, macrophages, natural killer cells, monocytes, plasmacytoid dendritic cells, follicular helper T cells, T helper type 1 cells, T helper type 17 cells, and T helper type 2 cells. These findings indicate that the immune microenvironment of subtype B is more active, with a higher presence of various immune cell populations that may significantly influence the immune response and tumor progression. In contrast, subtype A exhibited a distinct immune cell infiltration pattern, particularly in the enrichment of gamma delta T cells, activated CD4 T cells, and type 2 T helper cells. These differences suggest that the immune response in subtype A is notably different from that in subtype B (**Figure 3D**). These immune features may represent promising targets for immunotherapeutic strategies in LUAD and provide valuable insights for predicting immune responses and prognosis across subtypes.

### Gene subtypes identification

**Figure 4:**
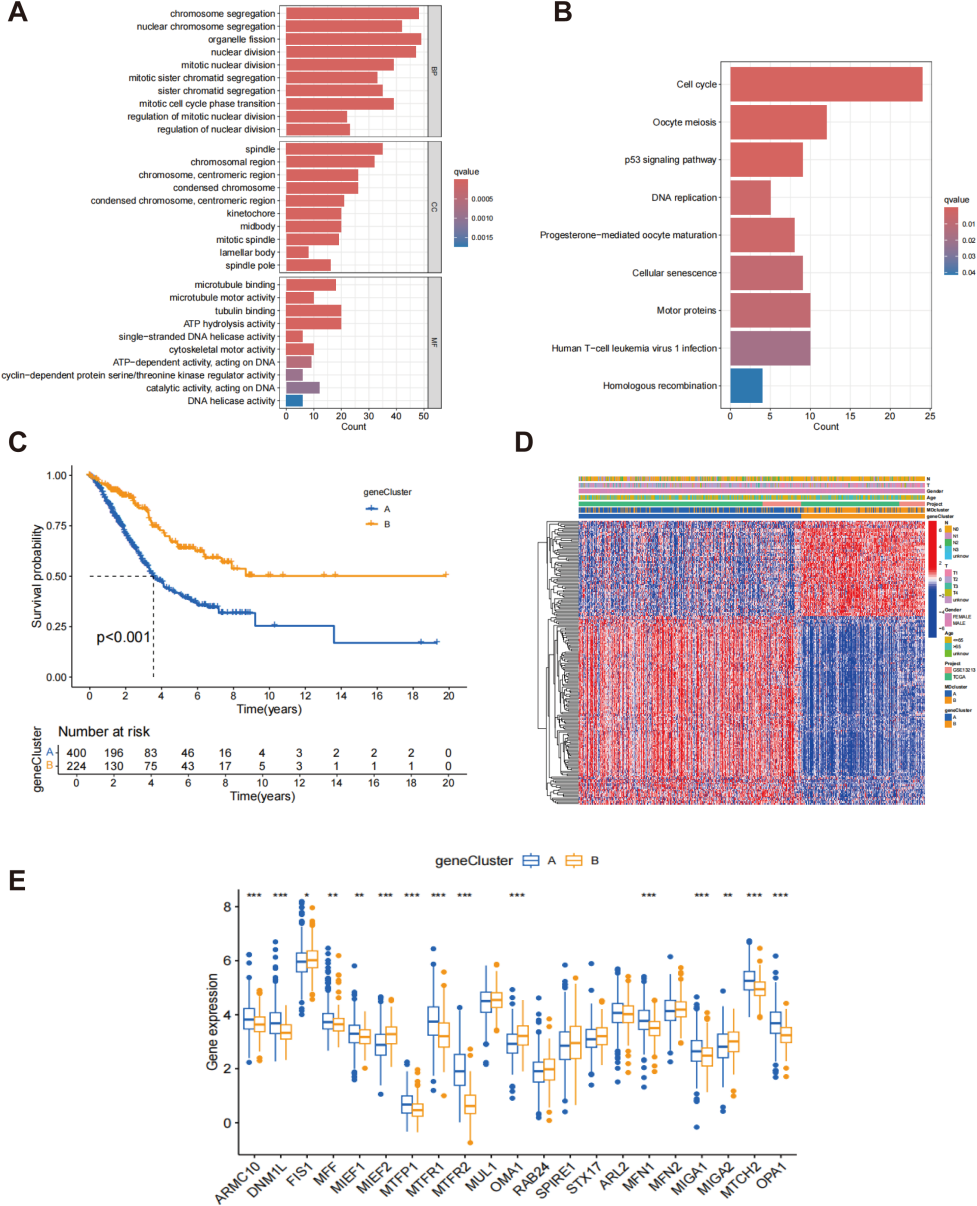
Classification of gene subtypes derived from DEGs. **(A)** Go enrichment analysis of DEGs associated with the subtypes of MD; **(B)** KEGG enrichment analysis of DEGs associated with MD subtypes; **(C)** Kaplan-Meier survival analysis of patients stratified by gene clusters A and B.; **(D)** Heatmap of two gene subtypes and associated clinical features; **(E)** Comparison of MD gene expression levels between gene clusters A and B. *p < 0.05; **p < 0.01; ***p < 0.001.

Through differential expression analysis using the R package “limma,” 217 DEGs associated with MD subtypes were identified, with significant expression differences observed across these subtypes (**Supplementary Table S2**). In order to explore the biological functions of these genes more thoroughly, GO analysis was performed. In terms of biological processes, DEGs exhibited significant enrichment in processes associated with cytokinesis and mitosis, such as chromosome segregation, organelle division, and nuclear division. Regarding cellular components, the DEGs were predominantly enriched in the spindle and chromosome regions. At the molecular function level, the DEGs were closely associated with microtubule binding, tubulin binding, and ATP hydrolytic activity (**Figure 4A**). These results indicate that the MD-associated DEGs may play a significant role in regulating cell division and the mitotic cycle. Further analysis using the KEGG pathways identified nine significantly enriched pathways, including those related to cell proliferation and division, such as the cell cycle and oocyte meiosis (**Figure 4B**). These pathways further support the hypothesis that MD subtypes are closely linked to cellular proliferation and division processes. Additionally, based on the gene expression data from MD subtypes, consensus clustering analysis was utilized to categorize patients into distinct gene subgroups. The results demonstrated that the patients were effectively grouped into two subgroups, with optimal clustering achieved (**Supplementary Figure S4**). Kaplan-Meier survival analysis indicated that patients in genome B exhibited significantly higher survival rates than those in genome A, exhibiting a statistically significant difference (p < 0.001) (**Figure 4C**). This survival disparity suggests that the gene expression pattern of MD subtypes is essential for forecasting the clinical outcomes of patients with LUAD. Finally, heatmap analysis was performed to explore the association between gene clusters and clinical features (**Figure 4D**). A comparative analysis of MD gene expression between the two gene clusters revealed that most MD genes exhibited significantly different expression levels across the clusters (**Figure 4E**). These findings provide important insights for future functional studies and the clinical application of MD genes in LUAD.

### Development and validation of the prognostic model

**Figure 5:**
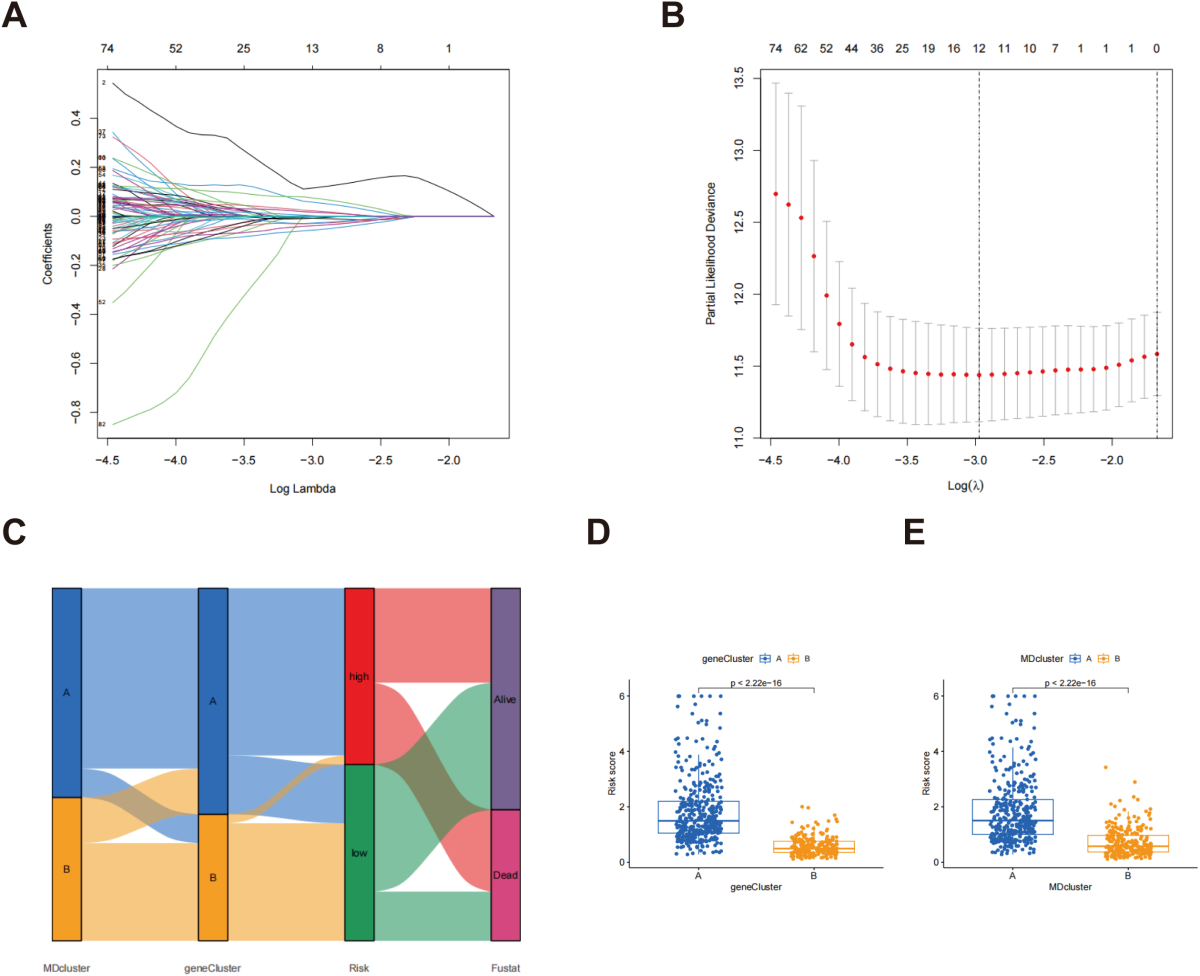
The LASSO regression and the creation of risk scores. **(A)** LASSO regression analysis for feature selection; **(B)** LASSO coefficient profiles; **(C)** Sankey diagram showing the relationships between MDcluster, genecluster, risk, and survival status; **(D)** Distribution of risk scores by genecluster; **(E)** Distribution of risk scores by MDcluster.


MD subtype-associated DEGs were utilized to establish risk scores. Through LASSO regression analysis, the number of genes was reduced to 12 (**Figures 5A, B**; **Supplementary Table S3**), and subsequent multivariate Cox regression analysis identified 5 key genes: ECT2, CHIA, CRTAC1, FAM83A, and PRAME. The calculation of the risk score was performed utilizing the subsequent formula:

Risk score = (0.257871 * ECT2) + (−0.171908077 * CHIA) + (−0.090361886 * CRTAC1) + (0.172045837 * FAM83A) + (0.082740792 * PRAME)

A Sankey diagram illustrates the relationships among MDcluster, geneCluster, risk stratification, and patient survival status, with streamlines representing the flow of patients between categories (**Figure 5C**). The results demonstrated strong consistency between MDcluster and geneCluster, indicating that individuals classified within the low-risk category experienced a greater probability of survival, whereas those identified in the high-risk group exhibited increased mortality (**Figure 5C**). Additionally, **Figures 5D, E** show that patients in group A exhibited markedly elevated risk scores within both geneCluster and MDcluster.

**Figure 6:**
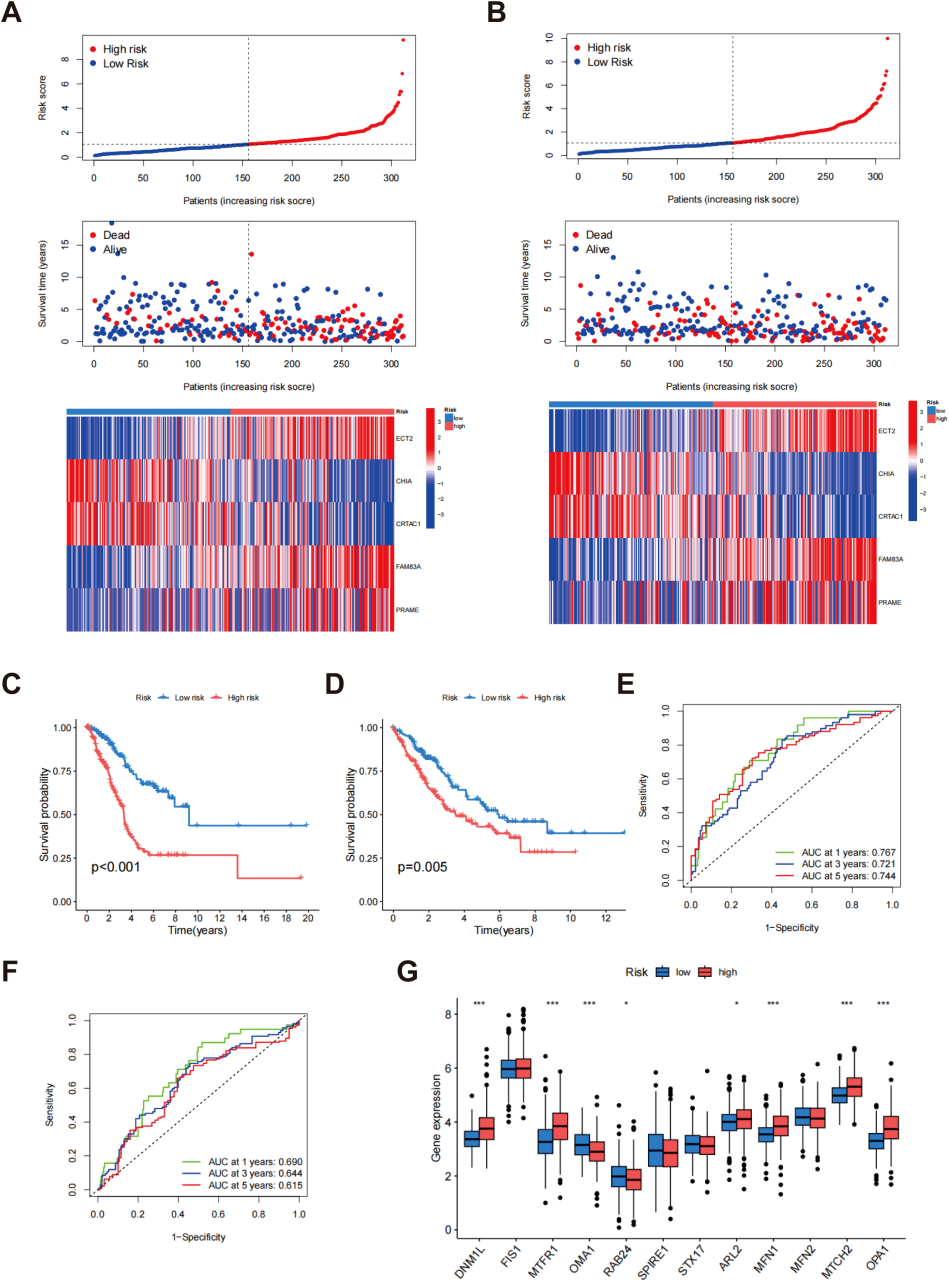
Evaluation of risk scores. The allocation of risk scores, survival outcomes, and prognostic gene expression levels within the training cohort **(A)** and testing cohort **(B)**; Kaplan-Meier survival analysis for the training cohort **(C)** and testing cohort **(D)**; ROC curves for the training cohort **(E)** and testing cohort **(F, G)** Assessment of MD gene expression levels contrasting low-risk and high-risk groups. p* < 0.05; ***p < 0.001.

Patients were allocated randomly into two groups: the training group and the testing group, with each comprising 312 participants. In the training group, the gene expression heatmap indicated that high-risk patients classified as high-risk displayed significantly increased expression of the ECT2, FAM83A, and PRAME genes, while the CHIA and CRTAC1 genes showed lower expression levels (**Figure 6A**). As the risk score increased from left to right, the proportion of patients who died also increased, indicating that elevated risk scores correlated with reduced survival durations. This trend further validated the risk score as an effective prognostic indicator (**Figure 6A**). Similar results were observed and confirmed in the test group (**Figure 6B**). Survival analysis in the training group demonstrated that the risk score served as a substantial predictor of patient outcomes, with low-risk patients showing a substantially higher survival rate than high-risk patients (p < 0.001) (**Figure 6C**). This finding was similarly confirmed in the testing group (p = 0.005) (**Figure 6D**), indicating that the risk score is effective in predicting survival across different patient cohorts and holds strong clinical relevance. Additionally, the area under the curve (AUC) at 1, 3, and 5 years for the training and test groups were 0.767, 0.721, and 0.744 for the training group (**Figure 6E**), and 0.690, 0.644, and 0.615 for the test group (**Figure 6F**), respectively. Despite the relatively lower AUC values in the test group, it still demonstrated reliable prognostic predictive capability, further supporting the accuracy and robustness of the risk score at various time intervals. **Figure 6G** depicts the differential expression of MD genes among different risk score categories. A notable elevation in the expression levels of the majority of MD genes was observed in the high-risk cohort compared to the low-risk cohort. This observation implies that these genes may be crucial in the risk assessment and prognostic evaluation of patients with LUAD. These results offer significant support for the clinical implementation of a risk scoring system based on MD gene expression to enhance prognostic accuracy for LUAD patients.

### Construction of a nomogram

**Figure 7:**
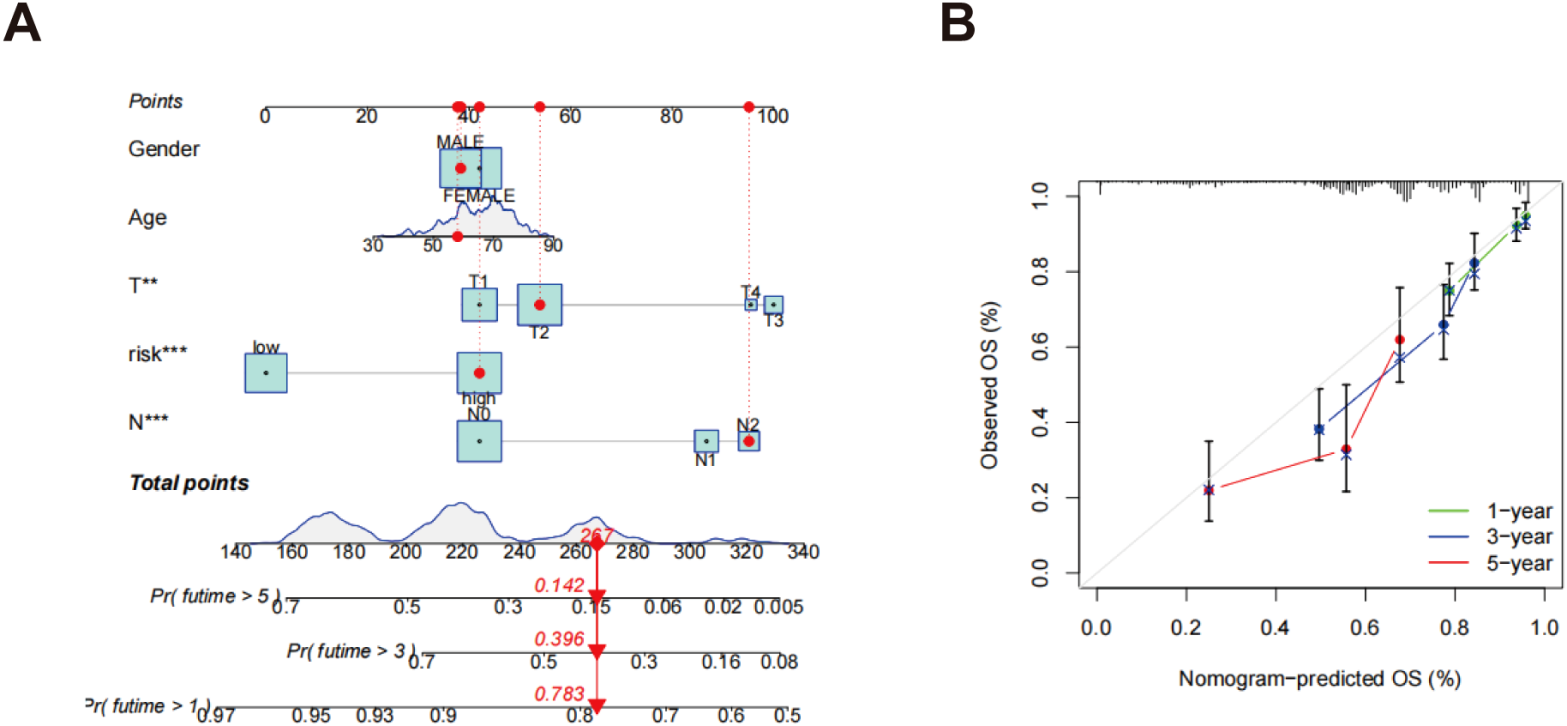
Development of a nomogram. **(A)** A nomogram designed to forecast OS at the 1, 3, and 5-year marks; **(B)** Calibration curves associated with the nomogram that estimate OS. **p < 0.01; ***p < 0.001.

The nomogram incorporated multiple variables, including age, gender, N-stage, T-stage, and risk score, into a comprehensive model for predicting individual patients’ survival probabilities at 1, 3, and 5 years (**Figure 7A**). The calibration curves further validated the model’s predictive accuracy, illustrating a robust correlation between the survival probabilities estimated by the nomogram and the outcomes that were actually observed (**Figure 7B**). The green, blue, and red curves represent the survival predictions at 1, 3, and 5 years, respectively, emphasizing the efficacy of the model across various temporal milestones. The near-diagonal alignment of the calibration curves indicates that the nomogram accurately reflects patients’ actual survival and demonstrates excellent predictive power and clinical applicability. These findings suggest that the nomogram provides reliable, individualized survival predictions, offering significant potential for improving patient prognosis and clinical decision-making.

### Evaluation of TME

**Figure 8:**
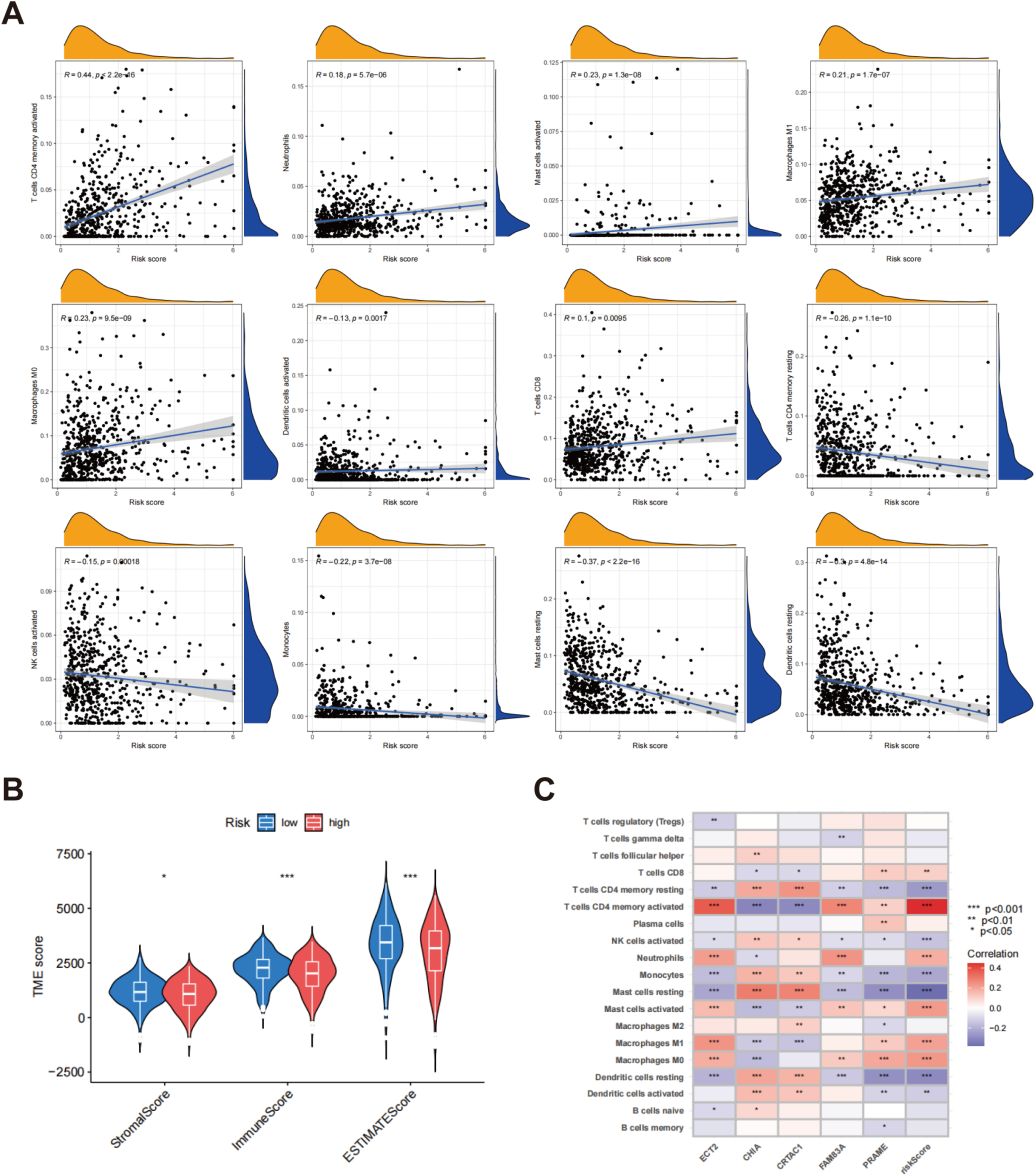
Assessment of TME in different risk scors. **(A)** Connections between immune cell types and risk scores; **(B)** A comparative assessment of TME scores was conducted between the groups identified as low risk and high risk; **(C)** Correlation heatmap between gene expression, risk score, and immune cell infiltration. *p < 0.05; p** < 0.01; ***p < 0.001.

Scatter plots revealed a noteworthy positive association between the risk score and various immune cell populations, particularly with the infiltration of T cells CD4 memory activated, neutrophils, mast cells activated, macrophages M0, dendritic cells activated, macrophages M1, and T cells CD8. Conversely, the risk score exhibited an inverse relationship with the infiltration levels of NK cells activated, T cells CD4 memory resting, monocytes, mast cells resting, and dendritic cells resting (**Figure 8A**). Furthermore, an analysis of TME scores across various risk categories showed that stromal and immune scores were generally higher in low-risk patients, while these scores were significantly lower in high-risk patients (**Figure 8B**). In order to gain deeper insights into the association between gene expression and the presence of immune cells, heatmaps were generated to illustrate the correlation between genes associated with risk scores (ECT2, CHIA, CRTAC1, FAM83A, and PRAME) and various immune cell types (**Figure 8C**). The heatmap revealed significant correlations between these five genes and most immune cell populations, suggesting that these genes might significantly influence the formation of the tumor immune microenvironment.

### Analysis of TMB, tumor stemness score, somatic mutation features and drug sensitivity

**Figure 9:**
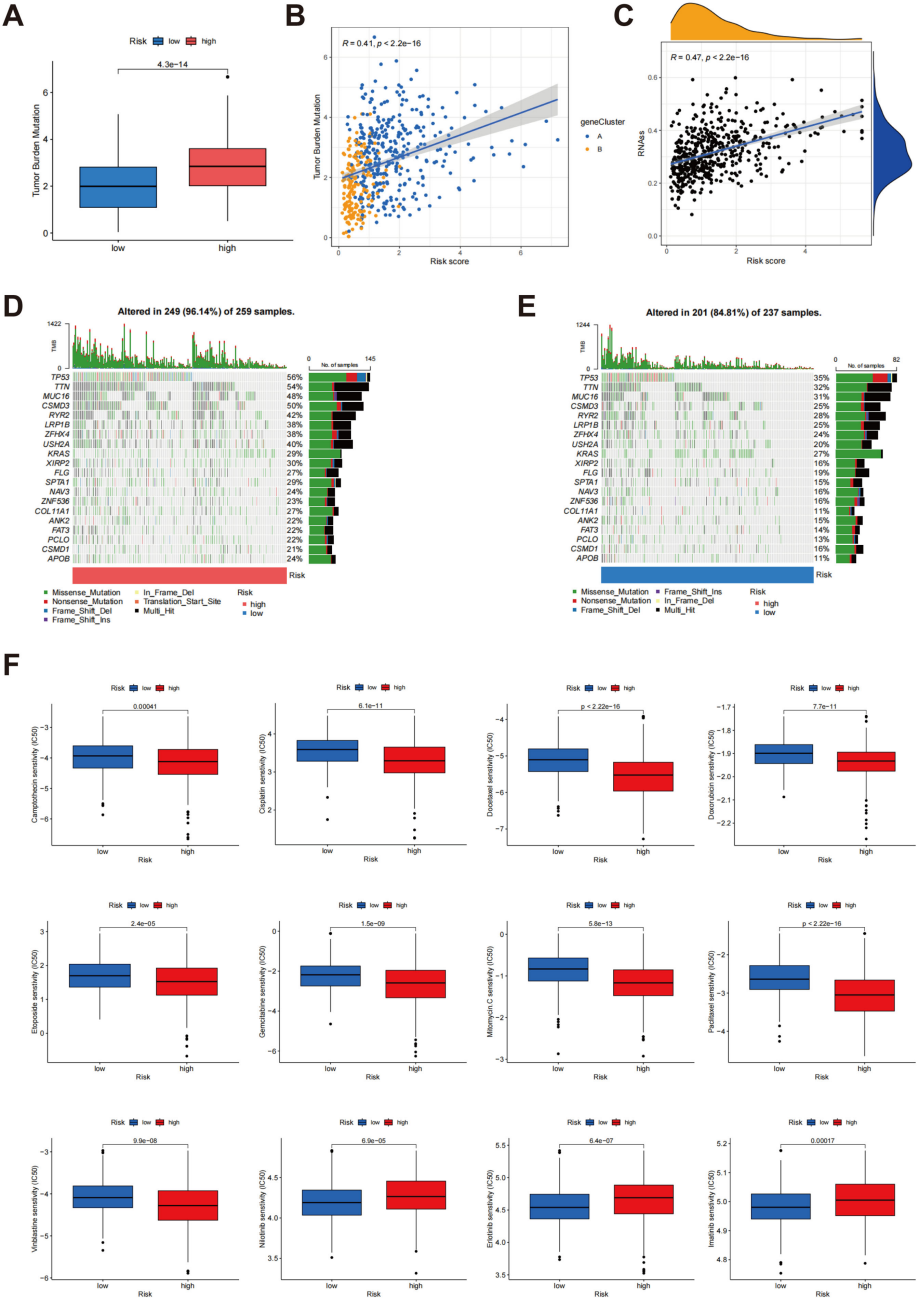
Analysis of TMB, tumour stemness score and somatic mutation characteristics in different risk scores. **(A)** Comparison of TMB between groups classified as low risk and high risk; **(B)** Association between risk score and TMB; **(C)** Correlation between risk score and tumor stemness score; **(D, E)** Comparison of somatic mutation profiles between high-risk and low-risk cohorts; **(F)** Relationship between risk scores and drug sensitivity.

Box plots demonstrated a noteworthy distinction in TMB between the low-risk and high-risk cohorts, with the high-risk group exhibiting a substantially higher TMB (p < 4.3e-14) (**Figure 9A**). Additionally, the scatter plot demonstrated a positive correlation between risk score and TMB (r = 0.41, p < 2.2e-16) (**Figure 9B**). These findings indicate that higher risk scores are associated with increased mutation burden, potentially reflecting more aggressive tumor characteristics. Further analysis revealed a significant positive association between the risk score and tumor stemness score (r = 0.47, p < 2.2e-16) (**Figure 9C**), suggesting that individuals with elevated risk scores tend to exhibit higher tumor stemness, a factor closely linked to tumor progression and resistance to treatment. The findings collectively highlight the promise of risk scores as biomarkers for assessing tumor biology, including mutation burden and stemness, which are critical for comprehending tumor aggressiveness and therapeutic challenges.

The tumor mutation profiles were compared between the high-risk and low-risk cohorts (**Figures 9D, E**). In the cohort identified as high-risk, 96.14% (249/259) of the samples exhibited mutations, with TP53 (56%), TTN (54%), and MUC16 (48%) being the most commonly mutated genes. The TMB was also significantly higher in this group. Notably, the elevated frequency of TP53 mutations suggests a more unstable genome in high-risk patients, potentially contributing to more aggressive tumor characteristics. In contrast, 84.81% (201/237) of the samples in the low-risk cohort showed mutations, with TP53 (35%), TTN (32%), and MUC16 (31%) being the most commonly mutated genes. However, both the mutation frequency and overall TMB were significantly reduced in the low-risk cohort when juxtaposed with the high-risk cohort. This lower mutation load and reduced mutation frequency indicate a relatively stable genome, which correlates with an improved clinical outcome for these individuals. These results indicate that the tumor genomes of high-risk patients exhibit greater instability, with a higher mutational load and frequent alterations in key tumor suppressor genes, such as TP53. These genetic alterations are likely to drive tumor progression and negatively impact patient outcomes. In contrast, the low-risk group demonstrates a more stable genomic landscape, which correlates with improved prognosis. Collectively, these analyses underscore the connection between risk scores and genomic mutational load, offering deeper insights into how risk stratification is linked to underlying tumor biology.

Further analysis was conducted to investigate the correlation between risk scores and drug sensitivity, as illustrated in **Figure 9F**. The findings indicated that individuals classified within the high-risk cohort exhibited heightened sensitivity to a range of chemotherapeutic agents, evidenced by reduced IC50 values for camptothecin, cisplatin, doxorubicin, docetaxel, etoposide, mitomycin C, paclitaxel, gemcitabine, and vinblastine. This suggests that high-risk patients may experience better efficacy with these drugs. Conversely, the low-risk group demonstrated lower IC50 values for erlotinib, nilotinib, and imatinib, indicating higher sensitivity to these specific agents, which may make them better candidates for treatment with these drugs. These findings provide personalized treatment strategies based on risk score and contribute to understanding the connection between drug sensitivity and tumor biology, thus offering valuable insights for clinical decision-making.

### Expression and prognostic implications of MTCH2 in LUAD

**Figure 10:**
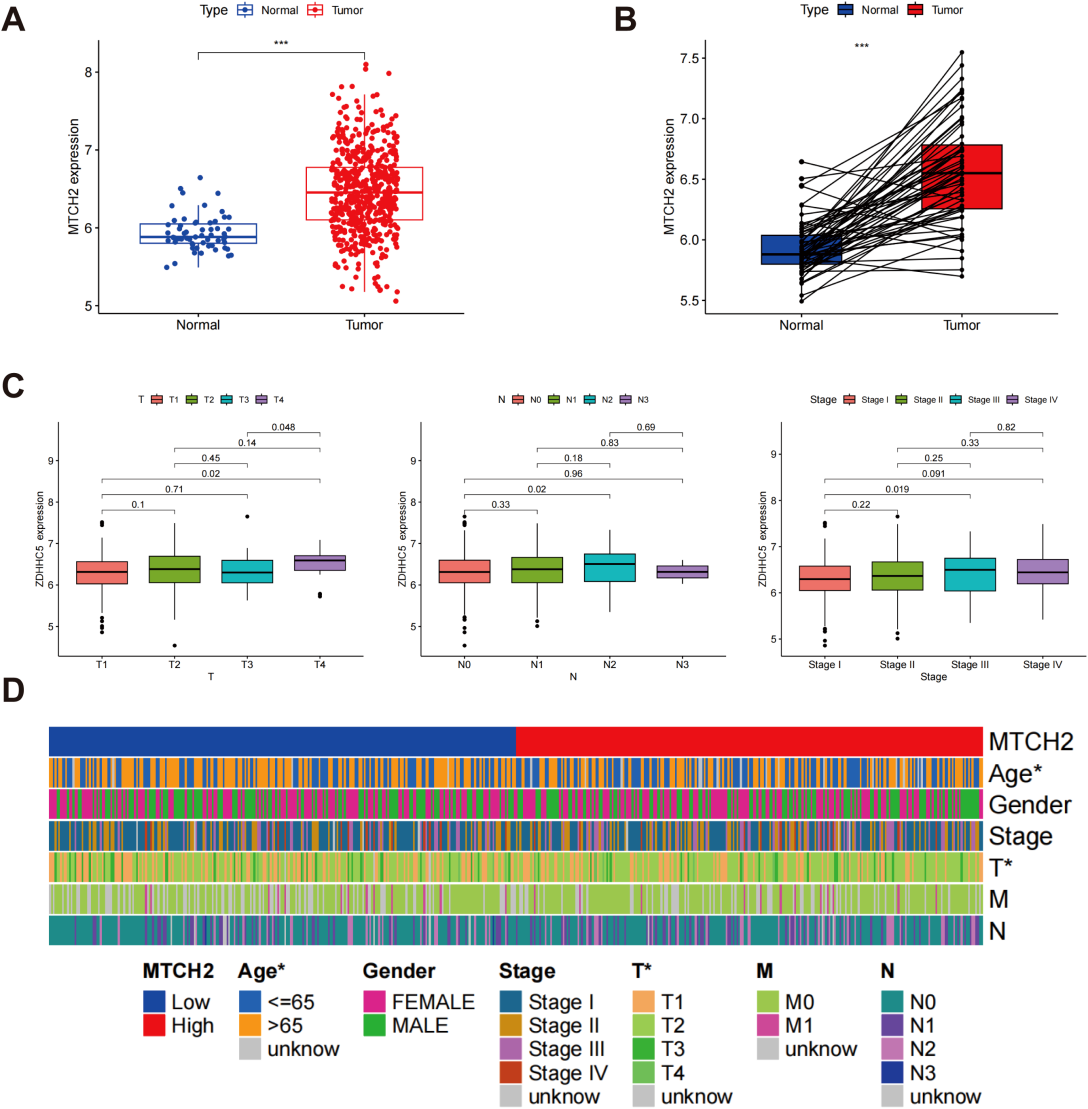
MTCH2 expression and clinical characterization in LUAD. **(A)** Comparison of MTCH2 expression in normal versus tumor tissues; **(B)** Comparative analysis of MTCH2 expression in matched normal and tumor tissue samples; **(C)** Association between MTCH2 expression levels and N stage, T stage and clinical stage; **(D)** Heatmap illustrating the distribution of MTCH2 expression levels and clinical characteristics, including gender, age, stage, T, M, and N classifications. *p < 0.05; ***p < 0.001.

The analyses presented indicate a significant correlation between increased MTCH2 expression and unfavorable prognosis. Nevertheless, as of now, there has been no investigation that has specifically examined the function of MTCH2 in LUAD. Given the high incidence of LUAD worldwide and its clinical relevance, this research aimed to explore the possible involvement of MTCH2 in LUAD. Further analysis showed that the expression of MTCH2 was significantly elevated in LUAD specimens (n = 541) compared to normal samples (n = 59) (**Figure 10A**). The results of pairwise analysis also confirmed this finding, indicating a significant disparity in MTCH2 expression between tumor and normal tissues from the same patient (**Figure 10B**). Additionally, the influence of various clinicopathological characteristics on MTCH2 expression in LUAD patients was examined. **Figure 10C** shows that MTCH2 expression is significantly elevated in T4, N2, and Stage III compared to T1, N0, and Stage I, respectively. **Figure 10D** further reveals an association between high MTCH2 expression and advanced T stage. Overall, these findings suggest that MTCH2 expression levels are closely linked to N stage, T stage, and clinical stage.

**Figure 11:**
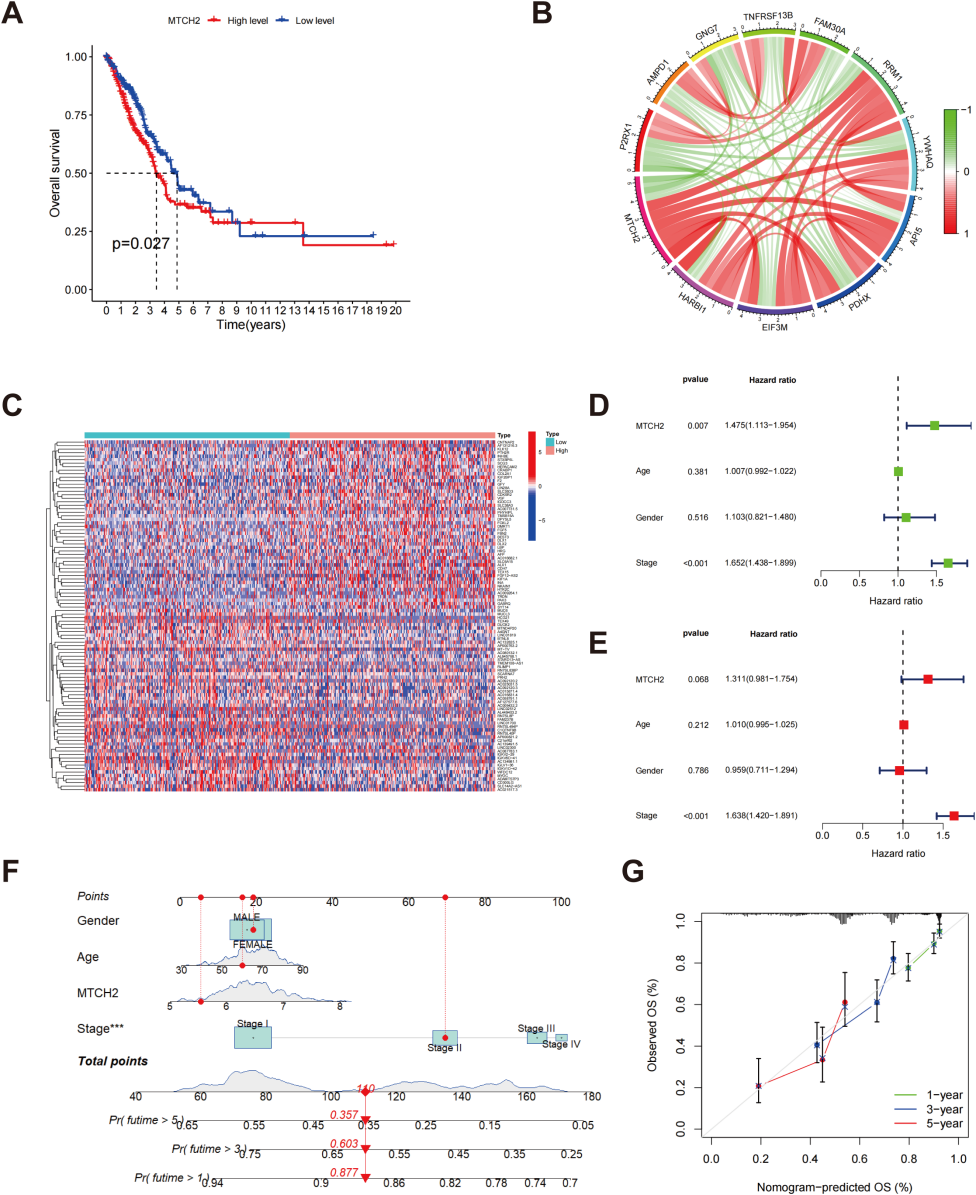
Relationship between MTCH2 and prognosis. **(A)** The Kaplan-Meier survival analysis displays OS rates among patients categorized by high versus low MTCH2 expression levels; **(B)** Circos plot showing MTCH2 co-expression with the six most positively and five most negatively correlated genes; **(C)** A heatmap represents the top 50 DEGs identified in groups with high and low MTCH2 expression; **(D)** Univariate Cox regression forest plot for MTCH2 expression, age, gender, and stage; **(E)** Multivariate Cox regression forest plot for MTCH2 expression, gender, stage, and age; **(F)** Nomogram predicting 1-, 3-, and 5-year survival probabilities; **(G)** A calibration plot is included, which assesses the nomogram’s predictive accuracy for survival probabilities at the 1-, 3-, and 5-year marks. ***p < 0.001.

The results of Kaplan-Meier survival analysis indicated that individuals exhibiting low expression levels of MTCH2 experienced significantly prolonged survival durations in comparison to those with elevated MTCH2 expression (p = 0.027) (**Figure 11A**). These findings imply that the expression level of MTCH2 may have a significant correlation with the prognostic survival outcomes of patients, with high expression potentially indicating a poorer prognosis. To further explore the potential involvement of MTCH2 in oncogenesis, a co-expression analysis was conducted to pinpoint genes that exhibit a strong correlation with MTCH2 expression. The analysis indicated that the six genes with the highest correlation coefficients were RRM1, YWHAQ, API5, PDHX, EIF3M, and HARB1, while the five genes with the lowest correlation coefficients were P2RX1, AMPD1, GNG7, TNFRSF13B, and FAM30A (**Figure 11B**; **Supplementary Figure S5**). The co-expression of these genes may provide insight into the molecular pathways through which MTCH2 may contribute to tumorigenesis. Additionally, 918 DEGs were identified between the high and low MTCH2 expression groups (**Supplementary Table S4**, FDR < 0.05, |Log2 FC| > 1, p < 0.05). Of these, 413 genes were upregulated in the high-expression cohort, while 505 genes were upregulated in the low-expression cohort, indicating the significant impact of MTCH2 expression on tumor gene expression profiles. A heatmap was generated to display the top 50 DEGs in each group (**Figure 11C**), providing potential targets for further investigation of MTCH2 function. Cox regression analysis confirmed that both MTCH2 expression level and clinical stage were independent prognostic indicators (**Figures 11D, E**). In order to further substantiate the prognostic significance of MTCH2, a nomogram was established combining gender, age, MTCH2 expression, and clinical stage to predict the 1-, 3-, and 5-year survival probabilities (**Figure 11F**). The accuracy of the model was confirmed through calibration plots, demonstrating good predictive performance (**Figure 11G**).

### Enrichment analysis of MTCH2 DEGs, association of MTCH2 with immune infiltration and drug sensitivity

**Figure 12:**
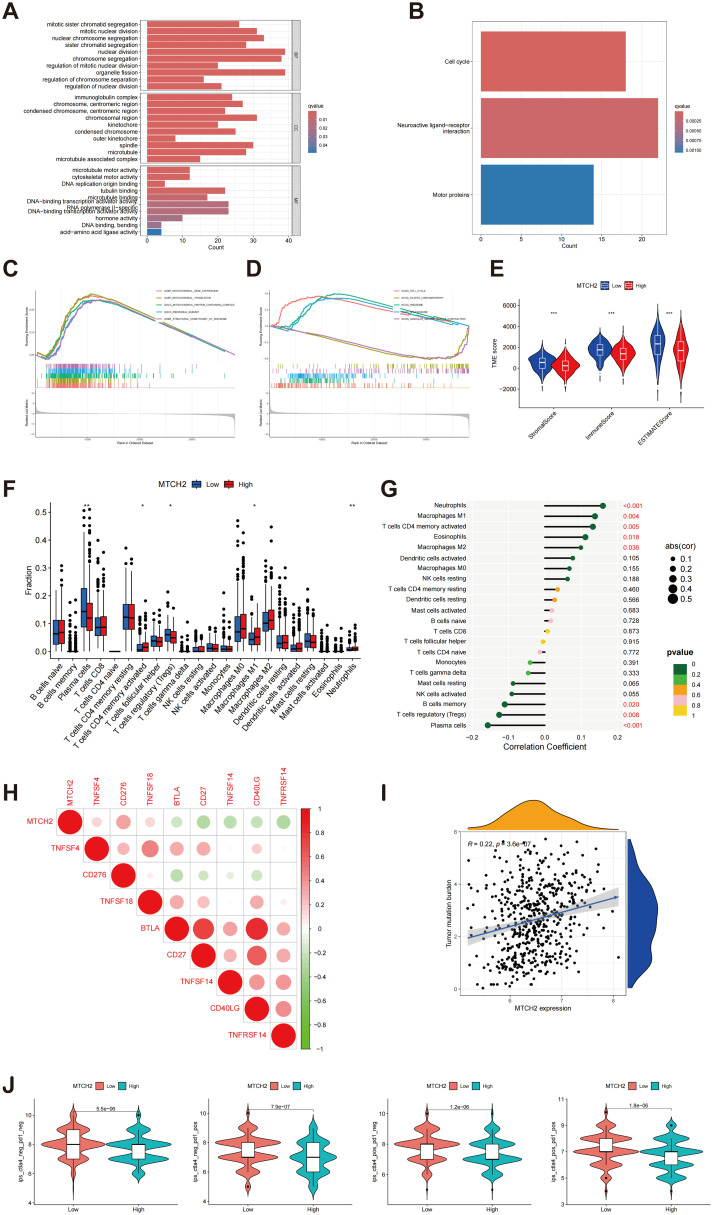
Enrichment analysis of MTCH2 DEGs and correlation of MTCH2 expression with immune infiltration. **(A)** GO enrichment analysis was conducted on DEGs; **(B)** Analysis of the KEGG pathways was performed on DEGs; **(C–D)** GSEA analysis of MTCH2 DEGs; **(E)** Comparison of stromal, immune, and ESTIMATE scores between high and low MTCH2 expression groups; **(F)** Distribution of 22 immune cell types between high and low MTCH2 expression groups; **(G)** Connection analysis between MTCH2 expression and various immune cell types; **(H)** Heatmap showing connection between MTCH2 expression and immune checkpoint genes; **(I)** Association between MTCH2 expression and TMB; **(J)** Connection between MTCH2 expression and immunotherapy. *p < 0.05; p** < 0.01; ***p < 0.001.

An enrichment analysis of DEGs was conducted to compare groups exhibiting high and low levels of MTCH2 expression. The results from the GO analysis indicated that these DEGs were mainly linked to biological processes, including organelle fission, nuclear division, and chromosome segregation. At the cellular component level, these genes were principally enriched in chromosome regions and spindle bodies, while molecular function analysis showed enrichment in tubulin binding, and DNA-binding transcription activator activity (**Figure 12A**). KEGG pathway analysis revealed significant pathways, including neuroactive ligand-receptor interactions, cell cycle regulation, and motor protein activity (**Figure 12B**). GSEA further demonstrated that the group with high expression of MTCH2 exhibited notable enrichment in the cell cycle pathway (**Figures 12C, D**). Subsequently, the TME was examined, demonstrating notably elevated immune, stromal, and estimate scores in the group exhibiting low expression of MTCH2 when contrasted with the group showing high expression (**Figure 12E**). Among 22 immune cell types, four showed marked differences (**Figure 12F**). Correlation analysis indicated associations between MTCH2 expression and various immune cell types: neutrophils (r = 0.16, p = 7e-04), macrophages M2 (r = 0.099, p = 0.036), activated CD4 memory T cells (r = 0.13, p = 0.0051), macrophages M1 (r = 0.14, p = 0.0035), and eosinophils (r = 0.11, p = 0.018) were positively correlated; whereas plasma cells (r = -0.16, p = 0.00086), regulatory T cells (Tregs) (r = -0.13, p = 0.0078), and memory B cells (r = -0.11, p = 0.02) showed negative correlations (**Figure 12G** and **Supplementary Figure S6**). Additionally, eight immune checkpoint (ICP) genes were found to be significantly correlated with MTCH2 (p < 0.001), with CD276 demonstrating the strongest correlation (cor = 0.34) (**Figure 12H**). Analysis of TMB revealed a positive association with MTCH2 expression (r = 0.22, p < 0.05), where elevated MTCH2 expression was linked to increased TMB (**Figure 12I**). Finally, immunotherapy efficacy was analyzed, showing that patients with low MTCH2 expression achieved better responses to programmed cell death protein 1 (PD-1) inhibitors, cytotoxic T-lymphocyte-associated protein 4 (CTLA-4) inhibitors, as well as to a combination of both PD-1 and CTLA-4 therapies (**Figure 12J**).

The correlation between the expression levels of MTCH2 and the sensitivity to pharmacological agents was investigated in greater detail. The analysis indicated that individuals displaying elevated MTCH2 expression levels demonstrated reduced IC50 values for drugs such as 5-Fluorouracil, cediranib, cytarabine, telomerase inhibitor IX, gallibiscoquinazole, savolitinib, and talazoparib, suggesting increased sensitivity to these agents. Conversely, higher IC50 values were observed for ribociclib, doramapimod, selumetinib, mitoxantrone, and entinostat, indicating reduced sensitivity. These findings suggest a correlation between MTCH2 expression levels and drug sensitivity (**Supplementary Figure S7**).

### Functional experimental validation

**Figure 13:**
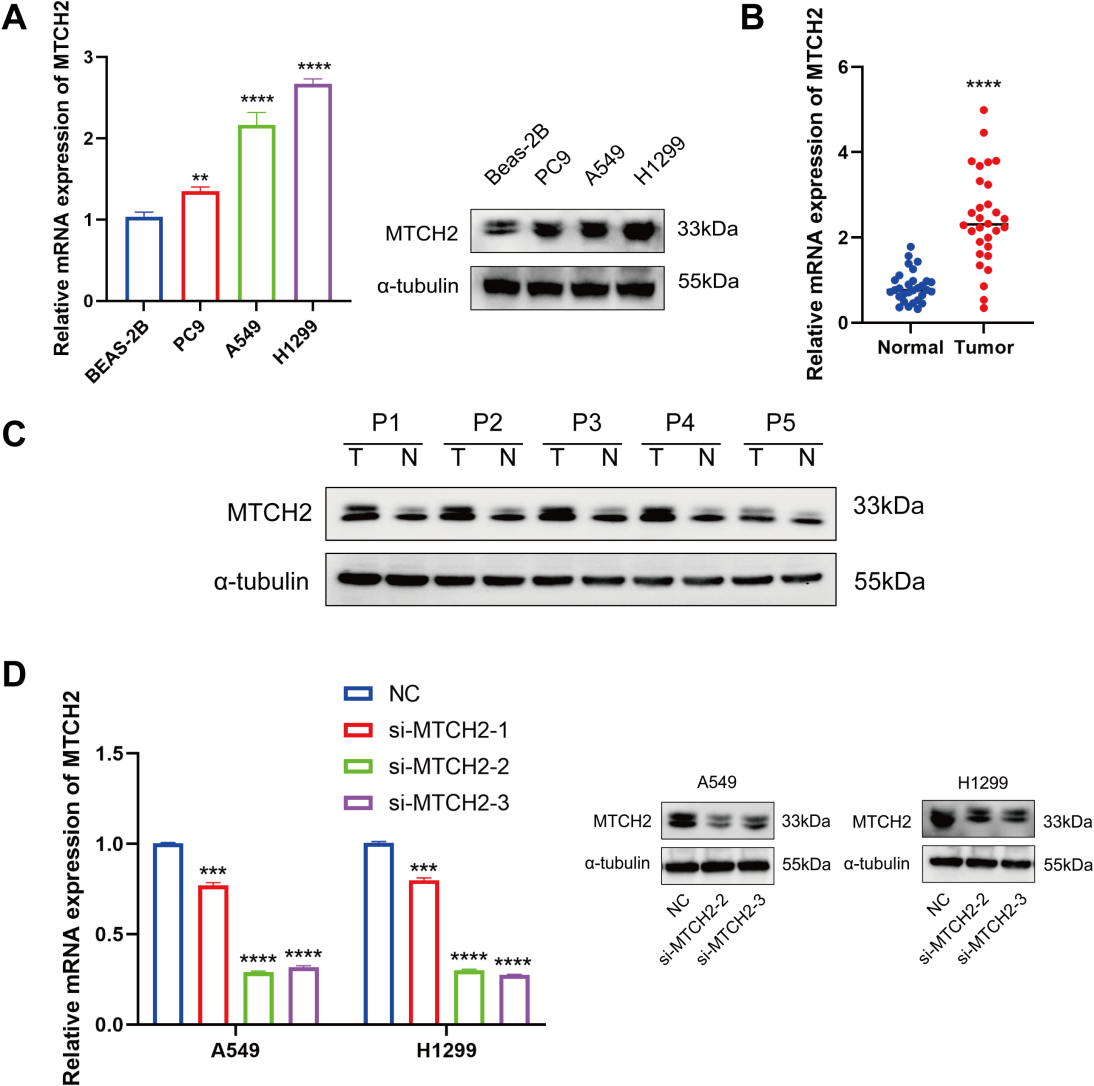
MTCH2 expression levels in normal and tumor cells and tissues, and the effect of siRNA knockdown of MTCH2. **(A)** Relative mRNA and protein expression levels of MTCH2 in normal lung cell line BEAS-2B compared to lung cancer cell lines PC9, A549, and H1299; **(B)** Comparison of MTCH2 mRNA expression in 30 paired normal and tumor LUAD tissues; **(C)** The protein expression of MTCH2 in matched normal (N) and tumor (T) samples obtained from five LUAD patients; **(D)** MTCH2 knockdown reduces mRNA and protein levels in A549 and H1299 cells. p** < 0.01; ***p < 0.001; ****p < 0.0001.

To further investigate the role of MTCH2 in LUAD, several experiments were conducted. First, the comparative mRNA expression levels of MTCH2 were examined in the normal lung cell line BEAS-2B alongside the lung cancer cell lines PC9, A549, and H1299. The findings revealed a notable increase in MTCH2 expression within the cancer cell lines (PC9, A549, and H1299) in contrast to the normal cell line (**Figure 13A**). This observation was additionally validated through Western blot analysis, revealing a notable elevation in MTCH2 protein expression within the cancer cell lines (**Figure 13A**). Additionally, MTCH2 mRNA expression was compared between normal and tumor tissue samples from 30 pairs of LUAD patients, revealing a significant upregulation of MTCH2 expression in the tumor tissues (**Figure 13B**). In a subsequent experiment, paired normal (N) and tumor (T) samples from five LUAD patients were analyzed. The findings corroborated that the expression levels of the MTCH2 protein were markedly increased in the tumor tissues (**Figure 13C**). To further assess the function of MTCH2 in LUAD, three siRNAs (si-MTCH2-1, si-MTCH2-2, and si-MTCH2-3) were used to reduce MTCH2 expression in the H1299 and A549 lung cancer cell lines. The expression levels of MTCH2 mRNA were markedly diminished in all groups treated with siRNA when compared to the negative control (NC) group, with si-MTCH2–2 and si-MTCH2–3 exhibiting the most effective knockdown (**Figure 13D**). Consequently, si-MTCH2–2 and si-MTCH2–3 were selected for further experimentation. Western blot analysis verified that MTCH2 protein levels were notably reduced in A549 and H1299 cells following siRNA treatment (**Figure 13D**). These observations imply that the expression of MTCH2 is heightened in LUAD and could be instrumental in the progression of tumorigenesis.

**Figure 14:**
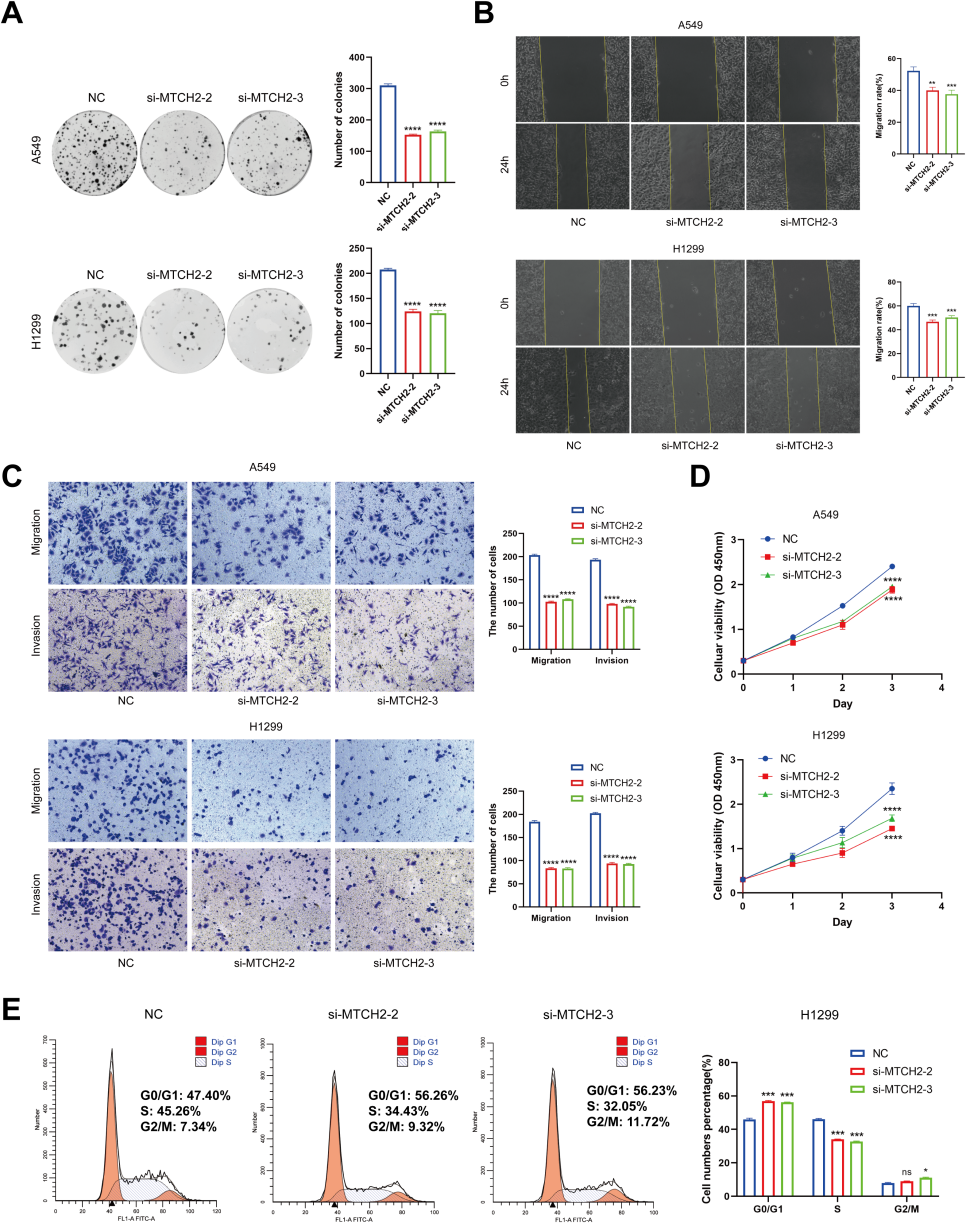
The Impact of MTCH2 knockdown on proliferation, invasion, migration, and cell cycle of LUAD cells. **(A)** Colony formation assay in H1299 and A549 cells after MTCH2 knockdown; **(B)** Wound healing assay in H1299 and A549 cells after MTCH2 knockdown. Scale bars, 200 µm; **(C)** Transwell migration and invasion assays in H1299 and A549 cells after MTCH2 knockdown. Scale bars, 200 µm; **(D)** CCK-8 assay in H1299 and A549 cells after MTCH2 knockdown; **(E)** Cell cycle analysis of H1299 cells following MTCH2 knockdown. *p < 0.05; p** < 0.01; ***p < 0.001; ****p < 0.0001.

The impact of MTCH2 knockdown on LUAD cell proliferation, invasion, and cell cycle progression were further evaluated. The results from the clone formation assay and CCK-8 analysis revealed that the reduction of MTCH2 expression notably suppressed the growth of A549 and H1299 cell lines (**Figures 14A, D**), indicating that MTCH2 might contribute positively to the proliferation of LUAD cells. The scratch assay revealed that the migration rate of A549 and H1299 cells was markedly reduced following MTCH2 knockdown (**Figure 14B**). A subsequent Transwell assay confirmed that both migration and invasion capabilities of these cell lines were markedly suppressed after MTCH2 knockdown (**Figure 14C**), indicating that MTCH2 might be essential in the processes of tumor cell invasion and metastasis. Based on previous KEGG and GSEA analyses, it was identified that the cell cycle pathway exhibited significant enrichment in the group with elevated MTCH2 expression, which prompted further investigation into cell cycle alterations. Cell cycle analysis revealed that, following MTCH2 knockdown, there was a marked increase in the percentage of H1299 cells residing in the G0/G1 phase, concomitantly with a reduction in the percentage of cells in the S phase. This shift resulted in a notable arrest in the G0/G1 phase, as illustrated in **Figure 14E**.

## Discussion

LUAD is the most common subtype of NSCLC (55). Notwithstanding the recent progress in therapeutic approaches, the outlook for individuals diagnosed with LUAD continues to be unfavorable (56). Mitochondria, as critical intracellular organelles, fulfill various essential functions, including calcium homeostasis, energy metabolism, and apoptosis. These organelles produce ATP through oxidative phosphorylation and play a regulatory role in cellular metabolic pathways (57). Prior studies have indicated that mitochondrial dysfunction and metabolic reprogramming are key mechanisms that support tumor cell survival and proliferation (58). Therefore, investigating the relationship between MD and LUAD is not only vital for understanding the biological characteristics of the disease but may also inform novel therapeutic strategies.

In this study, we systematically analyzed transcriptional and genetic alterations associated with MD in LUAD by integrating somatic mutation data, RNA expression data, and clinical features from GEO and TCGA databases. Specifically, we employed expression data derived from a total of 541 patients diagnosed with LUAD and 59 normal control individuals retrieved from the TCGA database. Additionally, we incorporated data from 117 LUAD patients available in the GSE13213 dataset to establish a thorough expression matrix. Utilizing the expression profiles of 23 genes associated with MD, LUAD patients were classified into different molecular subgroups by consensus clustering analysis to further investigate their roles in tumor biology. In addition, we identified DEGs and their associated biological pathways by enrichment analysis, and developed a prognostic model for evaluating the effects of these key DEGs on patient prognosis, TME, and drug sensitivity. Meanwhile, the investigation additionally concentrated on the distinctive function of MTCH2, a crucial gene associated with MD, in LUAD along with its potential underlying mechanisms. The outcomes underscore the significant prognostic value of MD in LUAD, thereby establishing a robust basis for an enhanced comprehension of the biological processes and patient outcomes associated with LUAD, while also facilitating the formulation of targeted therapeutic approaches in the future.

In this study, LASSO regression was selected as the feature selection method due to its stability and widespread application in high-dimensional biological data. Compared with other commonly used methods, LASSO offers distinct advantages: it produces more stable and parsimonious models than decision tree algorithms (59, 60); it provides greater computational efficiency and interpretability than random forests, particularly in high-dimensional, low-sample-size contexts (61); and it is less sensitive to parameter tuning than support vector machines, making it well suited for high-throughput feature selection and prognostic modeling tasks (62, 63). Therefore, LASSO was deemed a reasonable and preferred choice in this study, particularly for modeling linear relationships with strong interpretability.

The analysis of immune cell infiltration in LUAD revealed significant differences across molecular subgroups, particularly in the levels of activated B cells, dendritic cells, and macrophages. Activated B cells are essential components of the adaptive immunity, enhancing antitumor responses by producing antibodies and memory cells. B cell infiltration has been shown to improve survival prognosis in cancers such as LUAD through T cell activation and coordination of antitumor responses (64). In this study, the elevated level of activated B cells in the B subtype likely contributes to enhanced overall survival. Dendritic cells, essential for initiating and regulating immune responses via antigen presentation, generally indicate active antitumor immunity within the TME. Research has demonstrated that dendritic cells can present exogenous antigens to CD8+ T cells through cross-presentation, a mechanism particularly important for antitumor immunity (65, 66). The substantial infiltration of activated dendritic cells in LUAD subtype B suggests a robust antitumor immune response, reinforcing the association between this subtype and improved survival. Macrophages, notably the M1 subtype, are recognized for their proinflammatory properties and ability to phagocytose tumor cells. The presence of M1 macrophages within the TME has been associated with improved prognostic outcomes in multiple cancer types. This correlation is attributed to their capacity to amplify T cell responses and suppress tumor proliferation via the secretion of cytokines (67). This study observed increased macrophage infiltration in the B subtype, suggesting a protective role of these immune cells in LUAD, potentially contributing to improved survival in this subgroup.

Enrichment analysis identified significant pathways involved in the DEGs of MD subtypes in LUAD. Cell cycle pathways are particularly crucial in the advancement of tumors, as the improper regulation of cell cycle mechanisms frequently leads to the unchecked growth and persistence of cancerous cells. The abnormal expression of cell cycle regulatory proteins, particularly Cyclin D1 and CDK4/6, has often been linked to the onset of different types of cancers (68). Additionally, significant enrichment of the P53 signaling pathway underscores its essential role in maintaining genomic stability and regulating apoptosis. As the “guardian of the genome,” P53 is often inactivated in cancer, contributing to the survival of malignant cells (69). The enrichment of P53 signaling-related genes in the MD subtype suggests that these tumors may evade apoptosis via this pathway, thereby facilitating tumor development.

The predictive model validation demonstrated significant predictive power for OS in LUAD patients. Through both univariate and multivariate Cox regression analyses, five essential genes were identified for the formulation of the risk score. The efficacy of the model was validated in both training and testing cohorts, with Kaplan-Meier survival analysis revealing a statistically significant disparity in survival rates between the low-risk and high-risk groups (p < 0.001). The AUC values at 1, 3, and 5 years were 0.767, 0.721, and 0.744 for the training group and 0.690, 0.644, and 0.615 for the testing group, indicating robust patient risk stratification. Incorporating clinical variables (age, gender, tumor stage) further enhanced predictive accuracy, as supported by the calibration curve. Additionally, connections between the risk score, TMB, and drug sensitivity underscore its utility in treatment decision-making. Association between the risk score and TME characteristics, particularly immune cell infiltration, suggests high-risk patients may exhibit a more immunosuppressive TME. This observation informs anti-tumor immunotherapy strategies for high-risk LUAD patients. Overall, these findings deepen understanding of LUAD’s molecular characteristics and underscore MD’ critical role in disease progression.

It is noteworthy that patients in the high risk group generally exhibit poorer survival outcomes, likely due to increased proliferative capacity, elevated tumor stemness indices, and greater genomic instability. Furthermore, marked differences in immune cell infiltration were observed across risk groups, with the low risk group displaying enhanced immune responsiveness, which may contribute to its more favorable prognosis. These findings highlight the potential prognostic relevance of MD in LUAD from an immunobiological perspective and provide a theoretical basis for precise molecular subtyping and the development of personalized immunotherapeutic strategies.

The role of MTCH2, a key MD gene, was further investigated in LUAD. Elevated MTCH2 expression in LUAD MD samples, as compared to normal tissue, indicates its potential involvement in tumorigenesis. The Kaplan-Meier survival analysis demonstrated a significant correlation between elevated MTCH2 expression and unfavorable outcomes in patients with LUAD, highlighting its prospective utility as a prognostic biomarker. Additionally, the correlation between MTCH2 expression levels and clinical staging parameters (N stage, T stage, and overall clinical stage) reinforces its role in cancer progression. Higher MTCH2 expression was observed in patients with advanced stages (T4, N2, stage III), suggesting its possible contribution to tumor aggressiveness and metastatic potential. *In vitro* experiments further confirmed that MTCH2 is significantly upregulated in LUAD cells and tissues, where it promotes proliferation, migration, invasion, and cell cycle progression. The results indicate that the inhibition of MTCH2 may serve as a potentially effective approach for the creation of innovative treatment options.

Additionally, other MD-related genes play important roles. MFN2 is critically involved in mitochondrial fusion and cellular metabolism. Zhang et al. reported that low MFN2 expression in LUAD is associated with poor clinical outcomes. MFN2 and UCP4 may jointly regulate calcium homeostasis in LUAD and potentially serve as therapeutic targets (70). These findings suggest that MFN2 may function as a valuable prognostic biomarker and therapeutic target, as its upregulation may help restore mitochondrial function and suppress tumor growth. Our results are consistent with observations in other cancer types. Cheng et al. reported that MFN2 is downregulated in colorectal cancer and may exert tumor-suppressive effects by regulating apoptosis and metabolic pathways, supporting its prognostic and therapeutic relevance (71). Song et al. demonstrated that MFN1-mediated mitochondrial fusion promotes tamoxifen resistance by inhibiting apoptosis, underscoring the critical role of MD-related genes in breast cancer drug resistance. Collectively, these findings suggest that MD abnormalities may contribute to malignant progression in various cancers through metabolic reprogramming and immune modulation.

Although this study identified key MD genes and their associations with LUAD subtypes, certain limitations remain. First, functional validation of these genes is limited in both *in vitro* and *in vivo* contexts. While cell line experiments offer preliminary insights, they do not fully replicate the complexity of tumor behavior in the human body.

## Conclusions

This study performed an in-depth examination of LUAD utilizing data from the TCGA and GEO databases, integrating RNA expression, mutation profiles, and clinical attributes. Findings revealed that significant transcriptional changes in MD genes delineated two molecular subtypes linked to survival outcomes. The constructed prognostic model demonstrated strong predictive performance in both training and test cohorts.

## Data Availability

The original contributions presented in the study are included in the article/**Supplementary Material**. Further inquiries can be directed to the corresponding authors.

## Supplementary material

Supplementary Figure S1 Flowchart of the study.

Supplementary Figure S2 KM curve results of MD genes.

Supplementary Figure S3
**(A)** Consensus matrix heatmaps and unsupervised clustering of MD genes for k = 3–9. **(B)** Tracking Plot of Sample Classification.

Supplementary Figure S4 MD subtypes in the LUAD cohort were identified as gene subtypes based on DEGs. **(A)** 217 MD subtype-related DEGs; **(B)** For each tested k, the CDF displays the cumulative fraction of every sample co-clustering at the specified consensus index (1.0 = co-clustered 100% of the time); **(C)** The consensus clustering delta area curve, which shows how the area under the CDF curve changes relative to k − 1 for each category number k; **(D)** The consensus matrix indicating the correlation area and clusters (k = 2-9); **(E)** Tracking Plot of Sample Classification.

Supplementary Figure S5 The six genes with the strongest positive correlation with MTCH2.

Supplementary Figure S6 Correlation graph between MTCH2 and immune cells.

Supplementary Figure S7 Relationship between MTCH2 and drug sensitivity.
