# Supplementary figures and images for: Integrated multi-omics analysis and experimental investigation of mitochondrial dynamics-related genes: molecular subtypes, immune landscape, and prognostic implications in lung adenocarcinoma
Source: Front Immunol. 2025 May 29;16:1585505. doi: 10.3389/fimmu.2025.1585505 (PMC12159055; doi:10.3389/fimmu.2025.1585505)

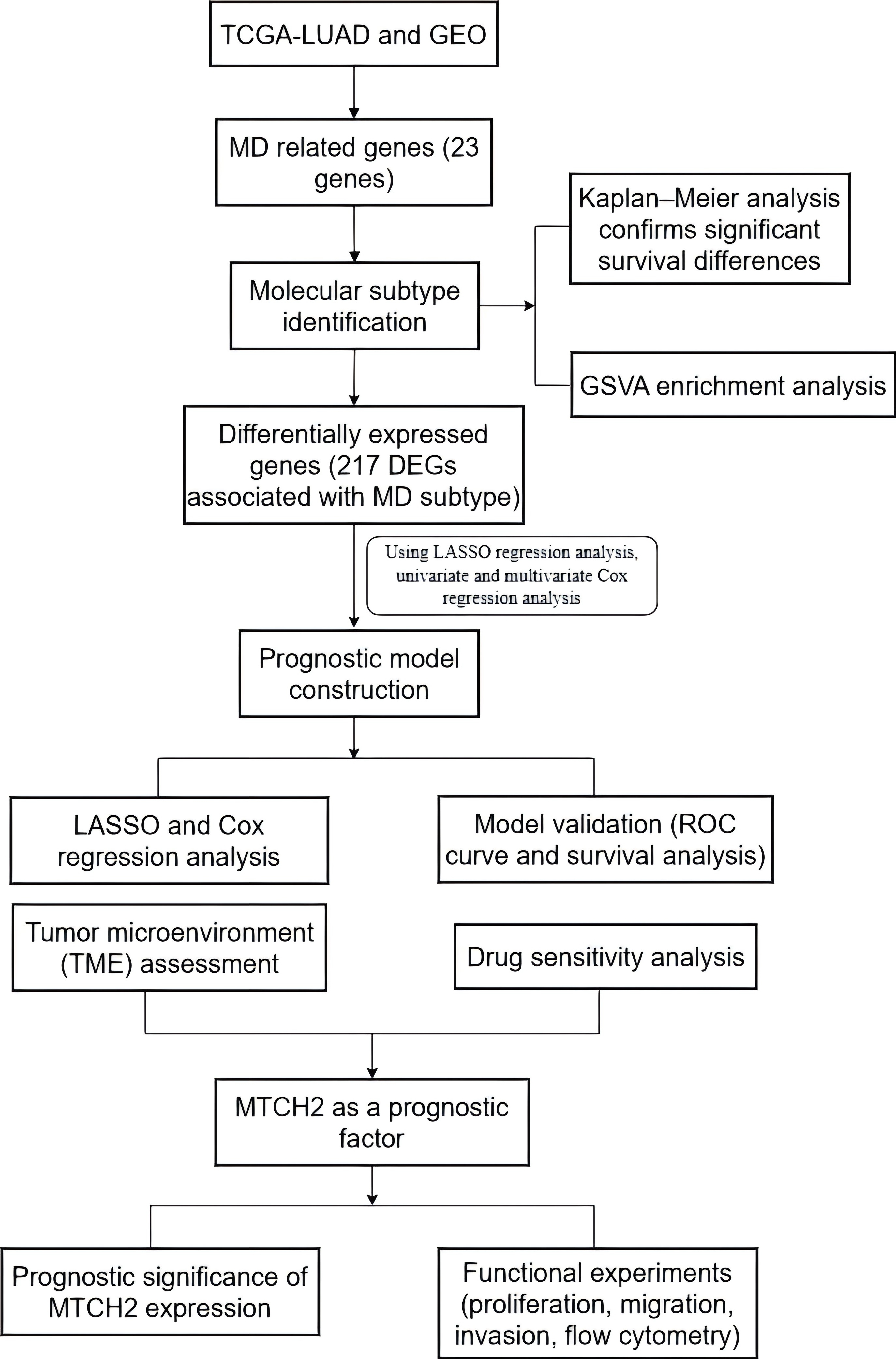

Supplement: Supplementary Figure S1 — Flowchart of the study. [file Image1.jpeg]

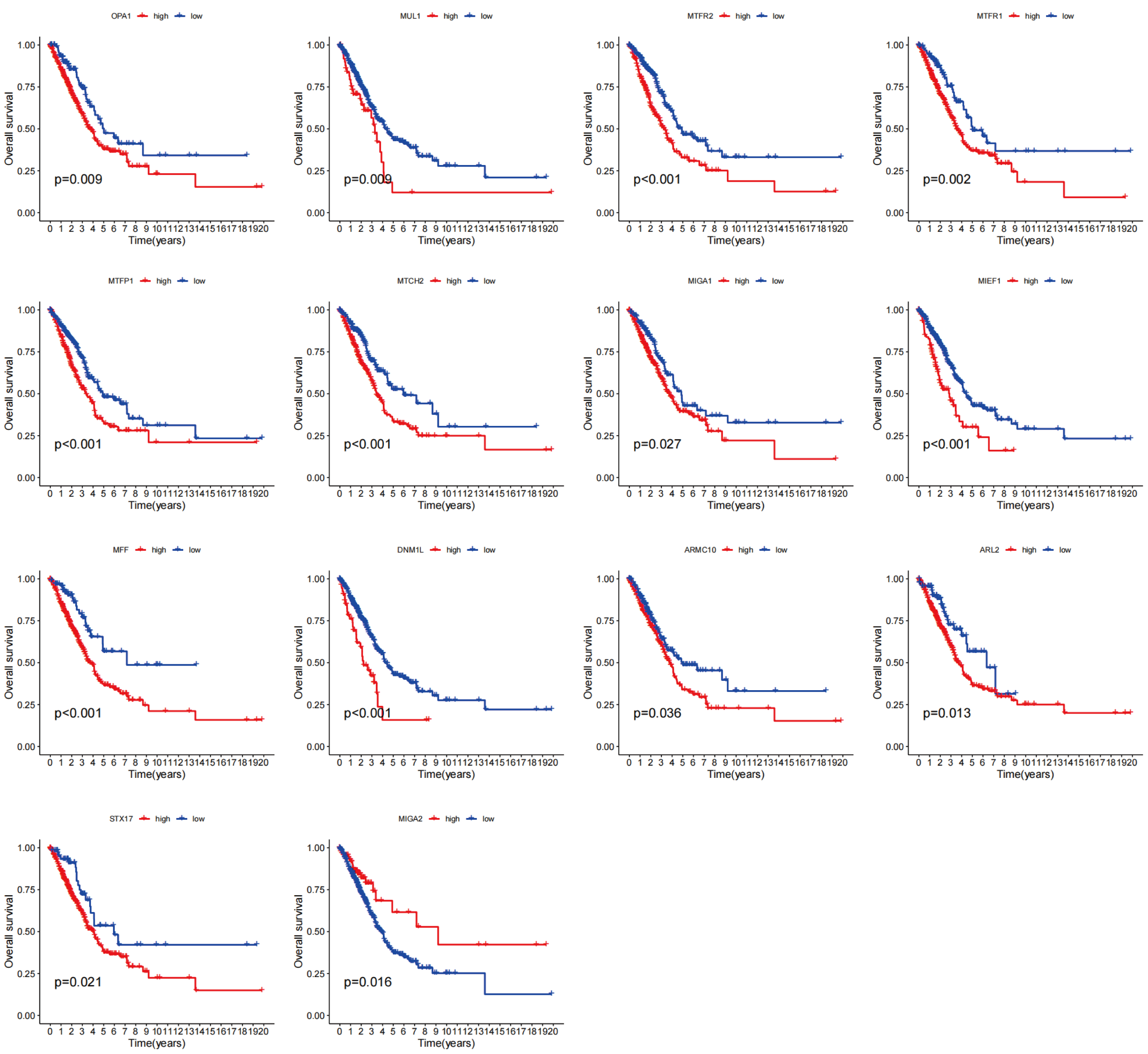

Supplement: Supplementary Figure S2 — KM curve results of MD genes. [file Image2.tif]

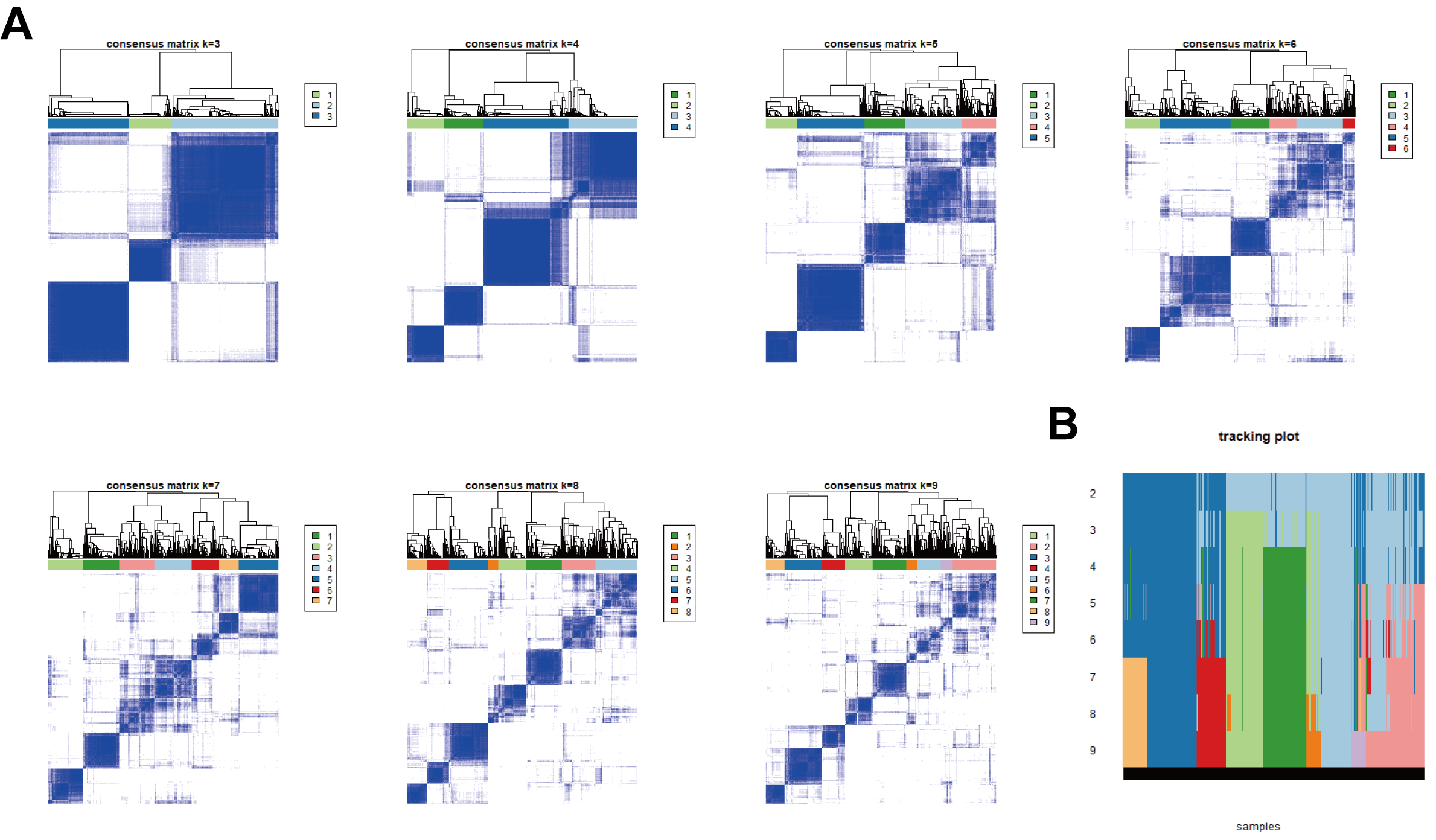

Supplement: Supplementary Figure S3 — (A) Consensus matrix heatmaps and unsupervised clustering of MD genes for k = 3–9. (B) Tracking Plot of Sample Classification. [file Image3.tif]

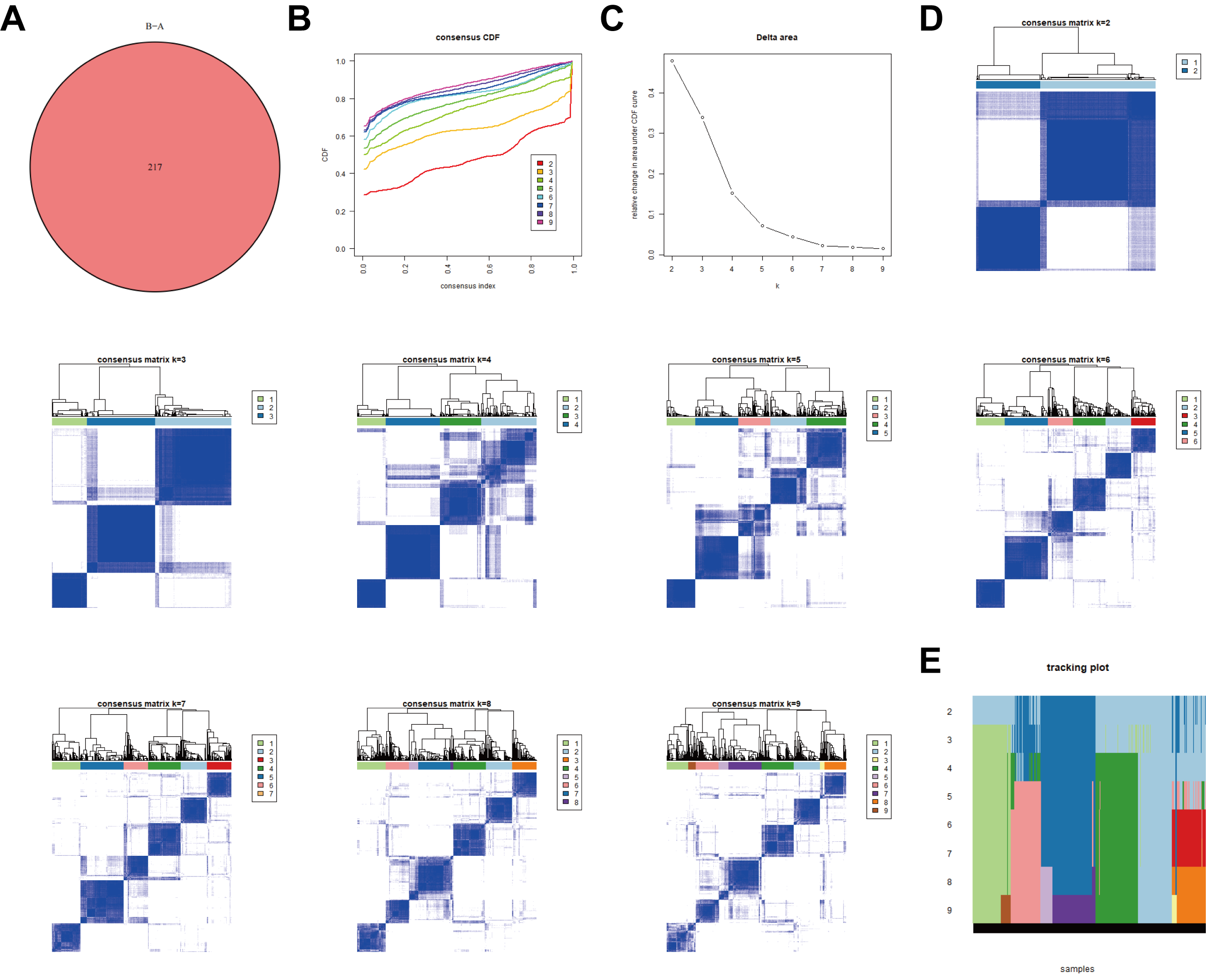

Supplement: Supplementary Figure S4 — MD subtypes in the LUAD cohort were identified as gene subtypes based on DEGs. (A) 217 MD subtype-related DEGs; (B) For each tested k, the CDF displays the cumulative fraction of every sample co-clustering at the specified consensus index (1.0 = co-clustered 100% of the time); (C) The consensus clustering delta area curve, which shows how the area under the CDF curve changes relative to k − 1 for each category number k; (D) The consensus matrix indicating the correlation area and clusters (k = 2-9); (E) Tracking Plot of Sample Classification. [file Image4.tif]

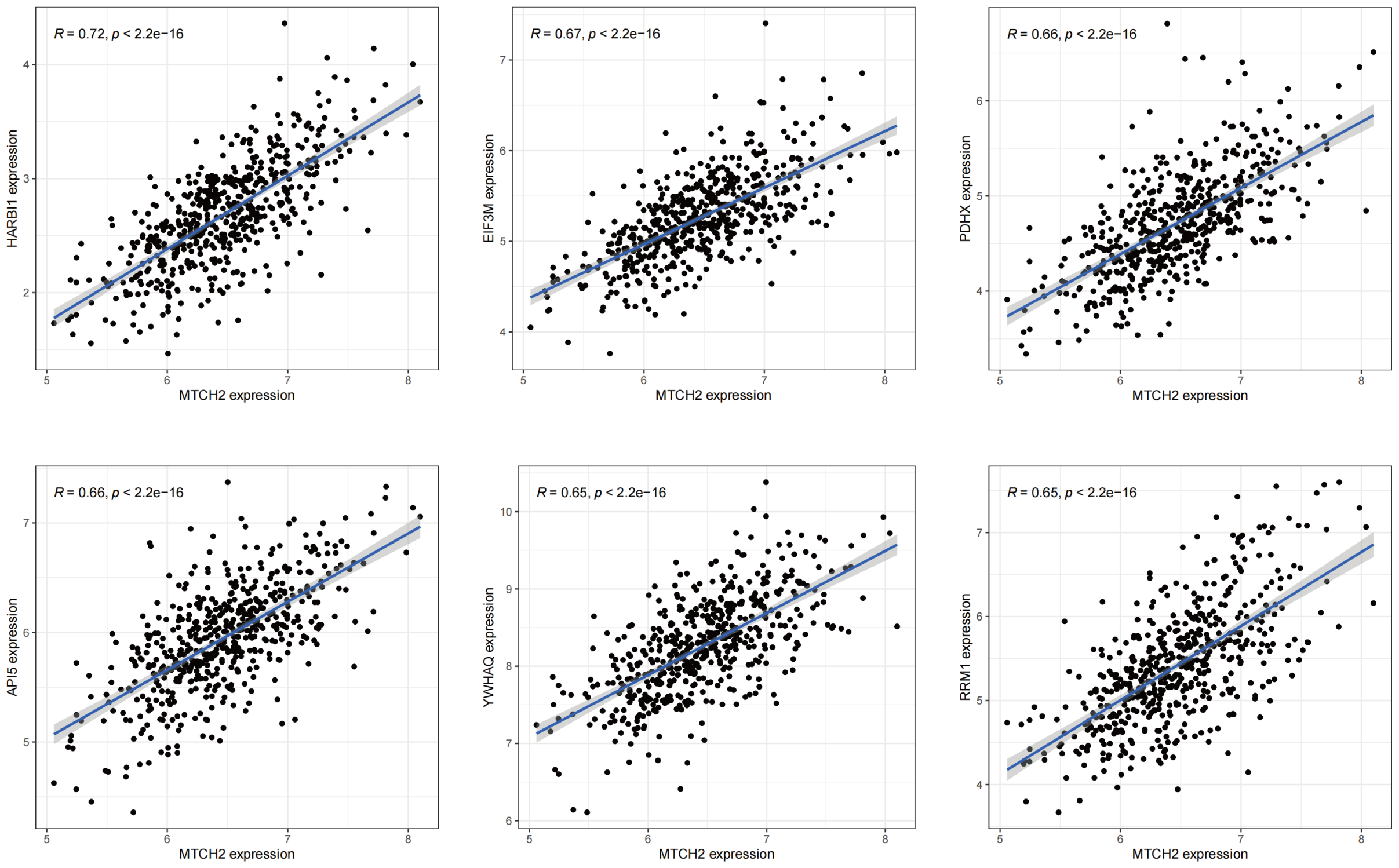

Supplement: Supplementary Figure S5 — The six genes with the strongest positive correlation with MTCH2. [file Image5.tif]

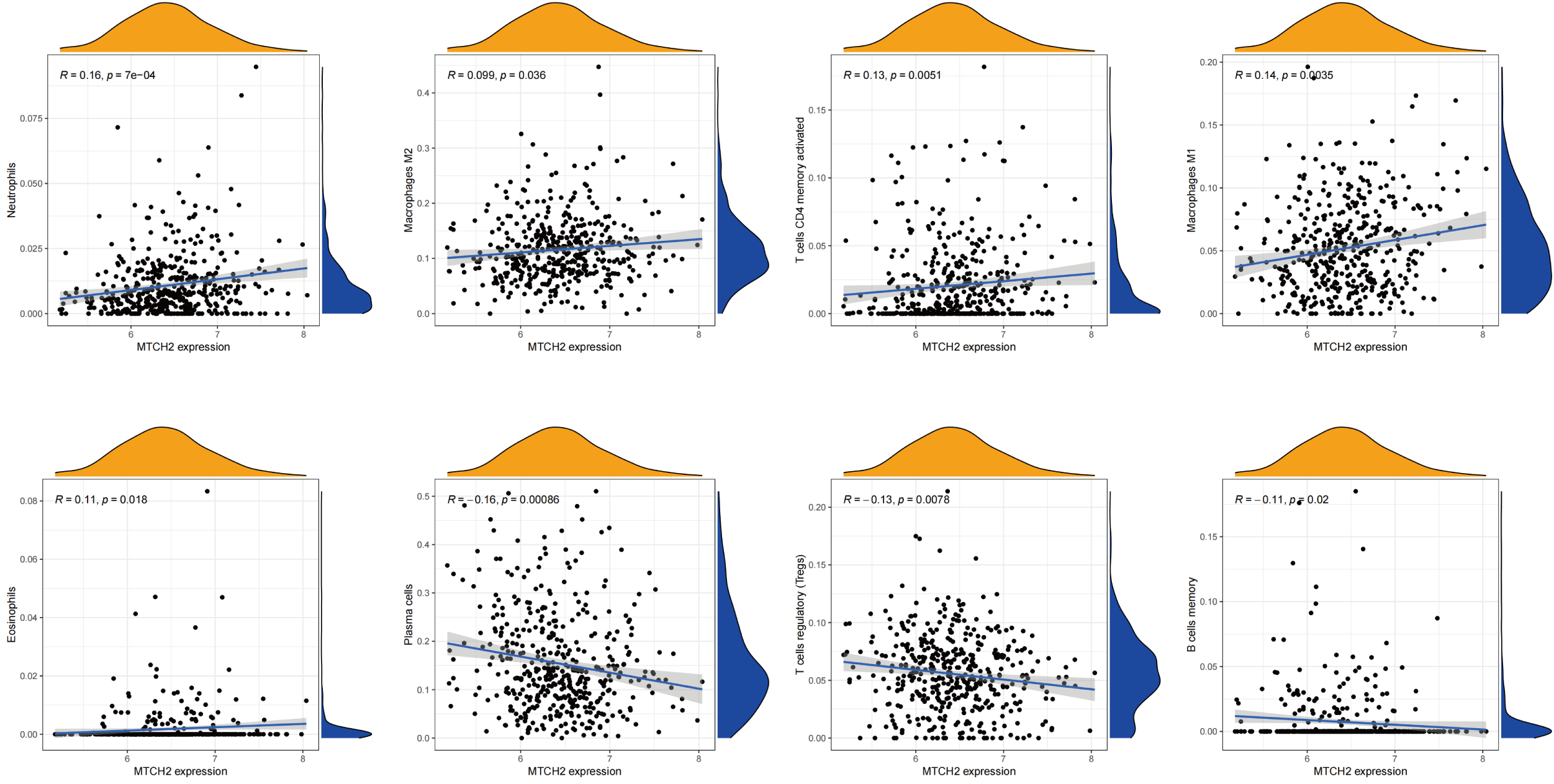

Supplement: Supplementary Figure S6 — Correlation graph between MTCH2 and immune cells. [file Image6.tif]

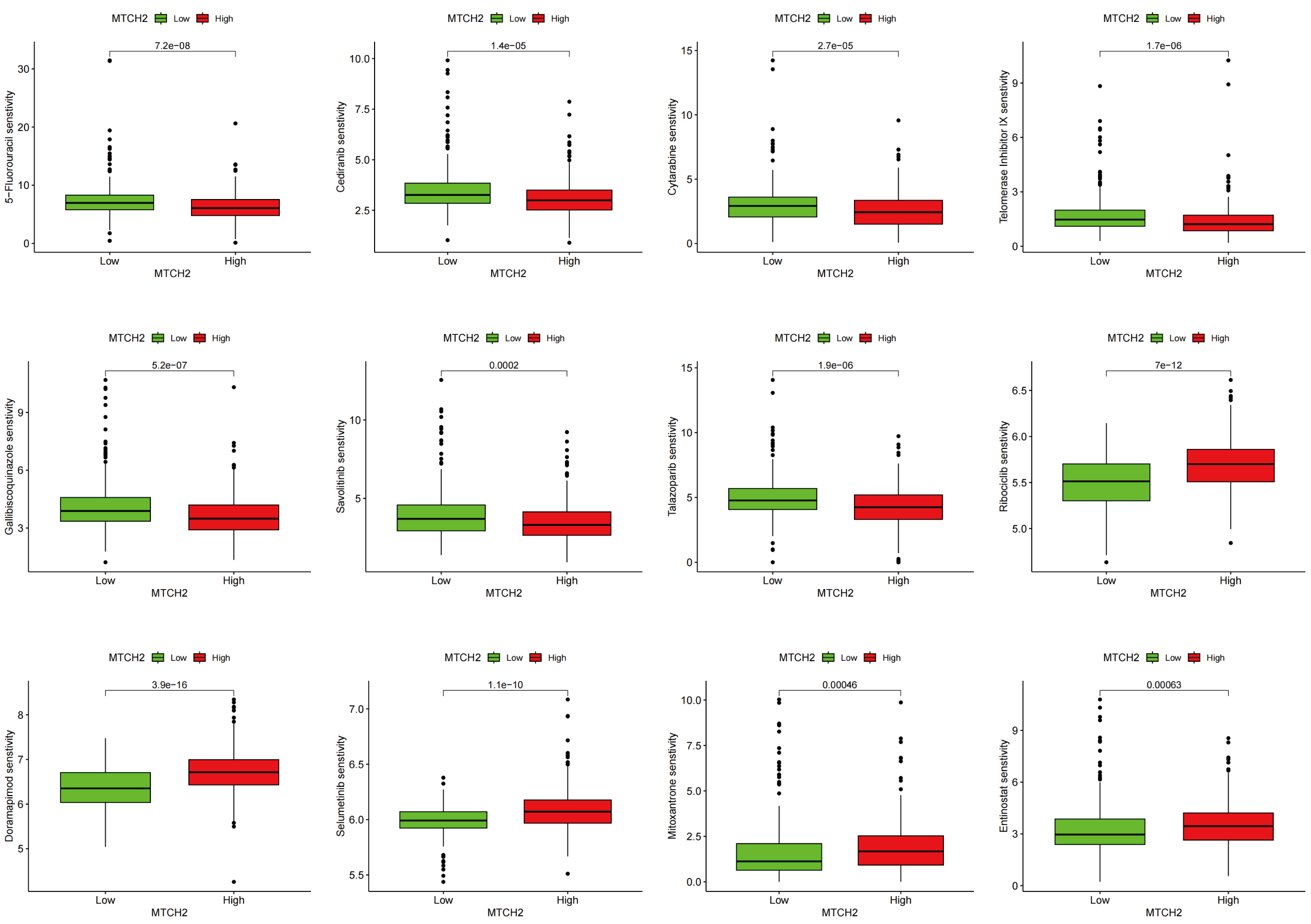

Supplement: Supplementary Figure S7 — Relationship between MTCH2 and drug sensitivity. [file Image7.tif]

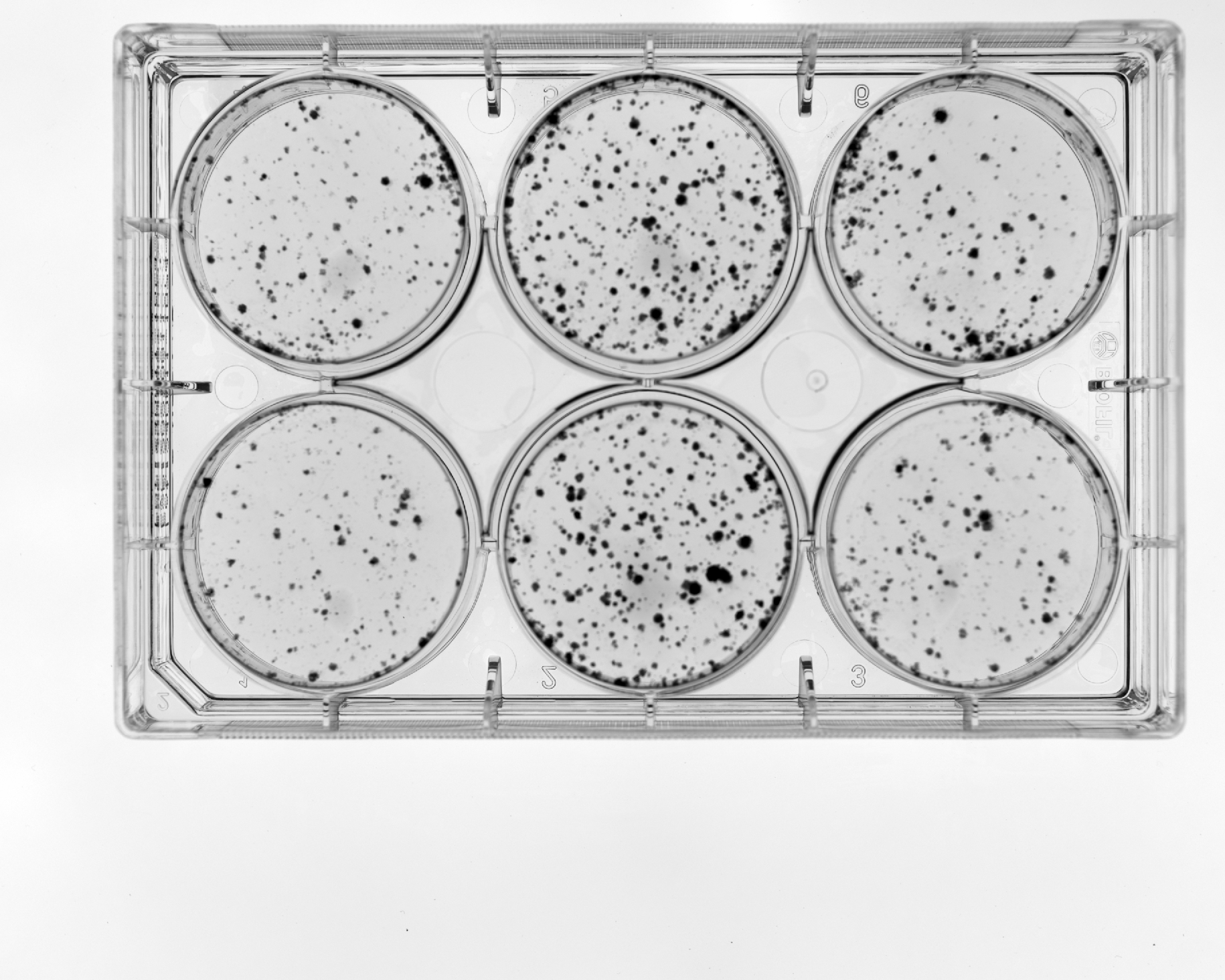

Supplement: Supplementary file 10 [file DataSheet3.zip › the raw images to Figures 14A, 14B, AND 14C/Colony Formation Assay/A549(Colony Formation Assay).jpg]

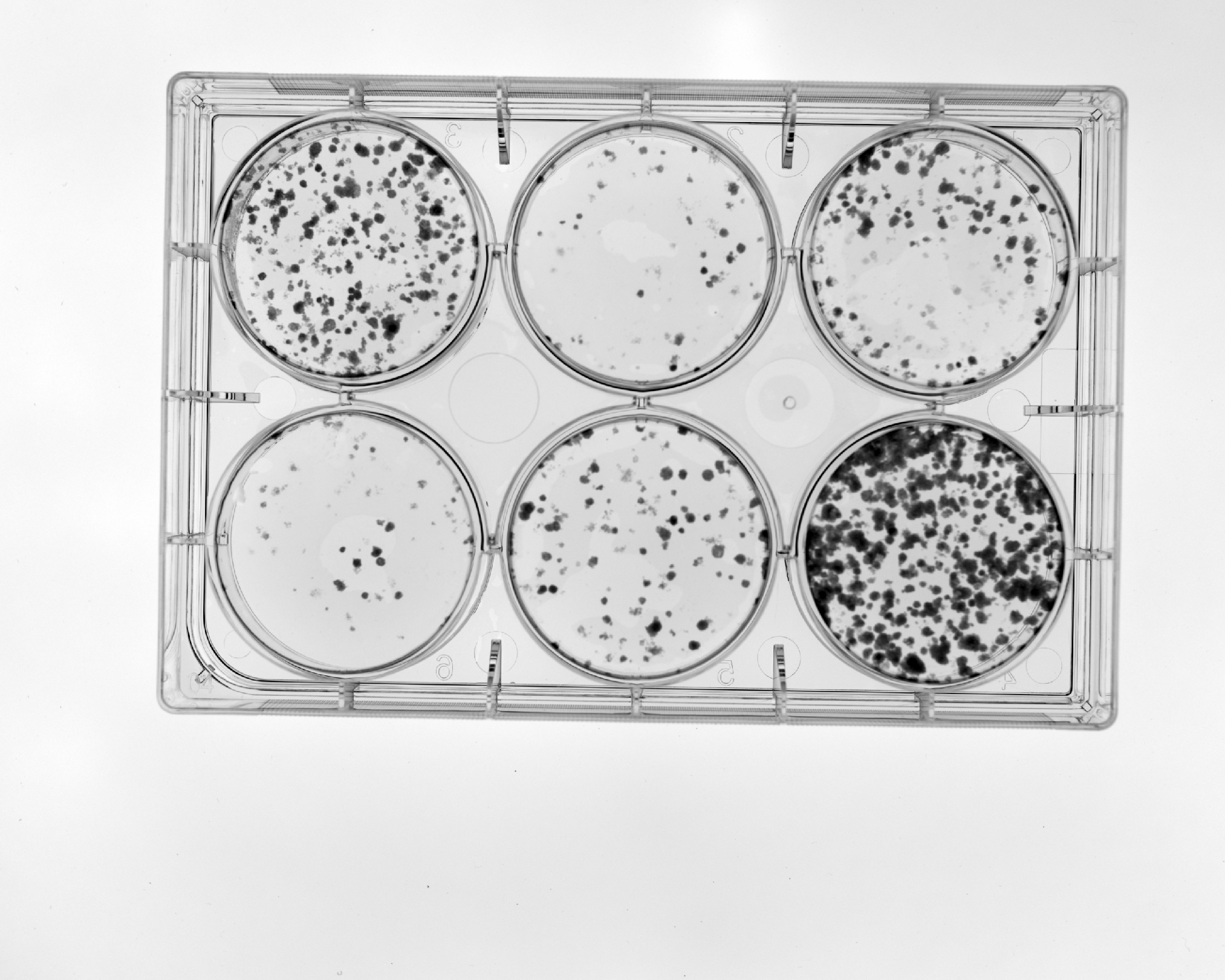

Supplement: Supplementary file 10 [file DataSheet3.zip › the raw images to Figures 14A, 14B, AND 14C/Colony Formation Assay/H1299(Colony Formation Assay).jpg]

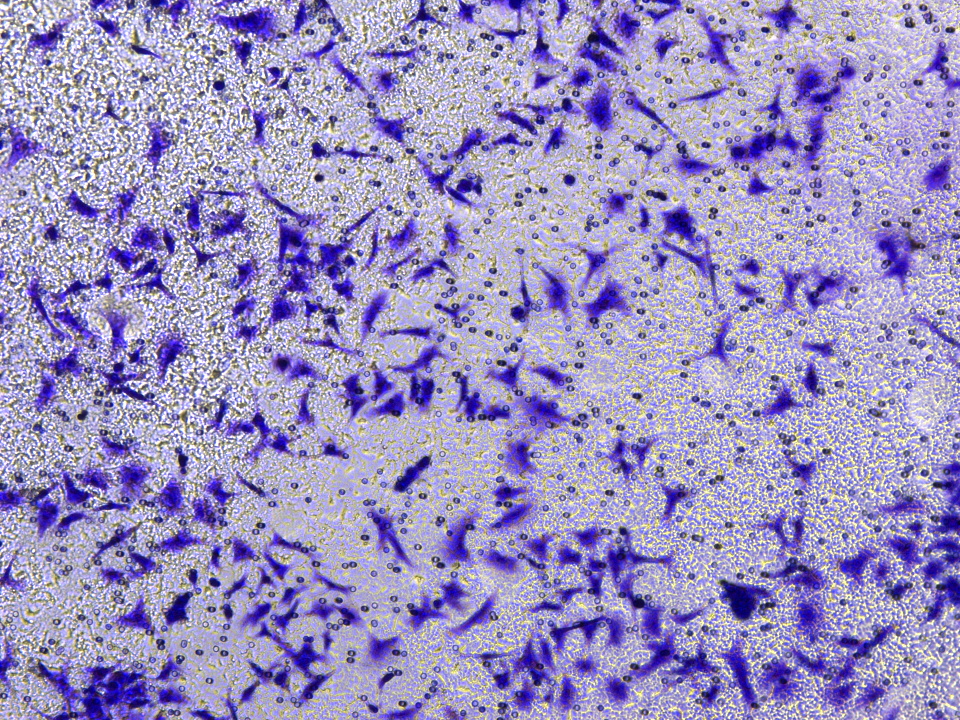

Supplement: Supplementary file 10 [file DataSheet3.zip › the raw images to Figures 14A, 14B, AND 14C/Transwell Assay/A549/Invasion/NC.tif]

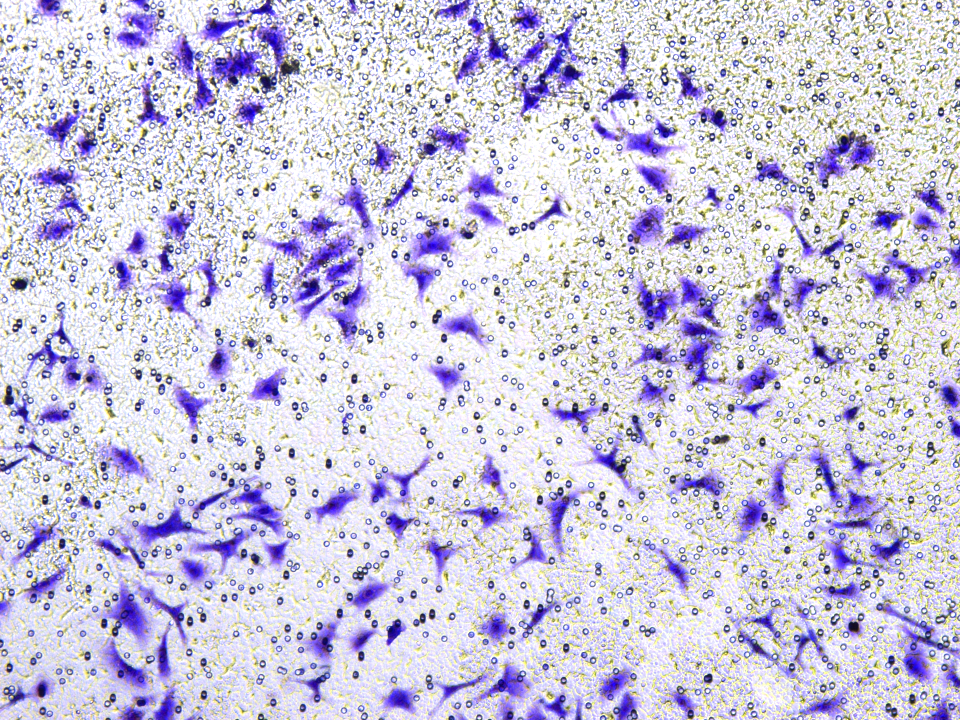

Supplement: Supplementary file 10 [file DataSheet3.zip › the raw images to Figures 14A, 14B, AND 14C/Transwell Assay/A549/Invasion/si-MTCH2-2.tif]

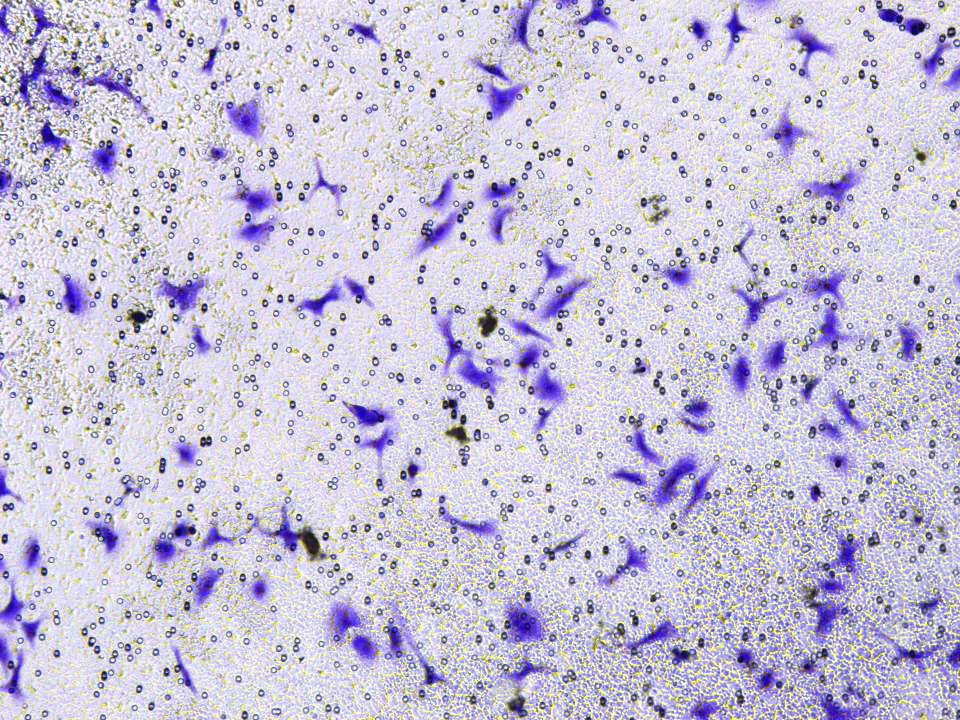

Supplement: Supplementary file 10 [file DataSheet3.zip › the raw images to Figures 14A, 14B, AND 14C/Transwell Assay/A549/Invasion/si-MTCH2-3.tif]

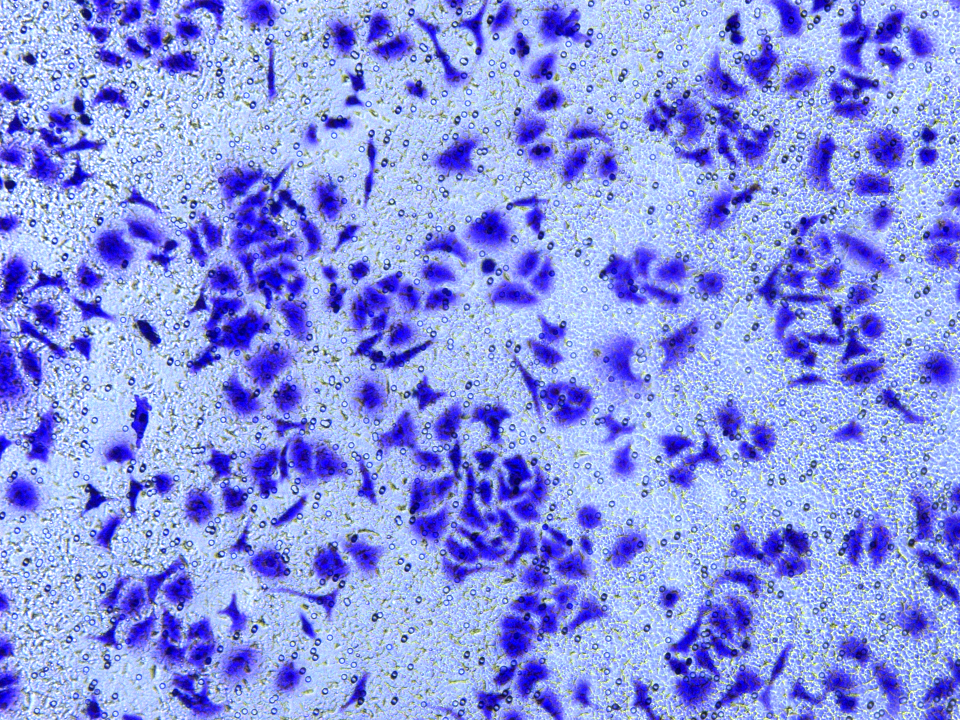

Supplement: Supplementary file 10 [file DataSheet3.zip › the raw images to Figures 14A, 14B, AND 14C/Transwell Assay/A549/Migration/NC.tif]

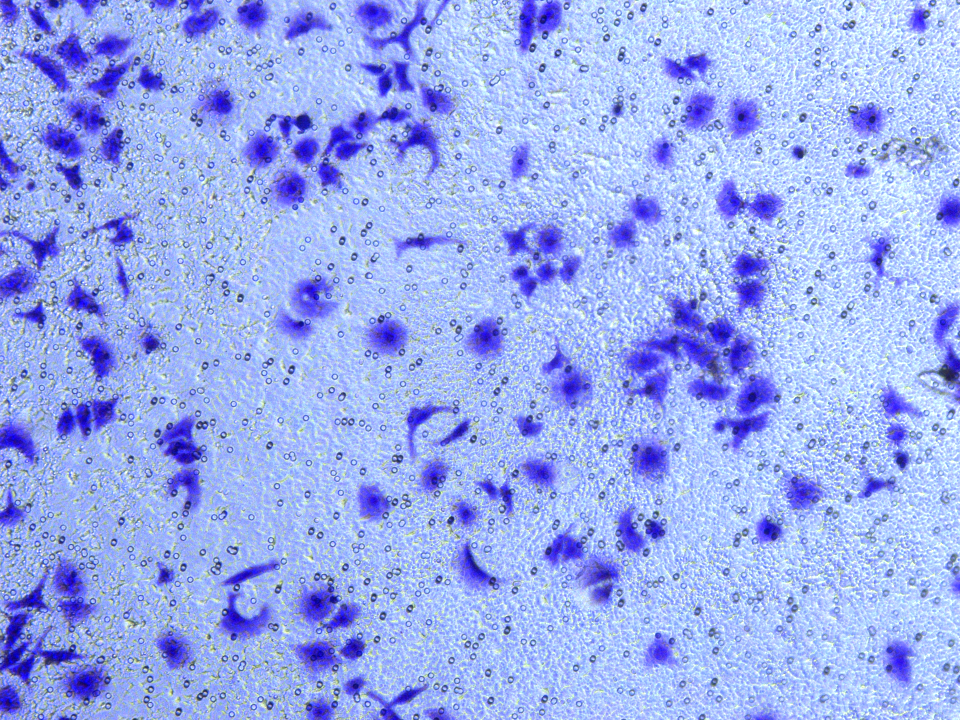

Supplement: Supplementary file 10 [file DataSheet3.zip › the raw images to Figures 14A, 14B, AND 14C/Transwell Assay/A549/Migration/si-MTCH2-2.tif]

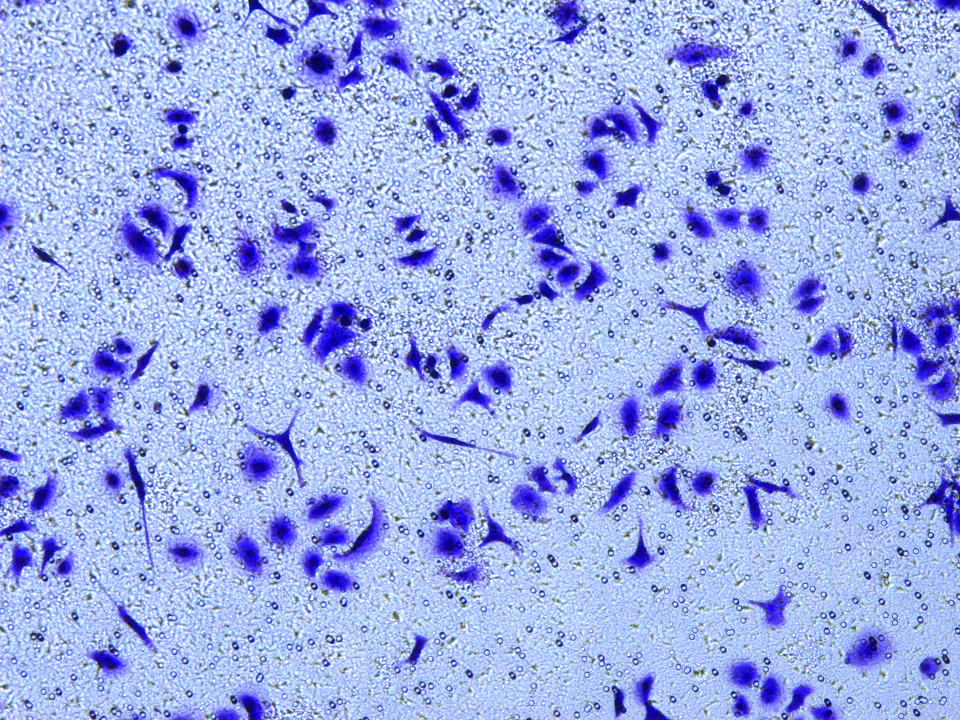

Supplement: Supplementary file 10 [file DataSheet3.zip › the raw images to Figures 14A, 14B, AND 14C/Transwell Assay/A549/Migration/si-MTCH2-3.tif]

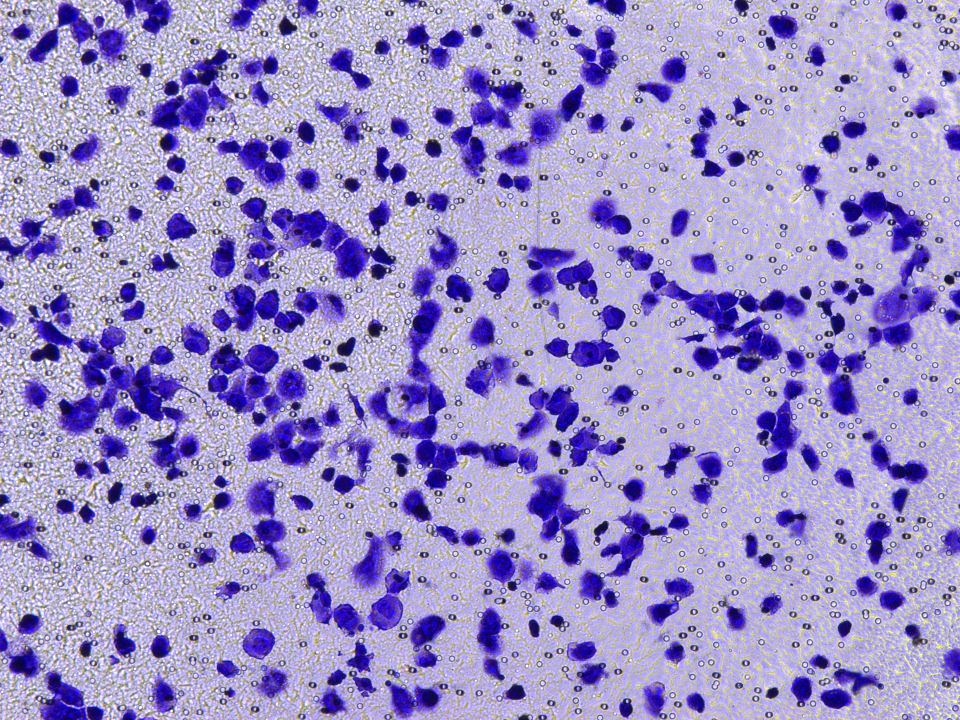

Supplement: Supplementary file 10 [file DataSheet3.zip › the raw images to Figures 14A, 14B, AND 14C/Transwell Assay/H1299/Invasion/NC.tif]

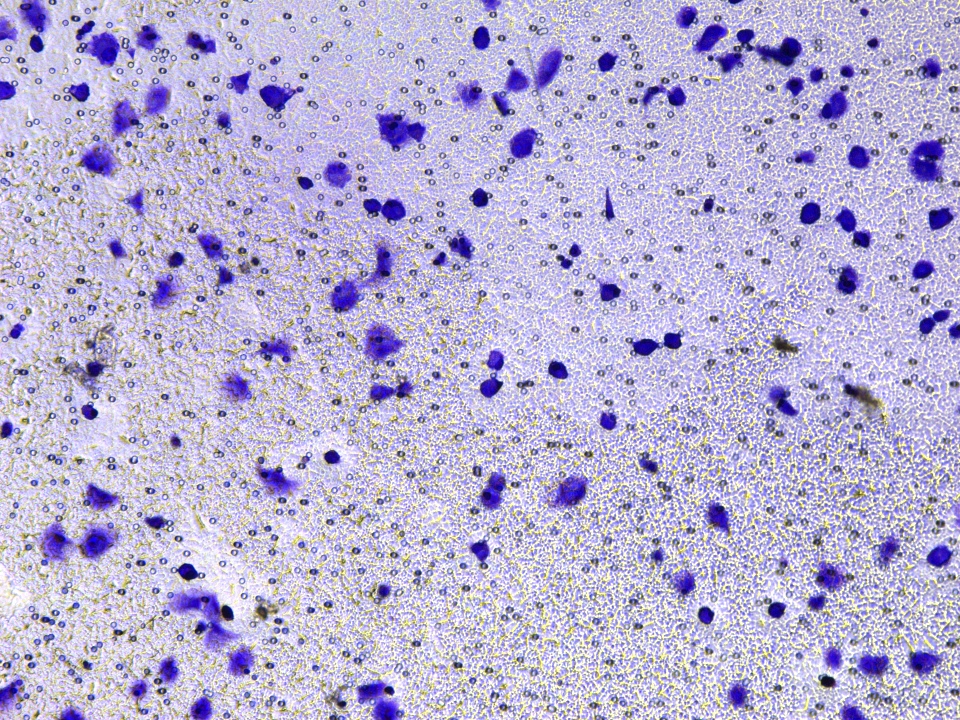

Supplement: Supplementary file 10 [file DataSheet3.zip › the raw images to Figures 14A, 14B, AND 14C/Transwell Assay/H1299/Invasion/si-MTCH2-2.tif]

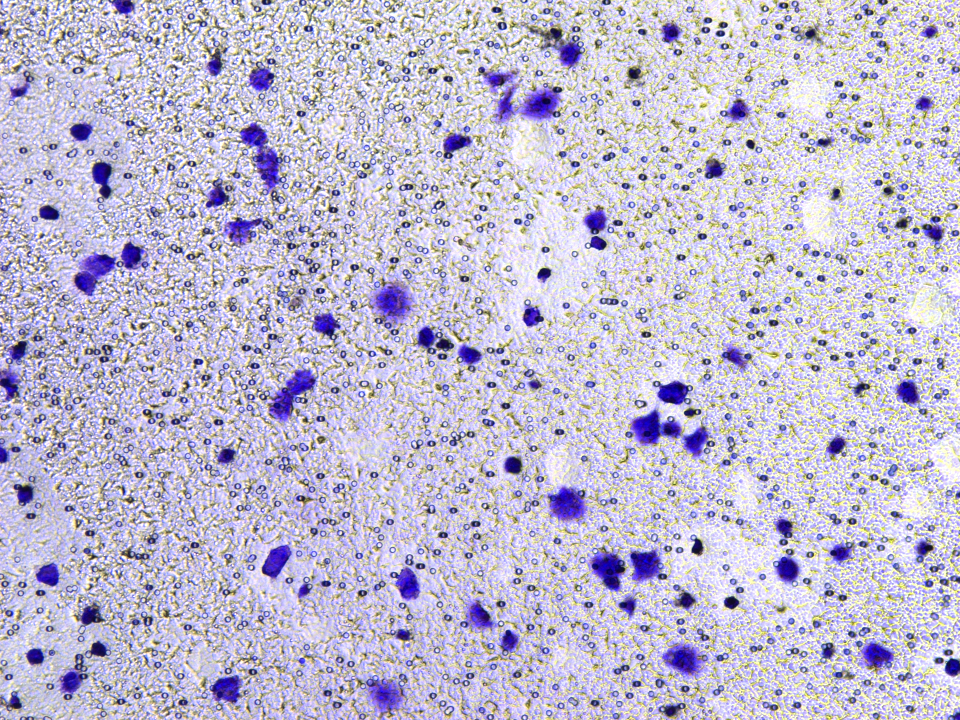

Supplement: Supplementary file 10 [file DataSheet3.zip › the raw images to Figures 14A, 14B, AND 14C/Transwell Assay/H1299/Invasion/si-MTCH2-3.tif]

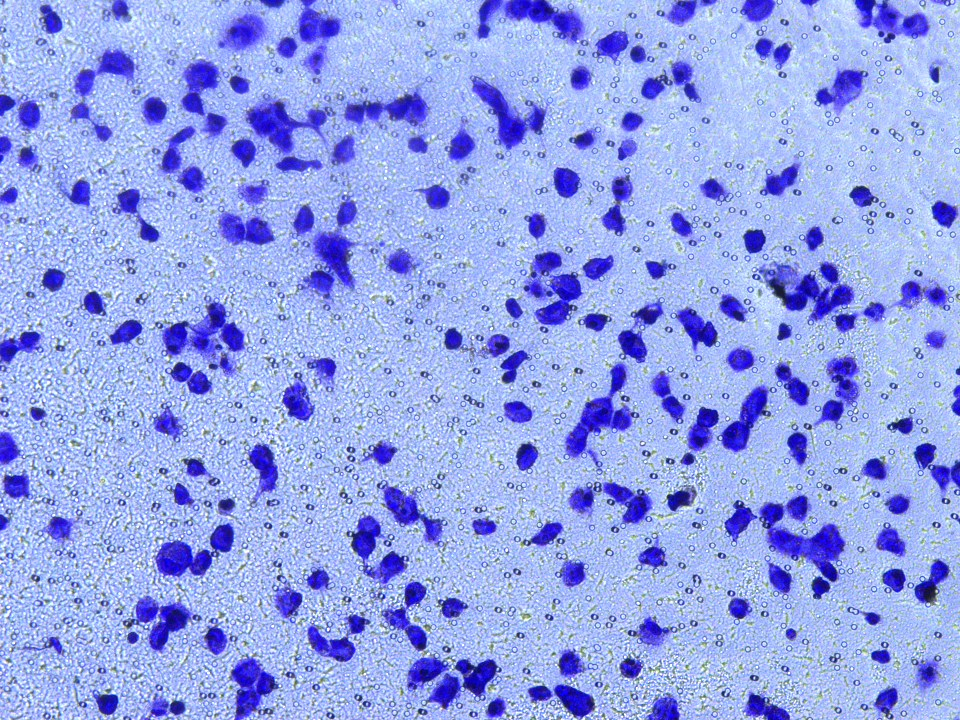

Supplement: Supplementary file 10 [file DataSheet3.zip › the raw images to Figures 14A, 14B, AND 14C/Transwell Assay/H1299/Migration/NC.tif]

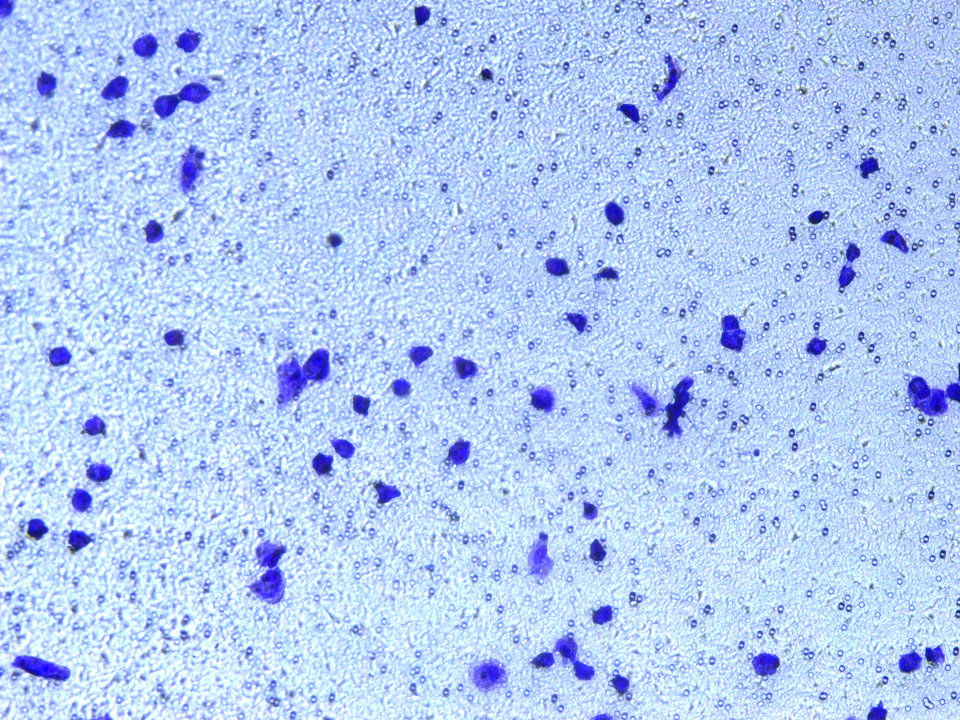

Supplement: Supplementary file 10 [file DataSheet3.zip › the raw images to Figures 14A, 14B, AND 14C/Transwell Assay/H1299/Migration/si-MTCH2-2.tif]

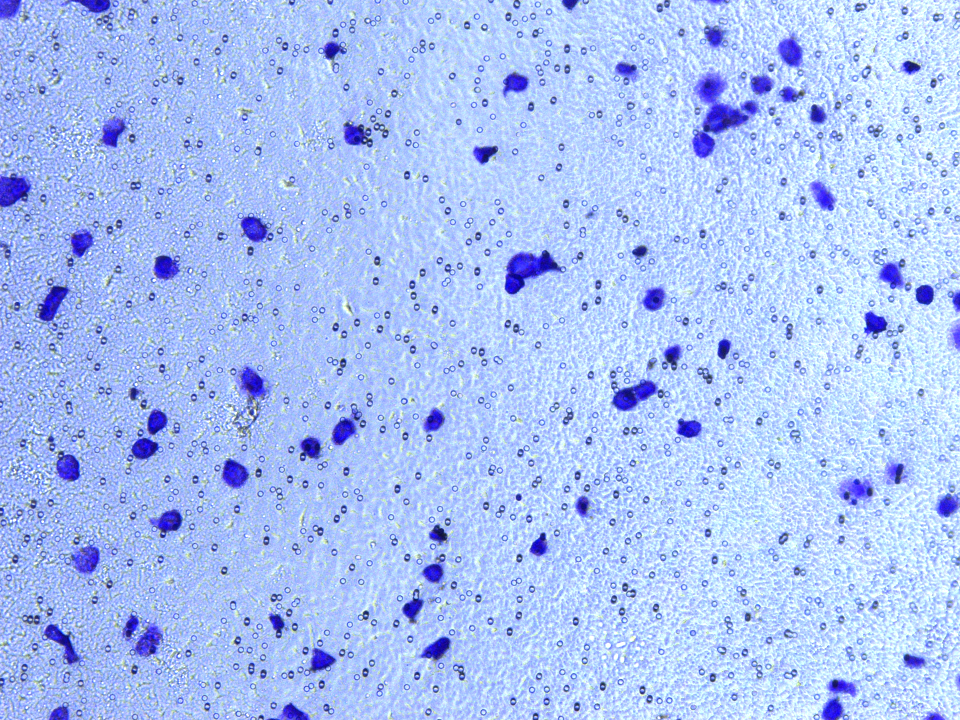

Supplement: Supplementary file 10 [file DataSheet3.zip › the raw images to Figures 14A, 14B, AND 14C/Transwell Assay/H1299/Migration/si-MTCH2-3.tif]

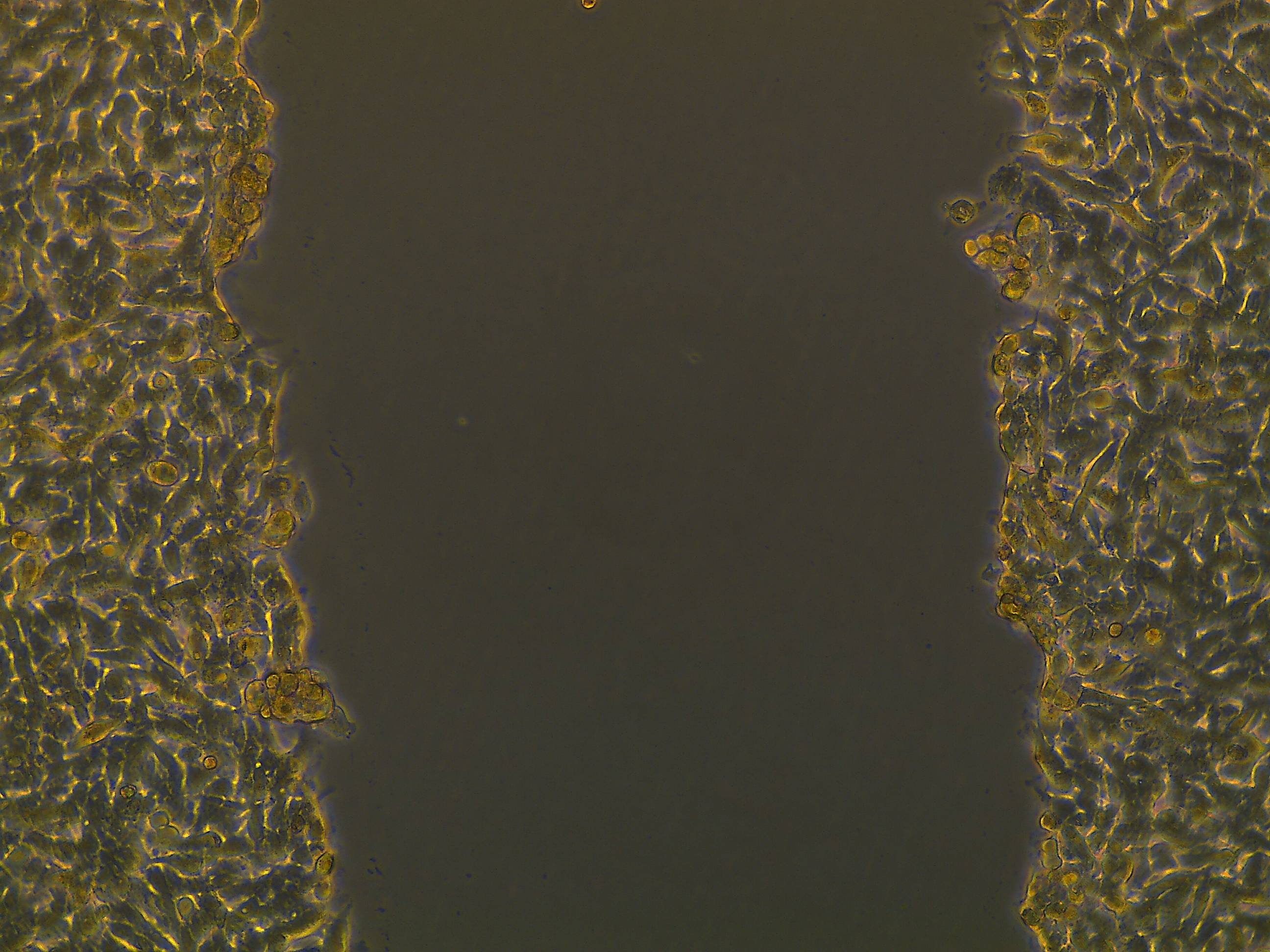

Supplement: Supplementary file 10 [file DataSheet3.zip › the raw images to Figures 14A, 14B, AND 14C/Wound Healing Assay/A549/0h(NC).jpg]

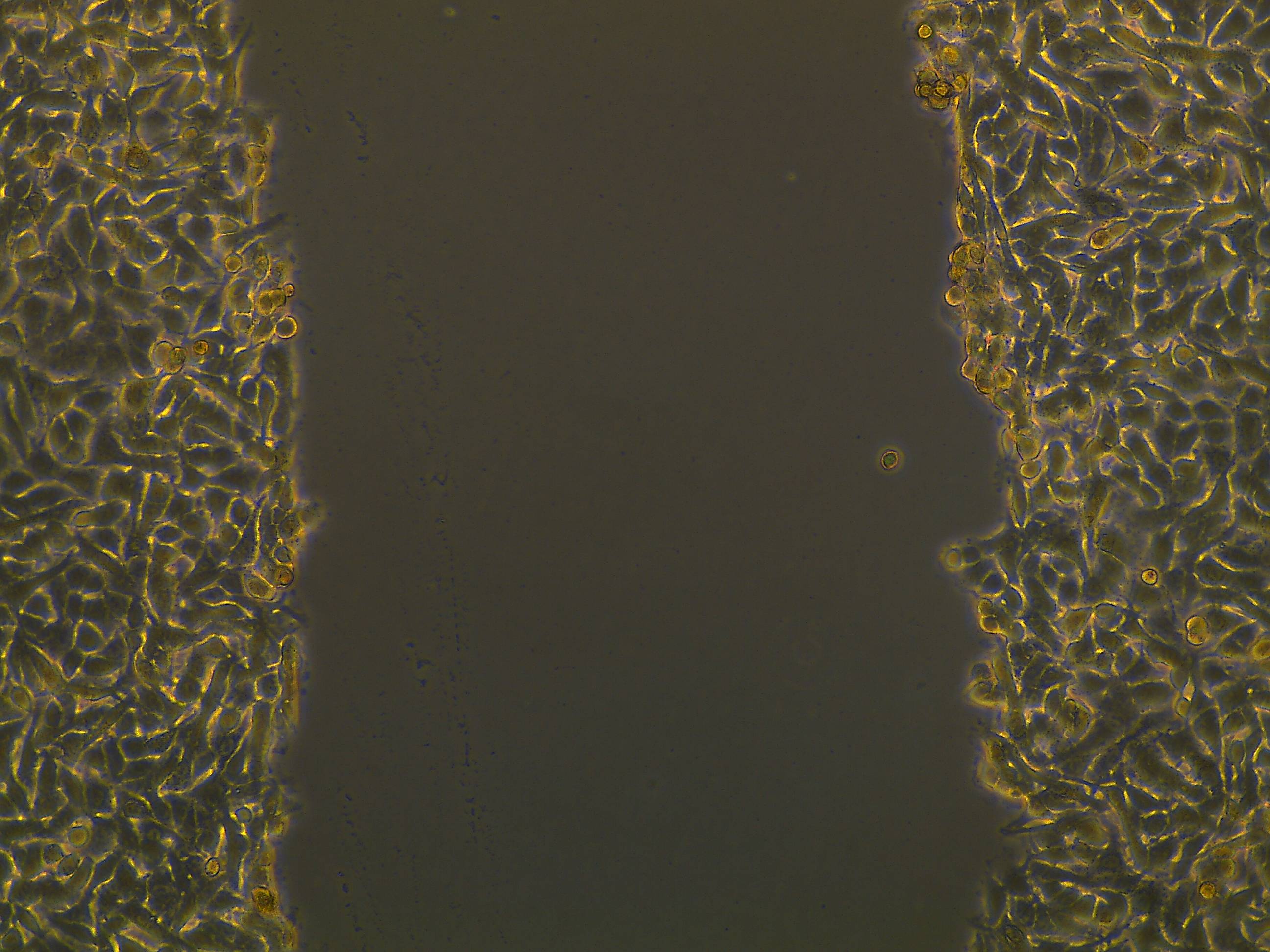

Supplement: Supplementary file 10 [file DataSheet3.zip › the raw images to Figures 14A, 14B, AND 14C/Wound Healing Assay/A549/0h(si-MTCH2-2).jpg]

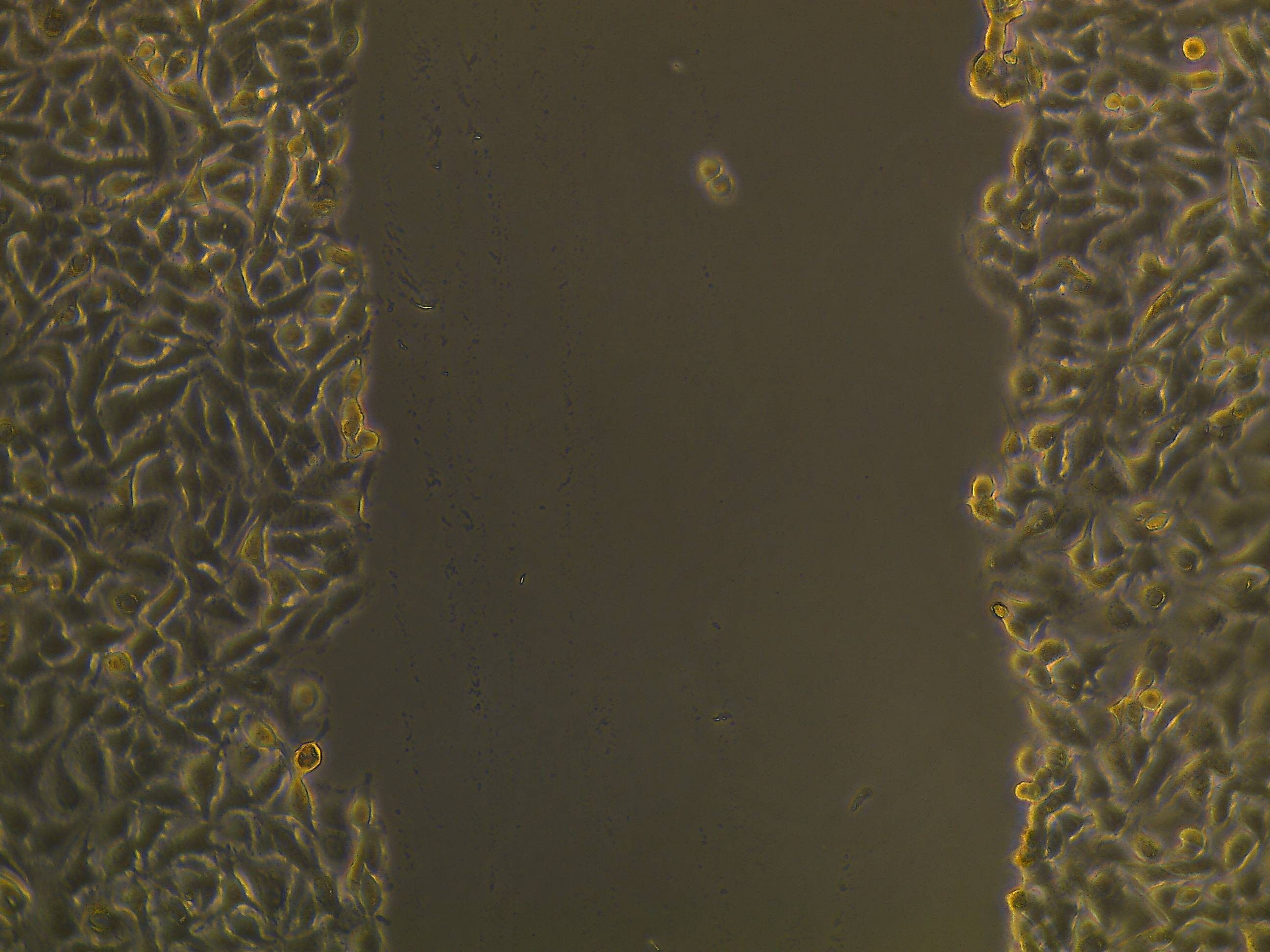

Supplement: Supplementary file 10 [file DataSheet3.zip › the raw images to Figures 14A, 14B, AND 14C/Wound Healing Assay/A549/0h(si-MTCH2-3).jpg]

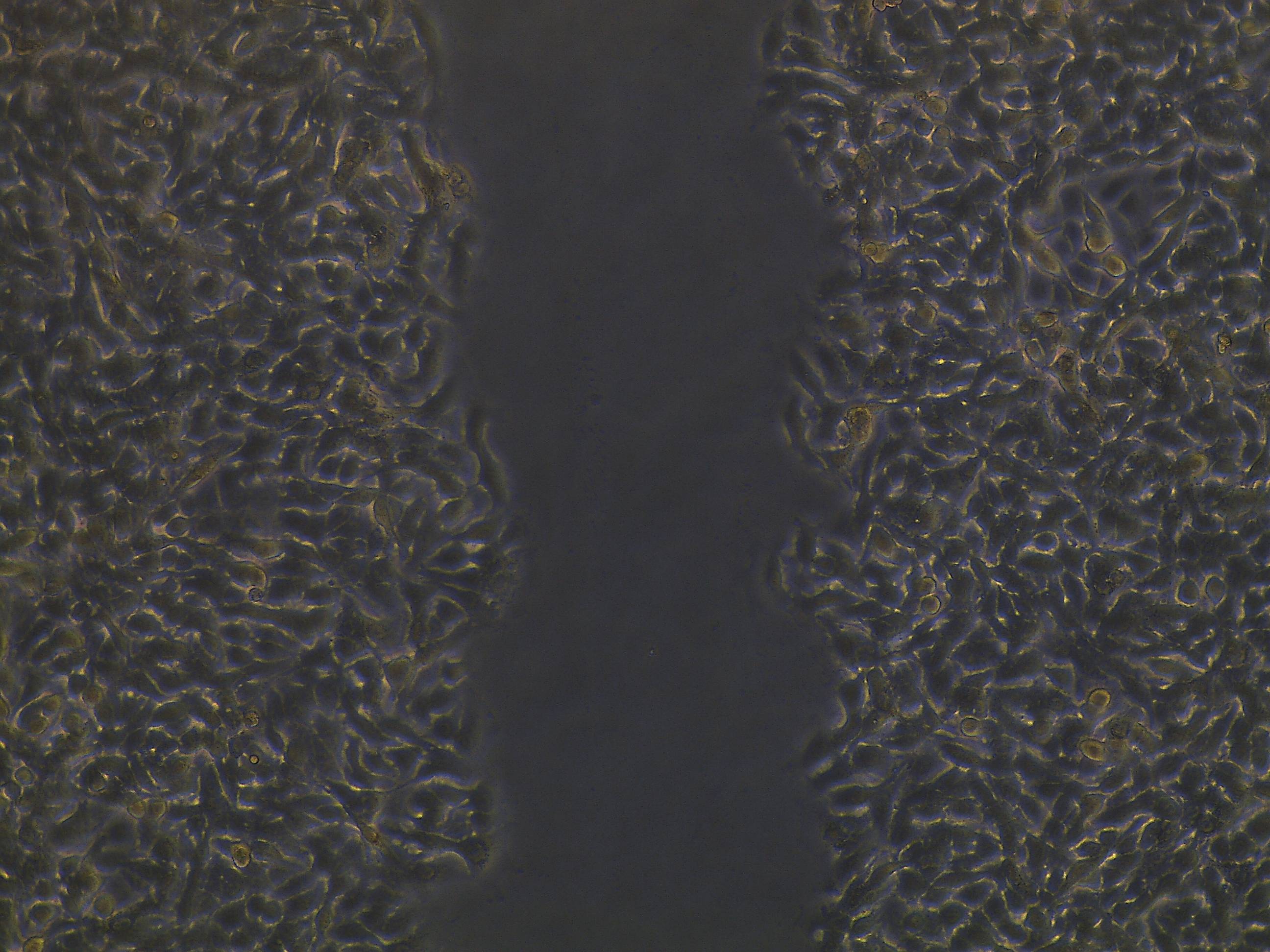

Supplement: Supplementary file 10 [file DataSheet3.zip › the raw images to Figures 14A, 14B, AND 14C/Wound Healing Assay/A549/24h(NC).jpg]

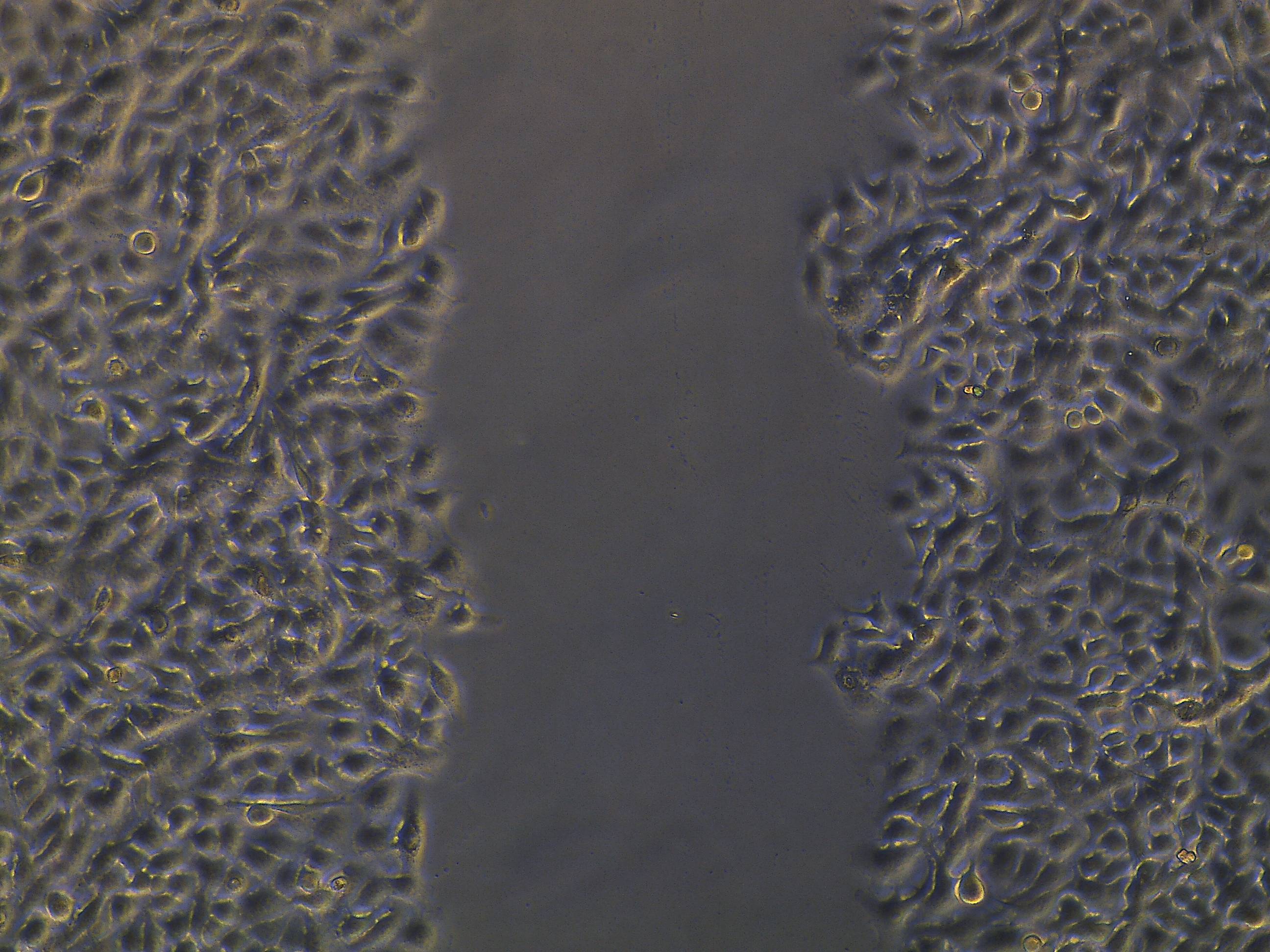

Supplement: Supplementary file 10 [file DataSheet3.zip › the raw images to Figures 14A, 14B, AND 14C/Wound Healing Assay/A549/24h(si-MTCH2-2).jpg]

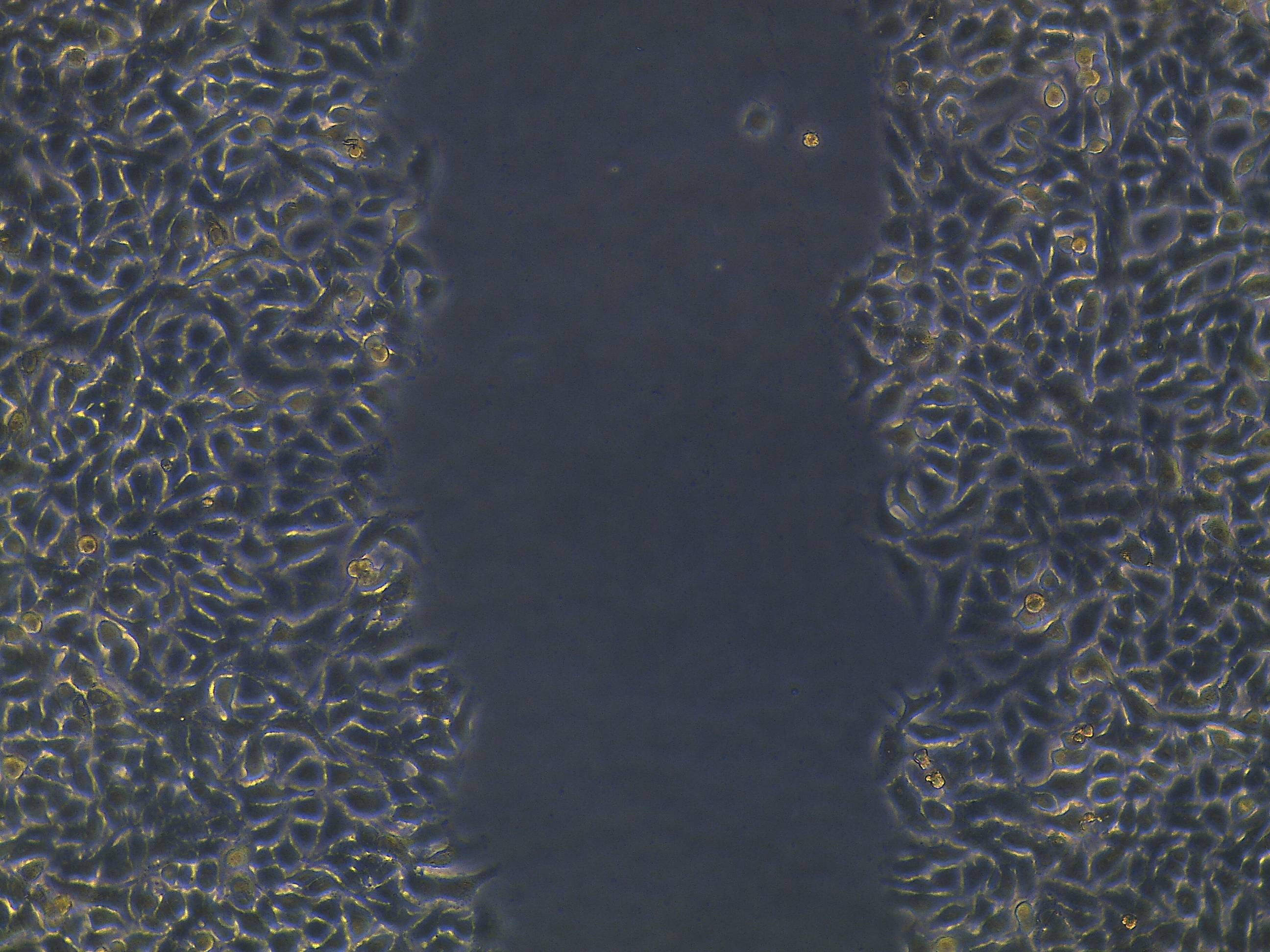

Supplement: Supplementary file 10 [file DataSheet3.zip › the raw images to Figures 14A, 14B, AND 14C/Wound Healing Assay/A549/24h(si-MTCH2-3).jpg]

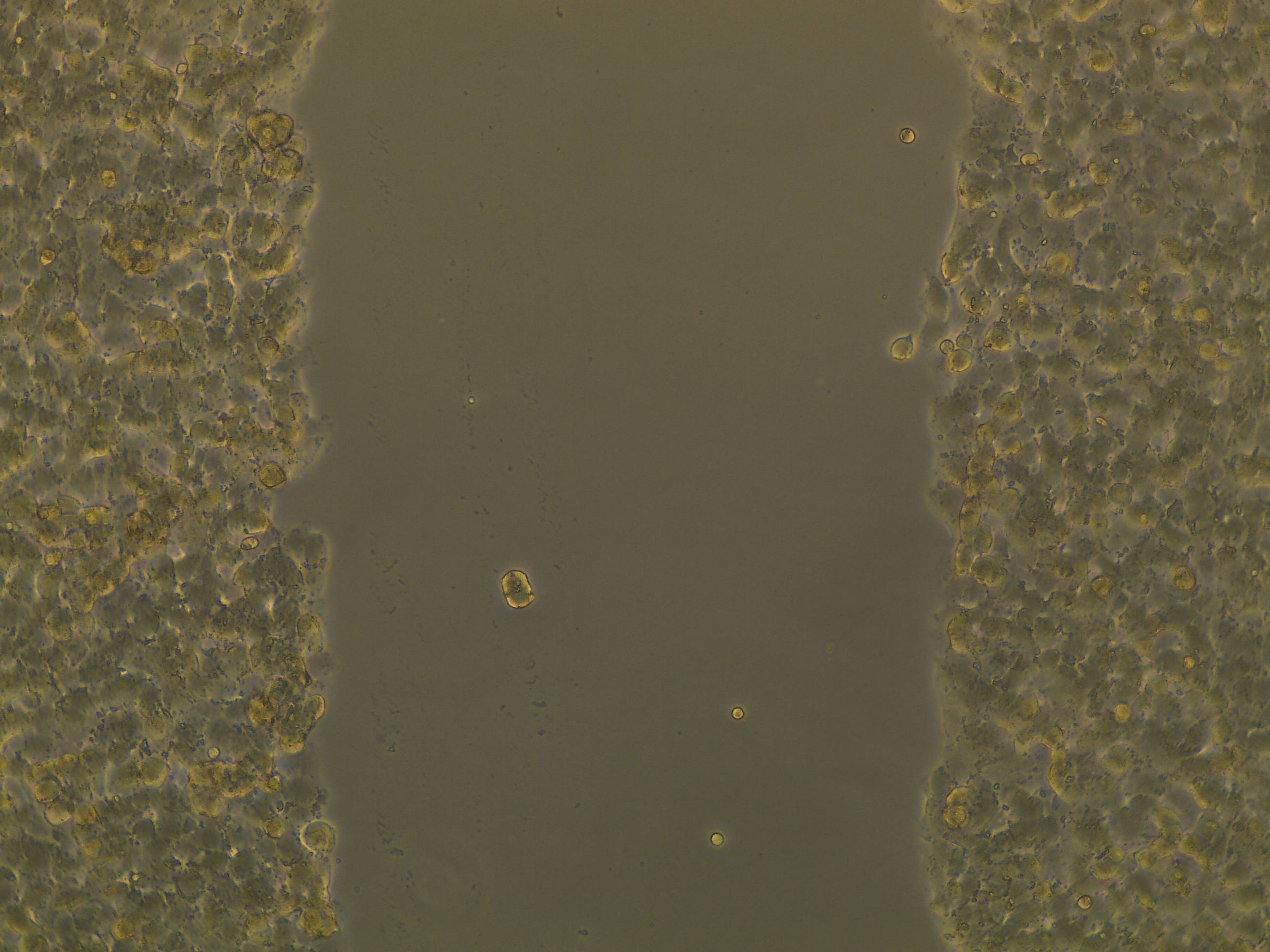

Supplement: Supplementary file 10 [file DataSheet3.zip › the raw images to Figures 14A, 14B, AND 14C/Wound Healing Assay/H1299/0h(NC).jpg]

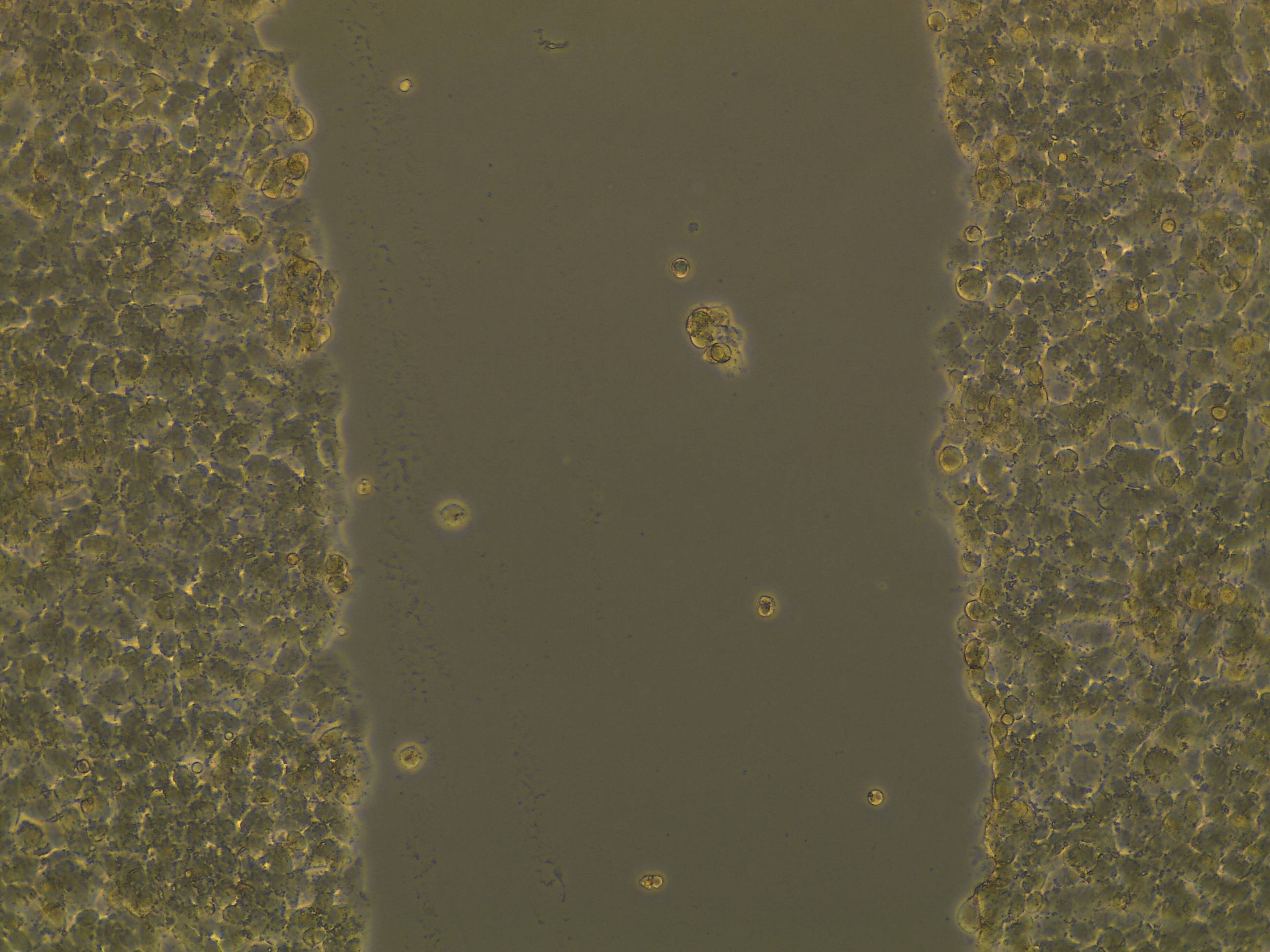

Supplement: Supplementary file 10 [file DataSheet3.zip › the raw images to Figures 14A, 14B, AND 14C/Wound Healing Assay/H1299/0h(si-MTCH2-2).jpg]

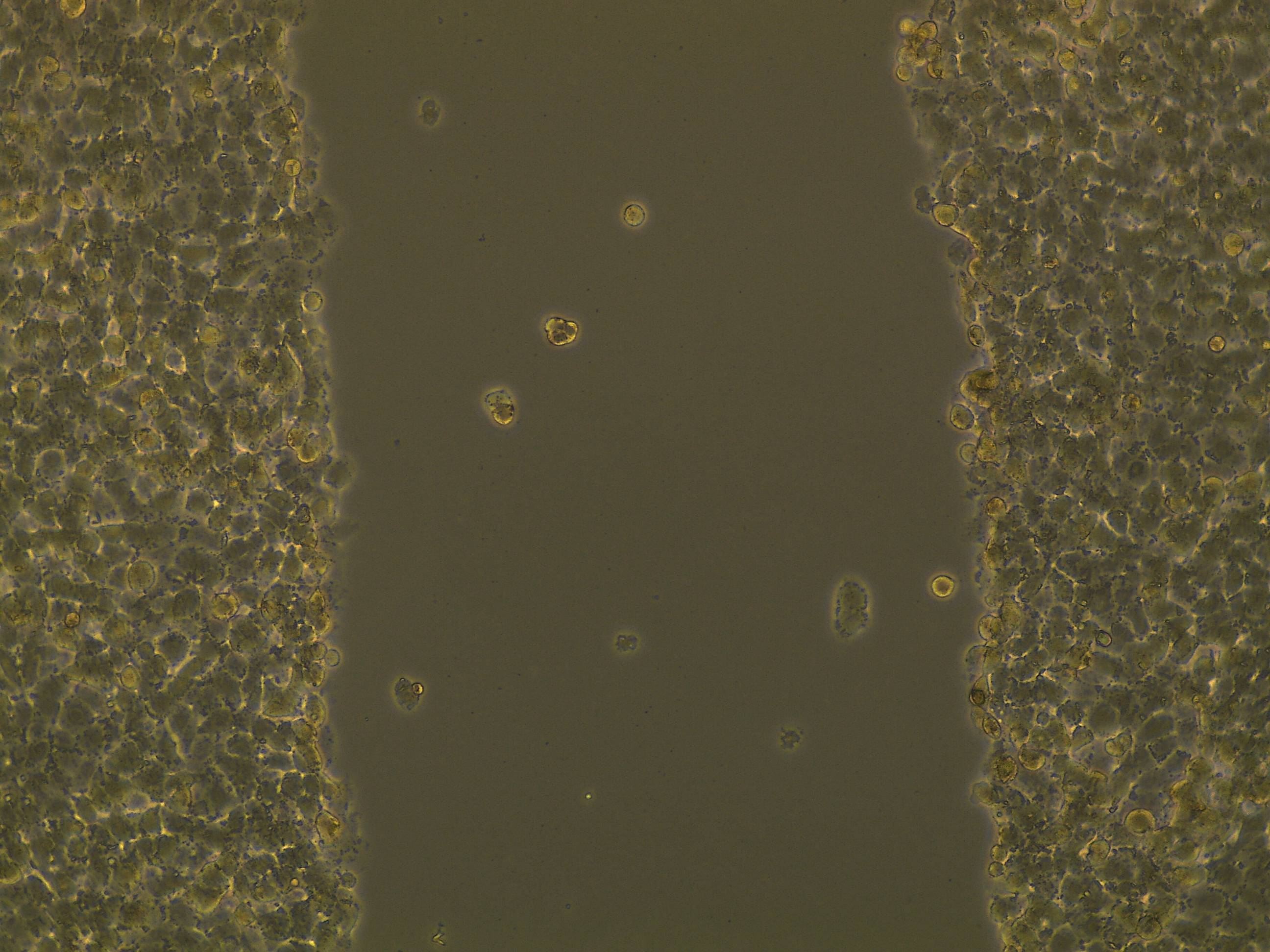

Supplement: Supplementary file 10 [file DataSheet3.zip › the raw images to Figures 14A, 14B, AND 14C/Wound Healing Assay/H1299/0h(si-MTCH2-3).jpg]

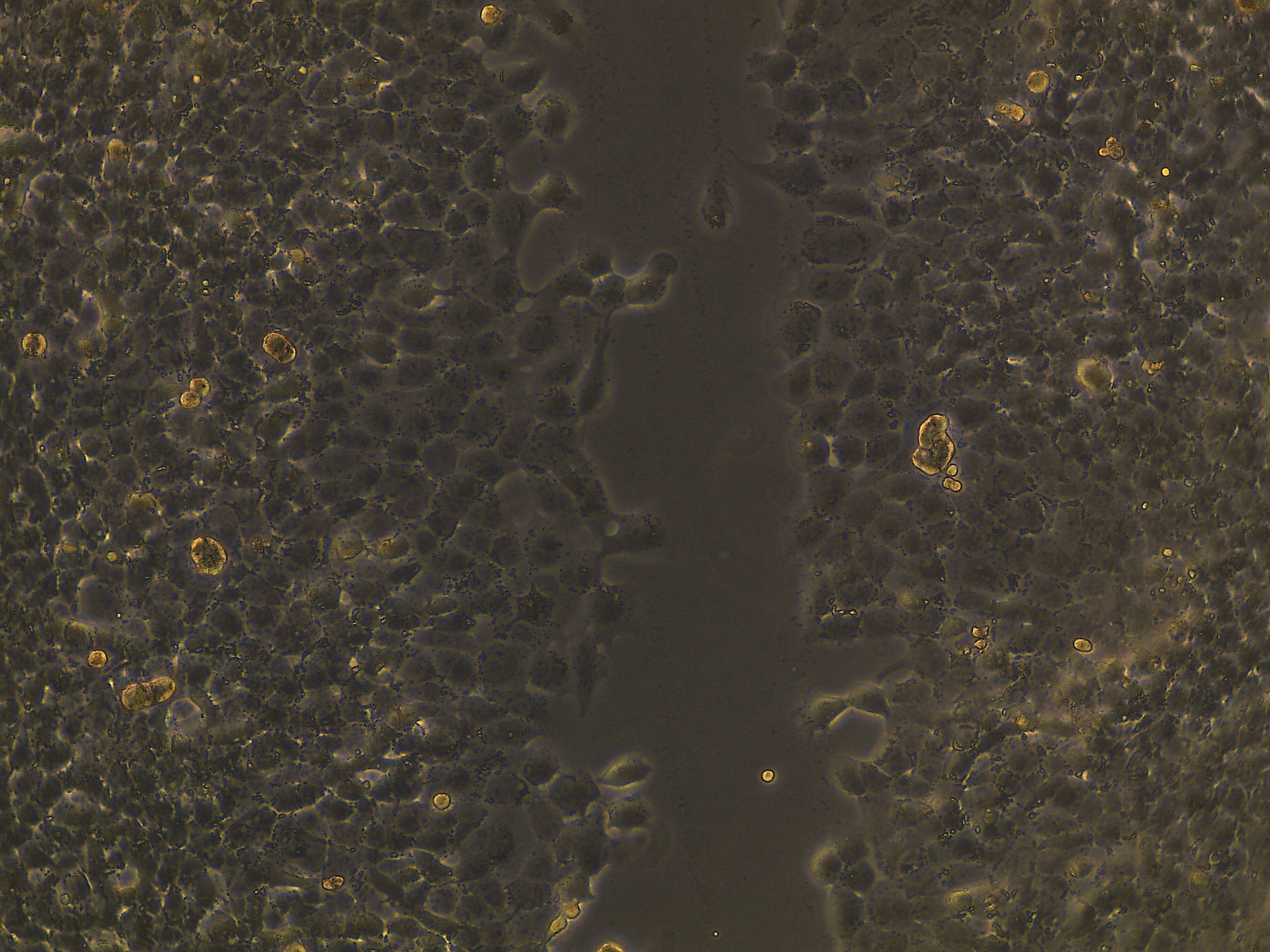

Supplement: Supplementary file 10 [file DataSheet3.zip › the raw images to Figures 14A, 14B, AND 14C/Wound Healing Assay/H1299/24h(NC).jpg]

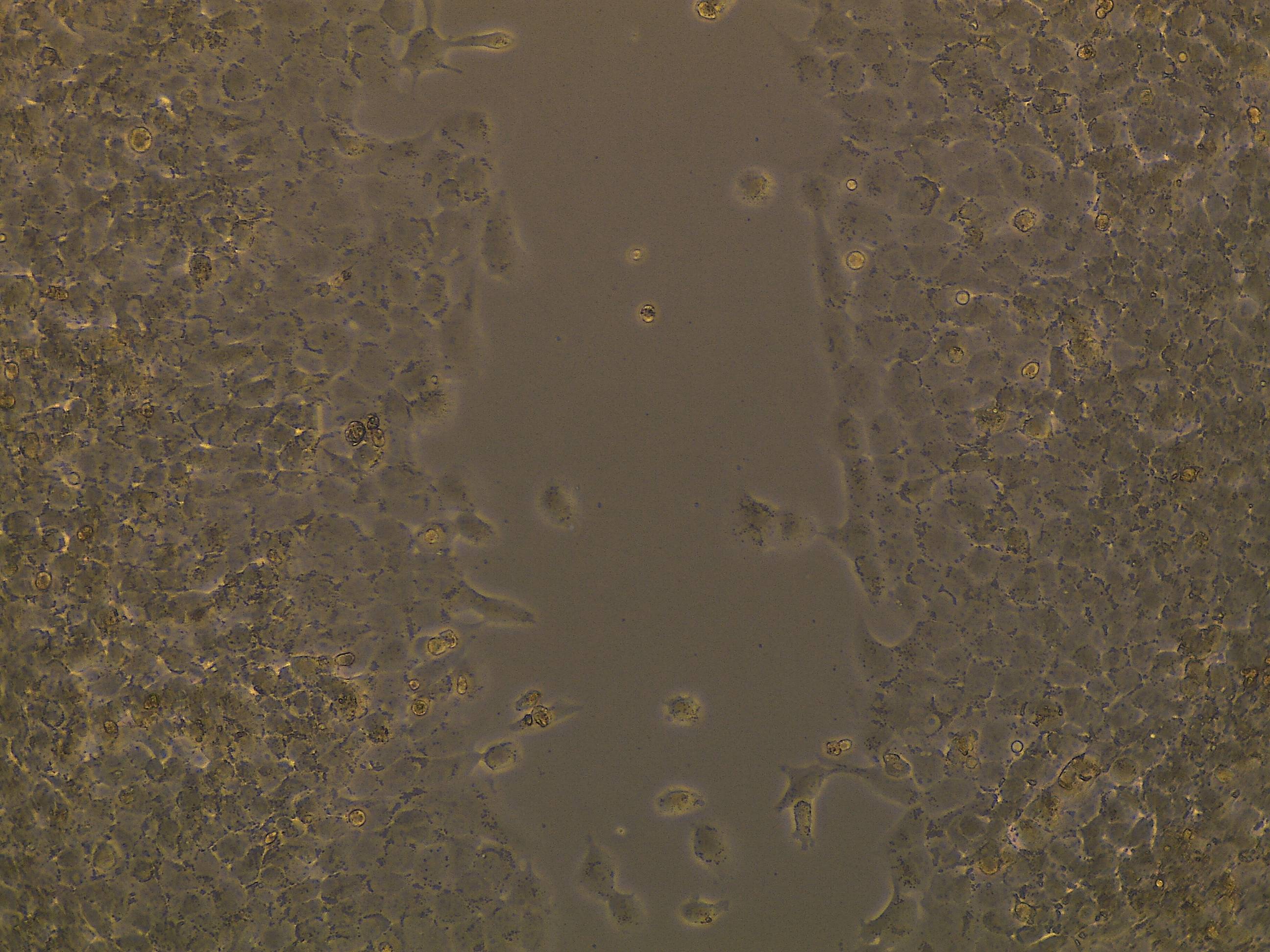

Supplement: Supplementary file 10 [file DataSheet3.zip › the raw images to Figures 14A, 14B, AND 14C/Wound Healing Assay/H1299/24h(si-MTCH2-2).jpg]

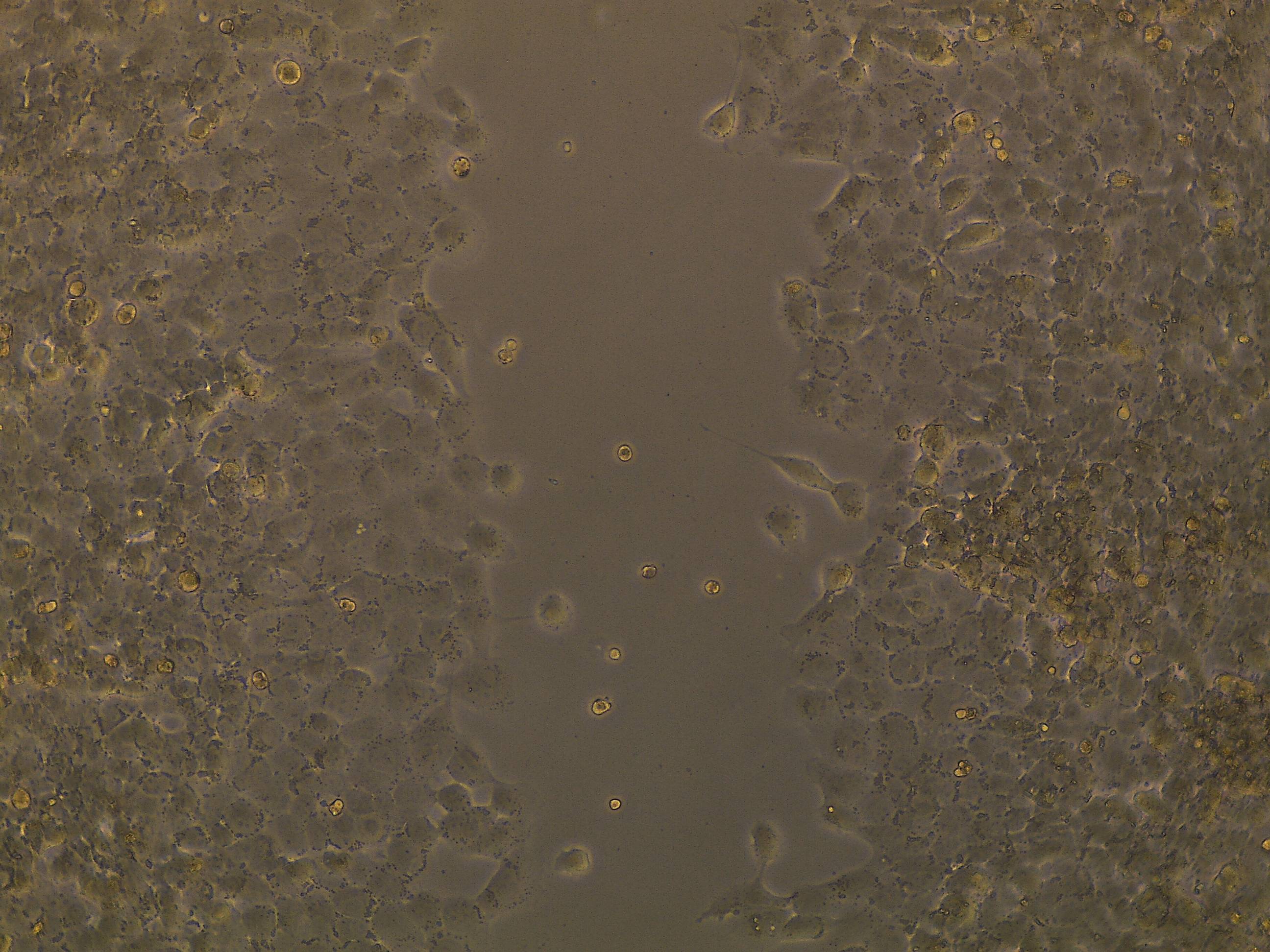

Supplement: Supplementary file 10 [file DataSheet3.zip › the raw images to Figures 14A, 14B, AND 14C/Wound Healing Assay/H1299/24h(si-MTCH2-3).jpg]

MTCH2


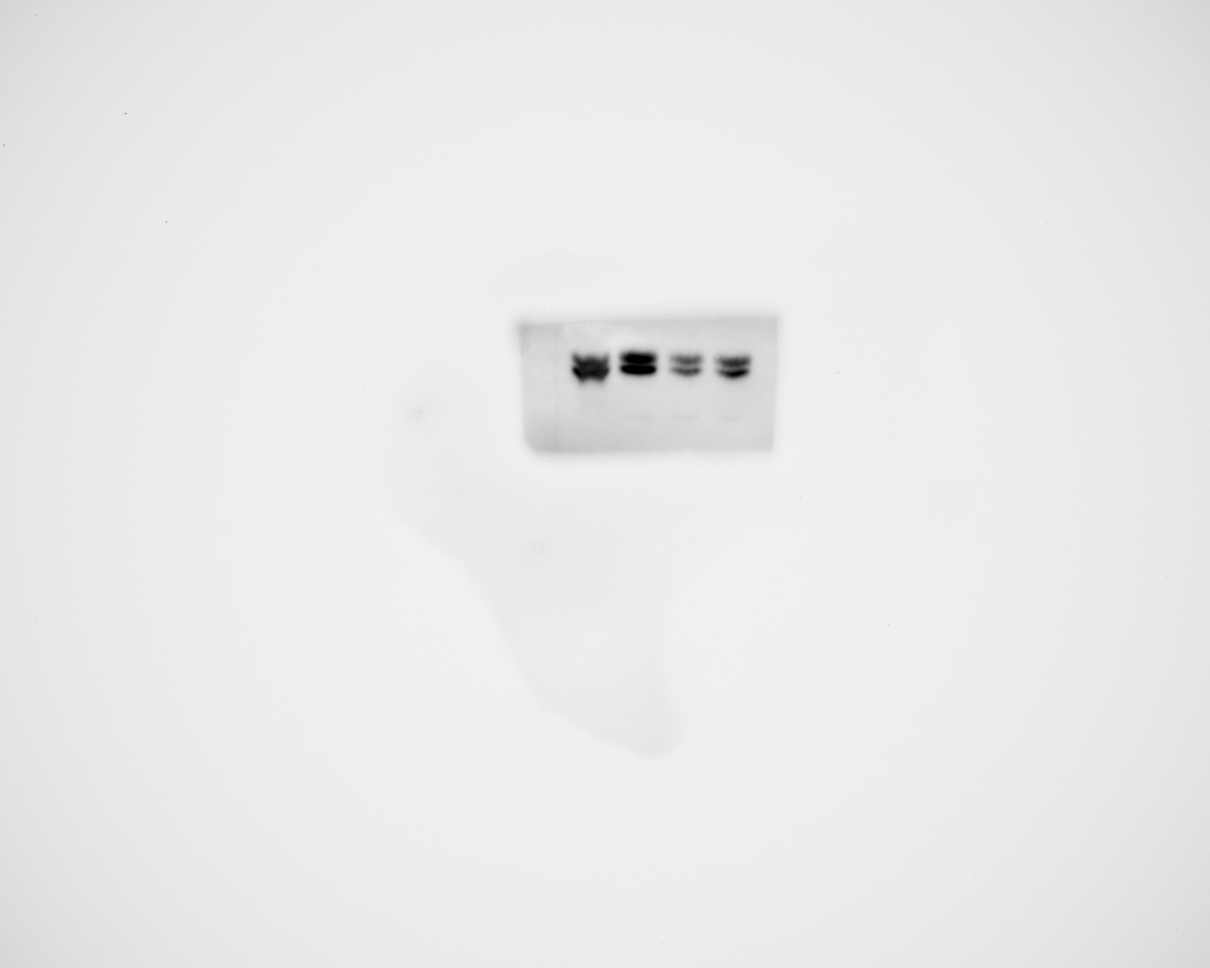

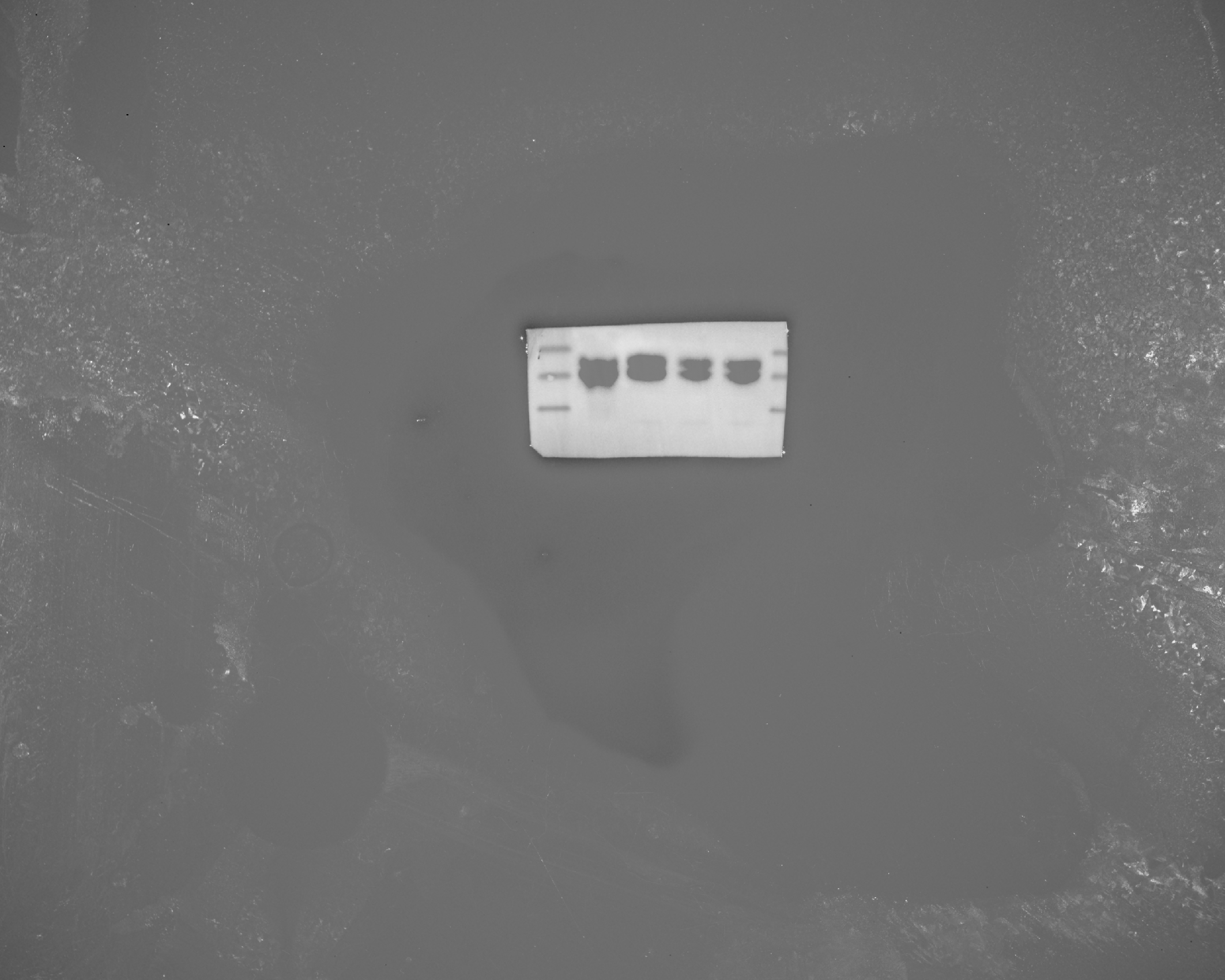


255

40

35

tubulin


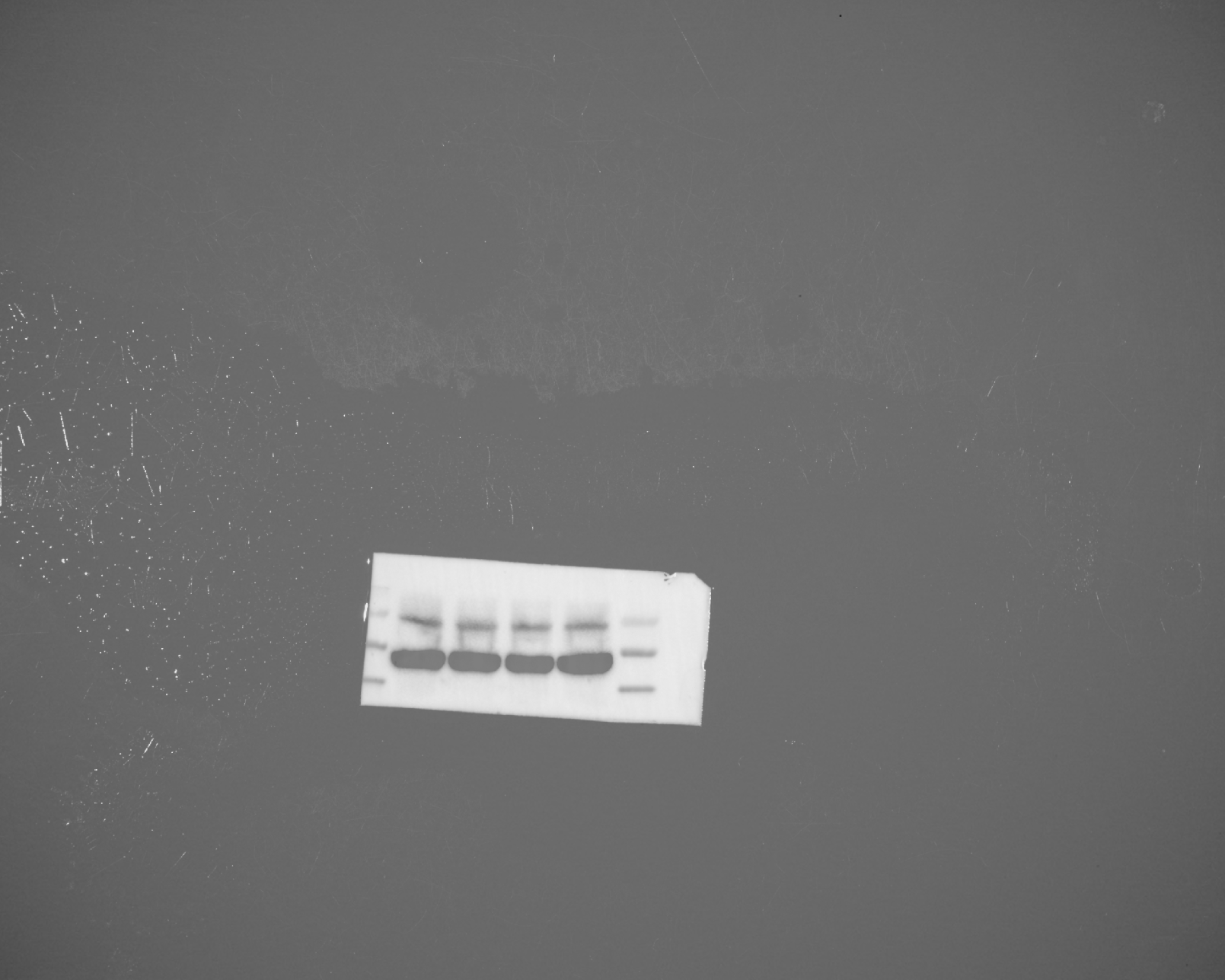

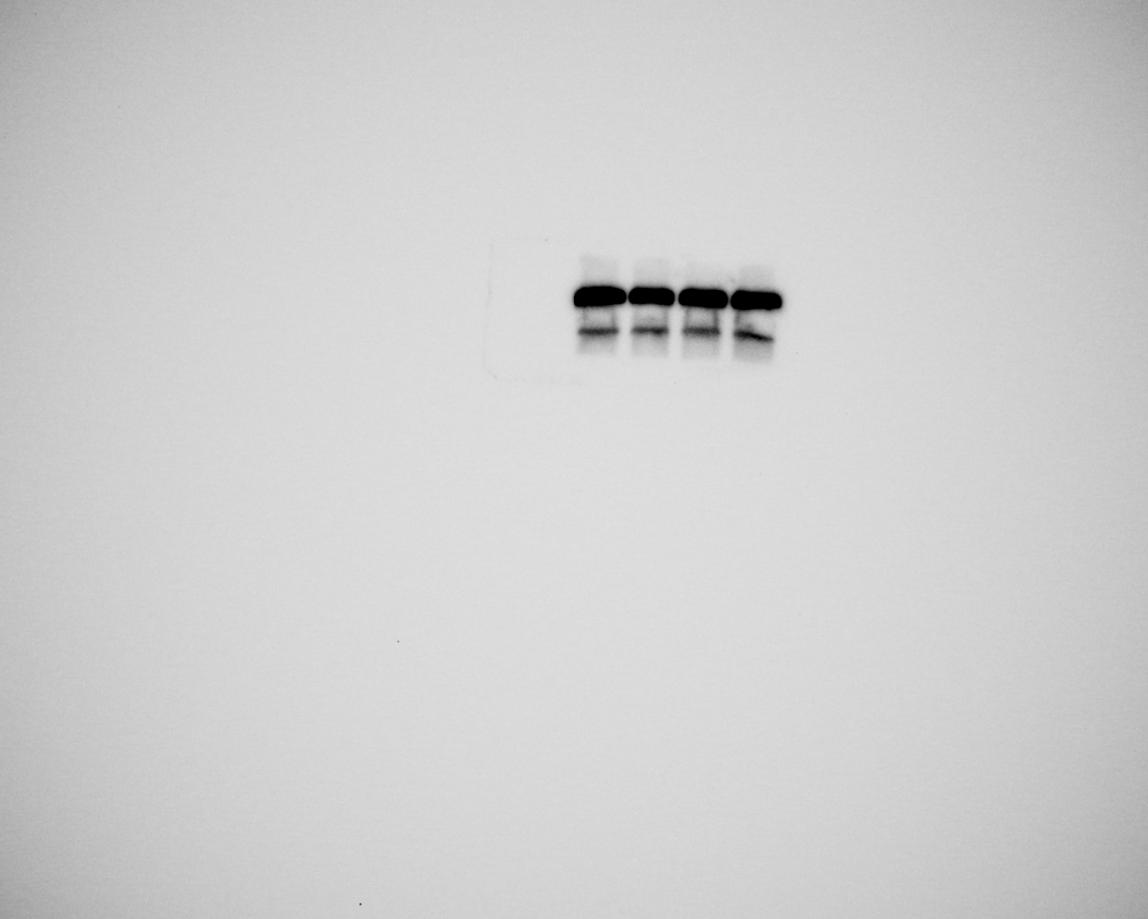


55

40

70

tubulin


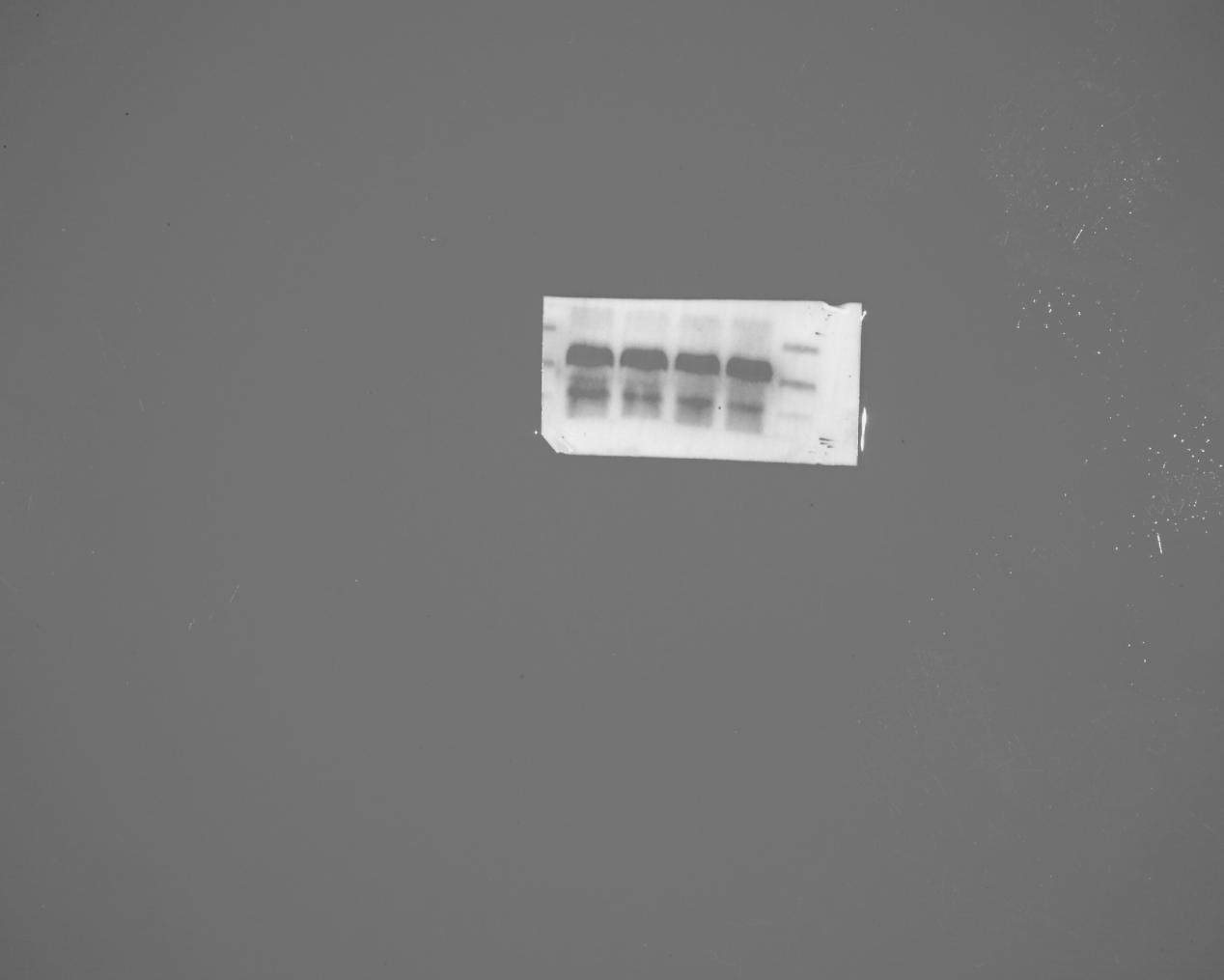

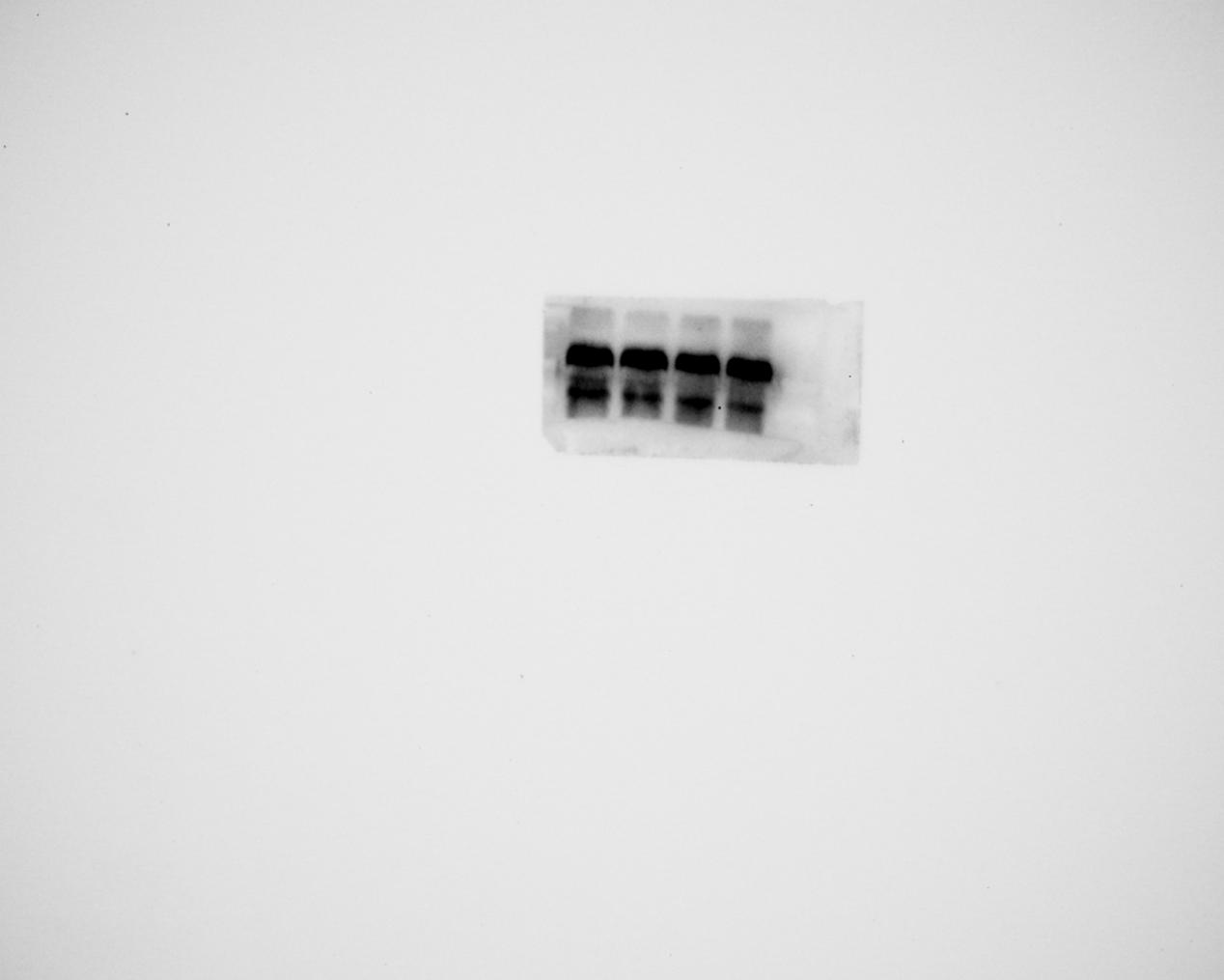


MTCH2


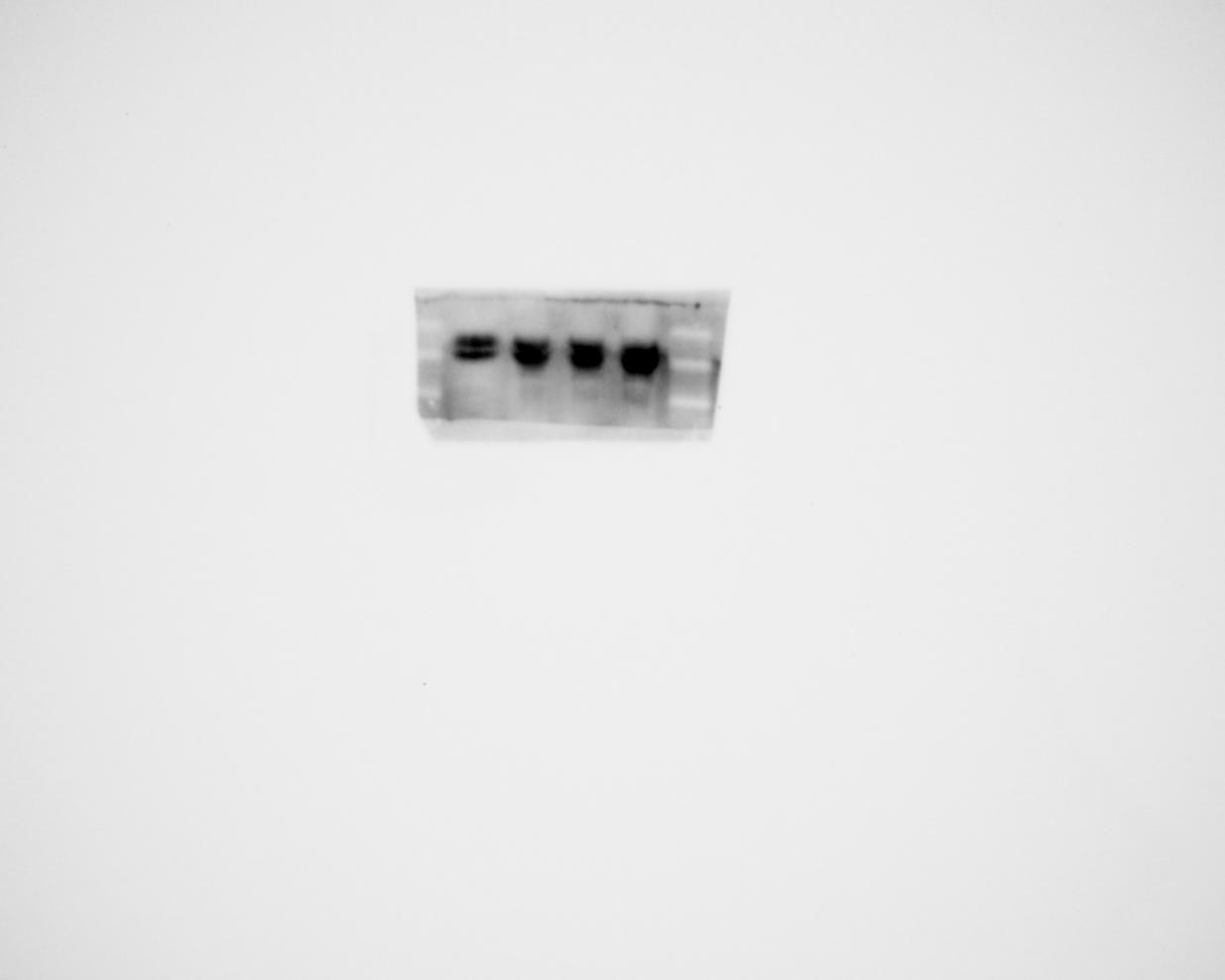

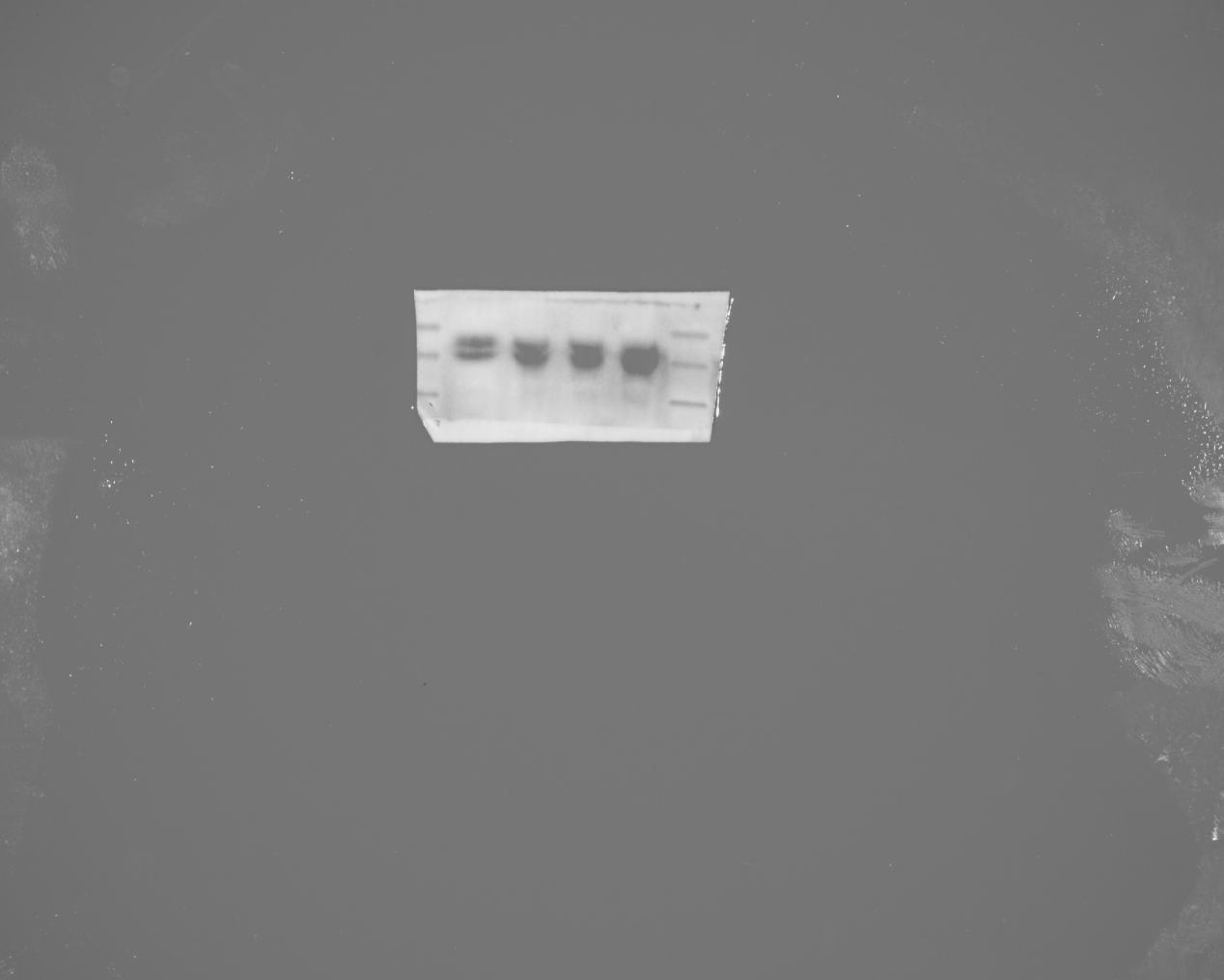


MTCH2


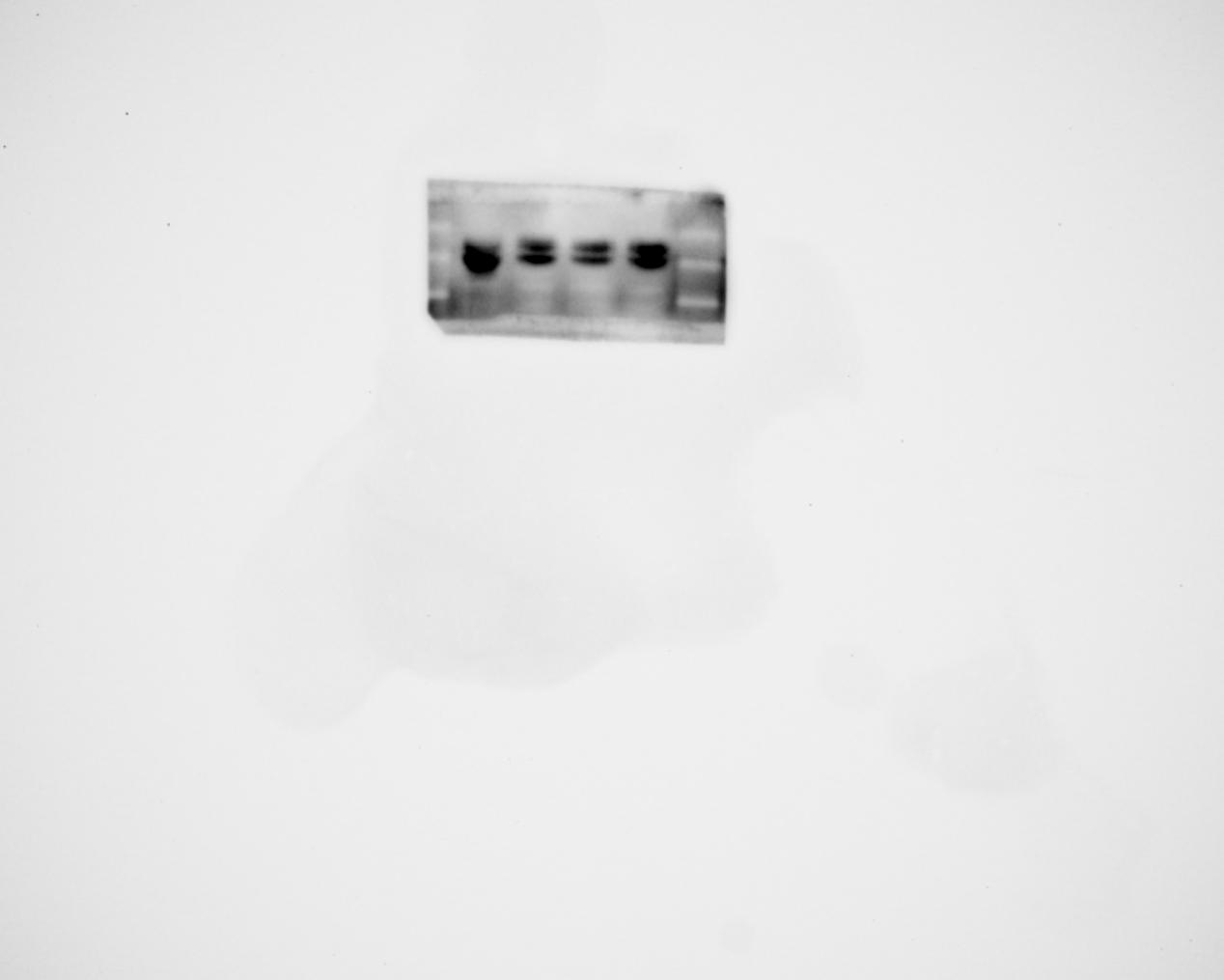


MTCH2


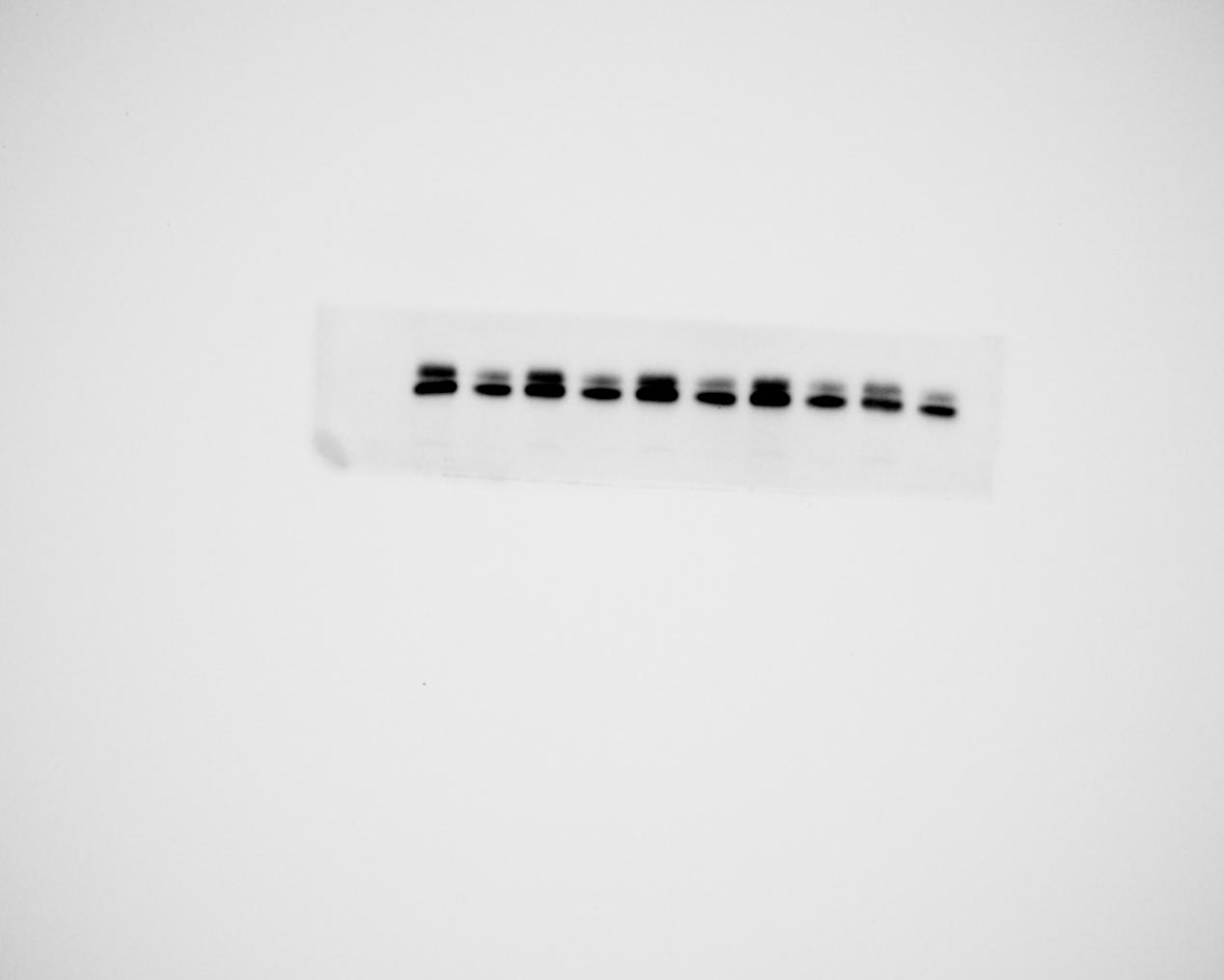

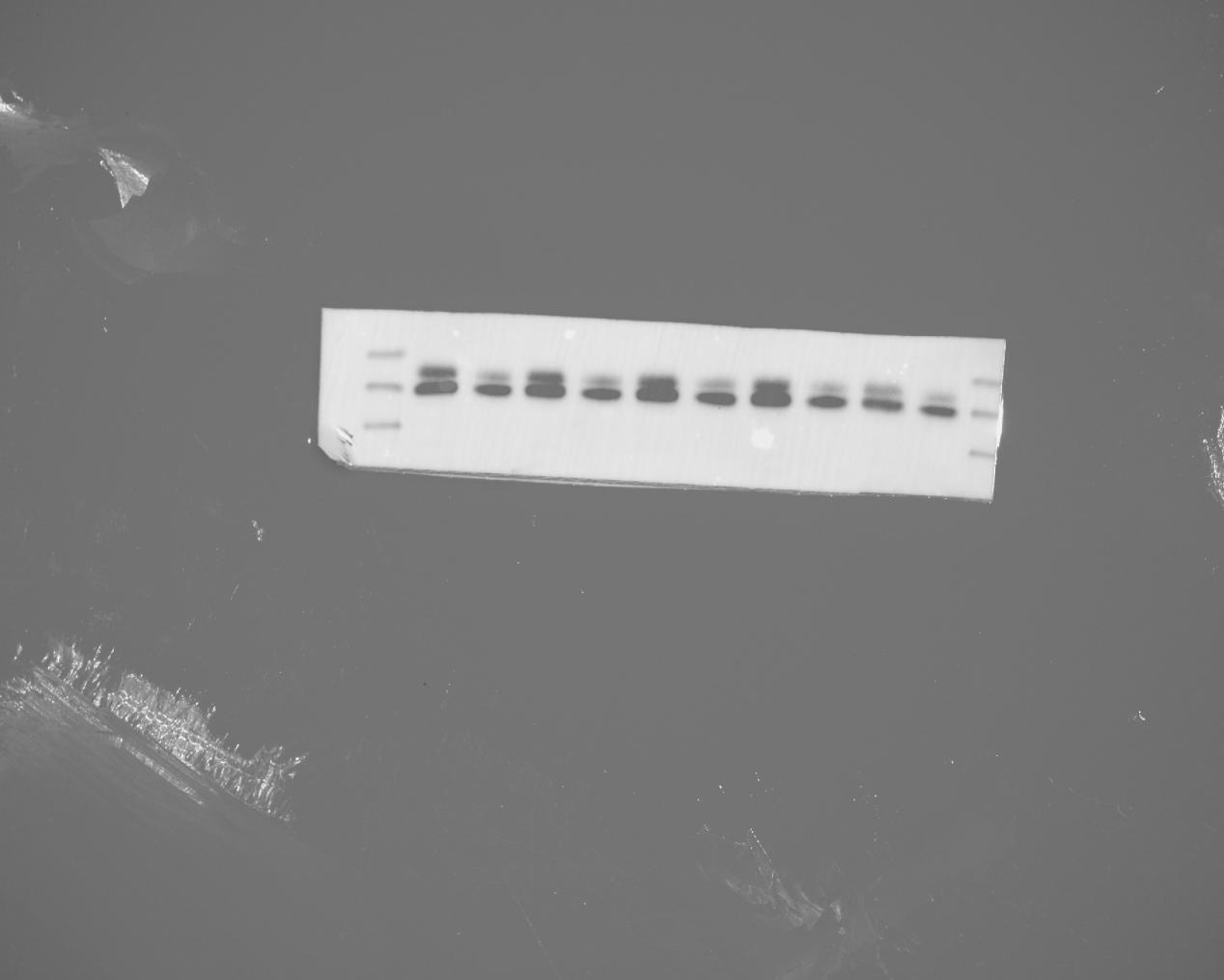


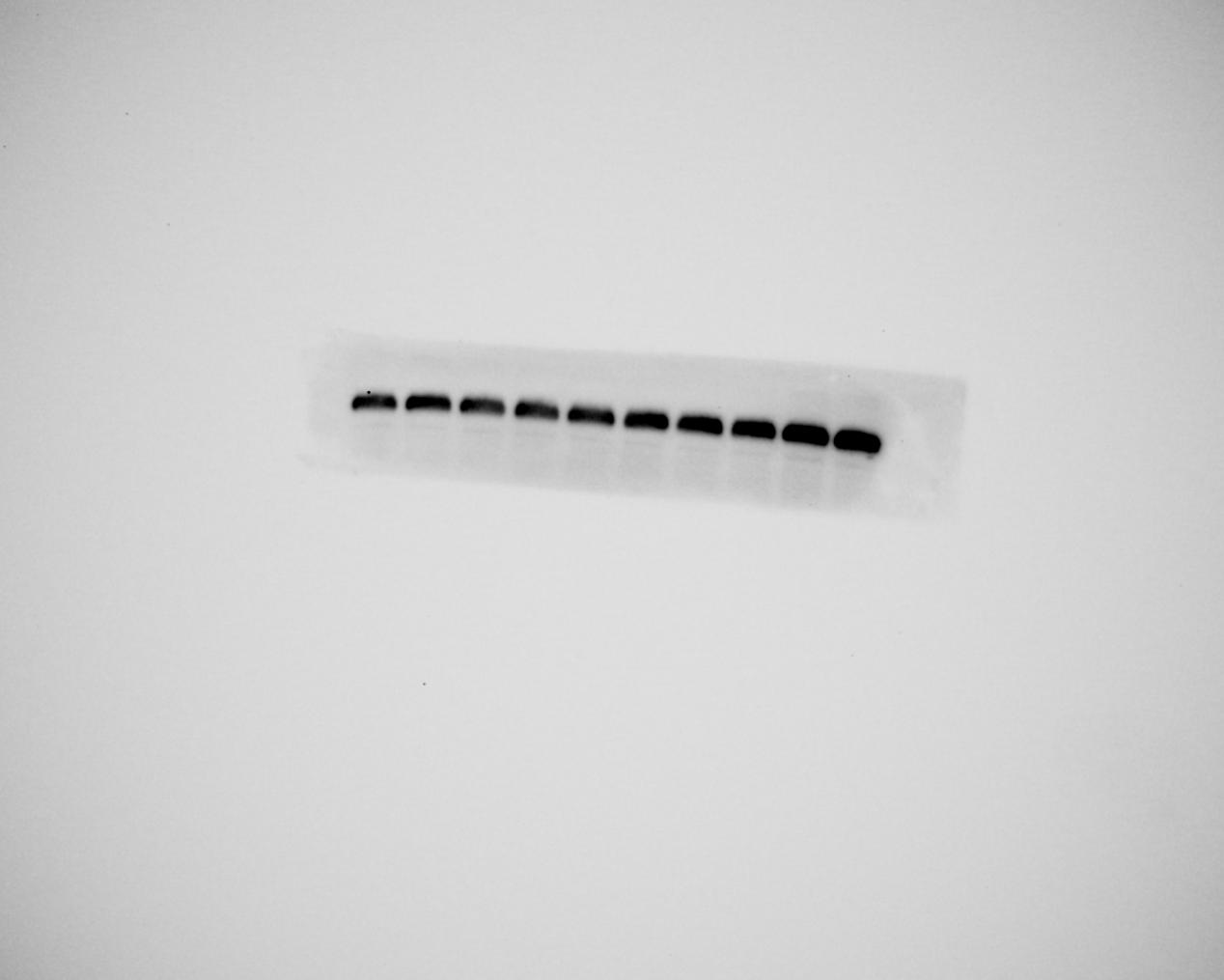

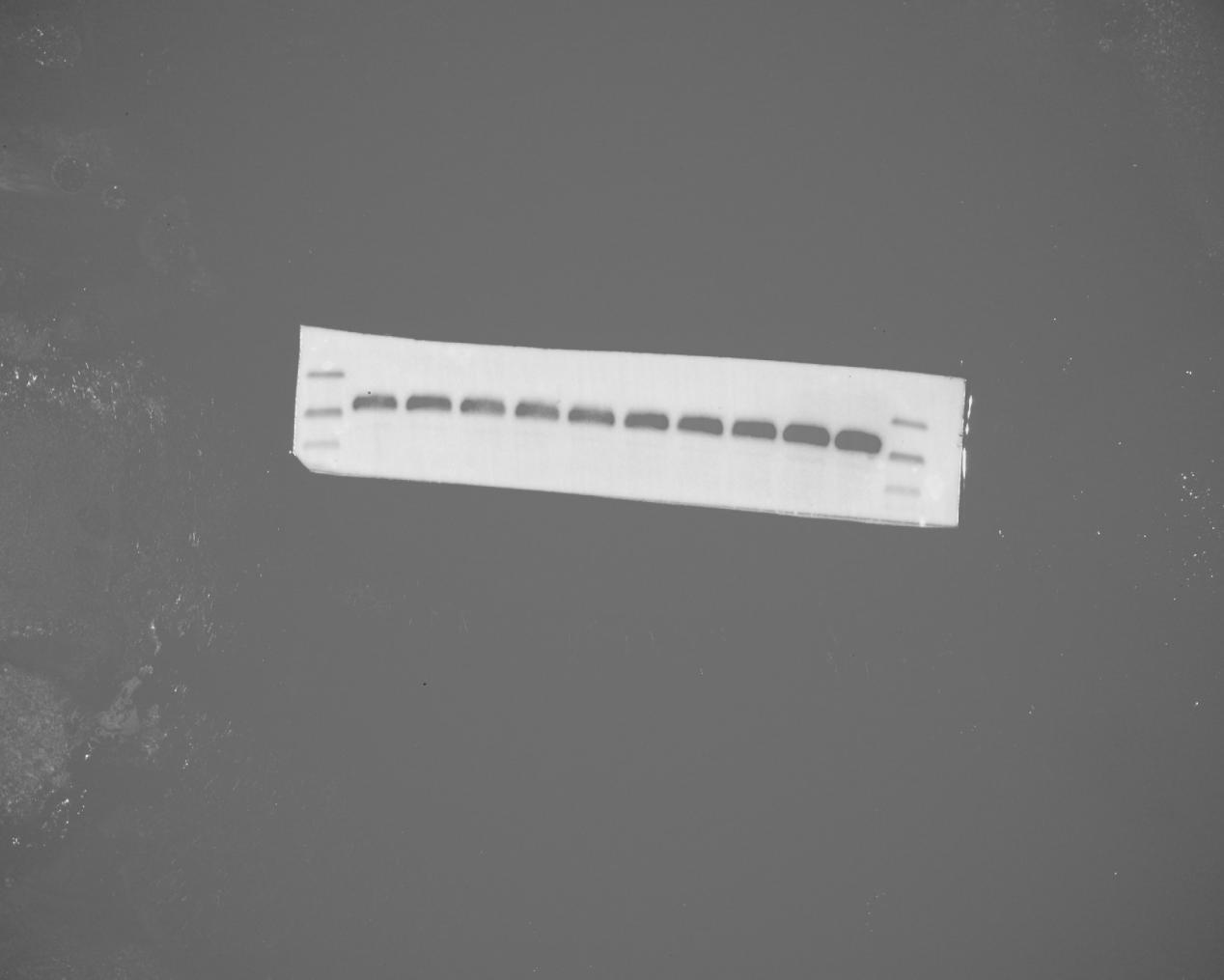


tubulin

Supplement: Supplementary file 12 [file Table2.docx]
